# Relative Efficacy, Effectiveness and Safety of Newer and/or Enhanced Seasonal Influenza Vaccines for the Prevention of Laboratory‐Confirmed Influenza in Individuals Aged 18 years and Over: Update of a Systematic Review

**DOI:** 10.1002/rmv.70020

**Published:** 2025-02-24

**Authors:** Mona Askar, Karam Adel Ali, Madeleine Batke, Timo Brugger, Annika Falman, Anna Hayman Robertson, Jaime Jesús Pérez, Kari Johansen, Jorgen de Jonge, Tyra Grove Krause, Wiebe Külper‐Schiek, Joerg J. Meerpohl, Angeliki Melidou, Hanna Nohynek, Carmen Olmedo, Kate Olsson, Ioanna Pavlopoulou, Vanessa Piechotta, Johanna Rubin, Johanna Schlaberg, Christine Schmucker, Waldemar Siemens, Jan Stratil, Veronika Učakar, Ole Wichmann, Thomas Harder

**Affiliations:** ^1^ Immunisation Unit Robert Koch Institute Berlin Germany; ^2^ European Centre for Disease Prevention and Control Solna Sweden; ^3^ Faculty of Medicine Institute for Evidence in Medicine Medical Center ‐ University of Freiburg University of Freiburg Freiburg Germany; ^4^ Cochrane Germany Cochrane Germany Foundation Freiburg Germany; ^5^ Division of Infection Control Norwegian Institute of Public Health Oslo Norway; ^6^ General Directorate of Public Health and Addictions IMIB‐Arrixaca Murcia University Region of Murcia Spain; ^7^ Vaccination Programme General Directorate of Public Health Ministry of Health Madrid Spain; ^8^ Public Health Agency of Sweden Solna Sweden; ^9^ Center for Infectious Disease Control National Institute for Public Health and the Environment Bilthoven The Netherlands; ^10^ Statens Serum Institut Copenhagen Denmark; ^11^ Finnish Institute for Health and Welfare Helsinki Finland; ^12^ Pediatric Research Laboratory School of Health Sciences Faculty of Nursing National & Kapodistrian University of Athens Goudi Greece; ^13^ National Advisory Committee on Immunisation Hellenic Ministry of Health Athens Greece; ^14^ Centre for Communicable Diseases National Institute of Public Health Ljubljana Slovenia

## Abstract

We performed an update (last search: 24 July 2023) of a systematic review on relative efficacy/effectiveness (rVE) and safety of newer/enhanced seasonal influenza vaccines in comparison with standard influenza vaccine or in head‐to‐head comparison. Eligible studies investigated adults aged ≥ 18 years, analysed the MF59‐adjuvanted or high‐dose or cell‐based or recombinant or mRNA‐based influenza vaccine and reported rVE or safety in randomised controlled trials (RCT) or non‐randomised studies of interventions (NRSI). Of 1561 new entries identified, 17 studies were included. Together with 42 studies identified in the previous primary review they added up to 59 studies, all comparing newer/enhanced with standard seasonal influenza vaccines. Relative VE against laboratory‐confirmed influenza was −30% (95%CI: −146% to 31%) to 88% (51%–100%; 7 NRSI) for the MF59‐adjuvanted vaccine (low certainty of evidence, CoE); 24.2% (9.7%–36.5%; 1 RCT) and −9% (−158% to 54%) to 19% (−27% to 48%; 1 NRSI) for the high‐dose vaccine (moderate CoE); −5.8% (−36.1% to 17.7%) to 21.4% (−7.3% to 42.4%; 2 NRSI) for the cell‐based vaccine (low CoE); 30% (10%–47%; 1 RCT) and 3% (−31% to 28%) to 19% (−27% to 48%; 1 NRSI) for the recombinant vaccine (moderate CoE), respectively. Relative VE against laboratory‐confirmed influenza‐related hospitalisation was 59.2% (14.6%–80.5%; 1 NRSI) for the MF59‐adjuvanted (moderate CoE); 27% (−1 to 48%; 1 NRSI) for the high‐dose (low CoE); 8.5% (−75.9% to 52.3%; 1 NRSI) for the cell‐based (low CoE); −7.3% (−52.1% to 24.4%) to 16.3% (−8.7% to 35.5%; 1 RCT) for the recombinant vaccine. No increased risk of serious adverse events was detected for any vaccine (12 RCT, 7 NRSI; low CoE). While all have a favourable safety profile, evidence on rVE of newer/enhanced vaccines is still limited, warranting further studies.

## 1 Introduction

Influenza is a respiratory infectious disease that spreads globally through seasonal epidemics and occasional pandemics [1, 2]. In humans, the most commonly observed types are influenza A and B. The burden of seasonal influenza is influenced by various factors such as the circulating strain(s), antigenic drift, immunity in the population after previous infection, vaccination and the extent of vaccination coverage [3].

Vaccination is the most effective mean to prevent influenza virus infections. However, the effectiveness of influenza vaccines varies from season to season and is influenced by a number of factors [1]. Especially among population groups that have a higher risk for a severe disease outcome, like elderly and persons with immunocompromising conditions, the response to standard influenza vaccine is reduced [4]. Newer and enhanced influenza vaccines have been developed in an attempt to further improve vaccine effectiveness [5]. In this context, NITAGs face the challenge to decide whether any of these enhanced influenza vaccines are necessary for older adults, or whether one product stands out in terms of relative vaccine effectiveness.

A systematic review on the efficacy, effectiveness and safety of newer and enhanced seasonal influenza vaccines covered data up to 7 February 2020 [4, 6, 7, 8, 9]. The aim of that work (herein referred to as the primary review) was to assess and synthesise the available evidence on the MF59‐adjuvanted, cell‐based, high‐dose, and recombinant haemagglutinin (HA) influenza vaccines for the prevention of laboratory‐confirmed influenza in individuals aged 18 years or older. While the safety profiles of these vaccines were generally consistent with expectations based on their individual compositions and were well‐tolerated, the overall evidence on the efficacy and effectiveness appeared limited at that time. However, at the date of last search that primary review identified a number of potentially relevant studies that were still ongoing. This emphasised the need to update the systematic review after a short period of time and to include also new technology developments, such as the messenger RNA (mRNA)‐based influenza vaccine.

The aim of this systematic review update was to review, assess and synthesise a joint body of evidence consisting of the most recent literature search (performed on 24 July 2023) in amalgamation with studies from the primary review, that met the inclusion criteria of this review update, on newer and enhanced inactivated seasonal influenza vaccines for the prevention of laboratory‐confirmed influenza in individuals ≥ 18 years of age in comparison to standard influenza vaccines [4].

## 2 Methods

The protocol for this systematic review has been developed following the Preferred Reporting Items for Systematic Review and Meta‐Analysis Protocols (PRISMA‐P) 2020 statement (prisma‐statement.org) [10]. This review is registered in the International Prospective Register of Systematic Reviews (PROSPERO) under the registration number CRD42023441114. (see Supporting Information S1: Appendix G for differences to study protocol).

### 2.1 Eligibility Criteria

We considered randomised controlled trials (RCTs) with randomisation either at the individual or cluster level. Non‐randomised studies of interventions (NRSI) were considered as long as they had a control group. We considered studies performed in subjects ≥ 18 years irrespective of health status or setting. To be eligible, studies had to investigate the performance of at least one of the following newer and enhanced seasonal tri‐ or quadrivalent inactivated influenza vaccines:

- adjuvanted vaccine;
- high‐dose vaccine;
- cell‐based vaccine;
- recombinant HA vaccine;
- mRNA‐based vaccine.

Valid comparators were tri‐ or quadrivalent standard influenza vaccines or one of the above‐mentioned newer and/or enhanced seasonal tri‐ or quadrivalent influenza vaccines (head‐to‐head comparison between newer and/or enhanced vaccines).

We assessed the following primary rVE outcomes:

- Laboratory‐confirmed influenza (a positive laboratory diagnosis by PCR, virus culture or antigen detection);
- Influenza‐related hospitalisation (laboratory‐confirmed by PCR, virus culture or antigen detection);
- Influenza‐related death (laboratory‐confirmed by PCR, virus culture or antigen detection).

The following primary safety outcomes were assessed:

- Serious adverse events (requiring intervention to prevent disability or permanent damage, resulting in disability or permanent damage, initial or prolonged hospital care, congenital anomaly/birth defect, life‐threatening, or resulting in death).

In addition, we considered the following secondary outcomes:

- Influenza related intensive care unit (ICU) admissions (laboratory‐confirmed by PCR, virus culture or antigen detection);
- Influenza associated pneumonia/lower respiratory tract disease (laboratory‐confirmed by PCR, virus culture or antigen detection);
- Influenza‐associated cardiovascular disease (laboratory‐confirmed by PCR, virus culture or antigen detection);
- Influenza‐like illness (ILI) (symptoms of influenza only). Internationally accepted case definitions to be used (e.g. WHO, US CDC, EU);
  — Systemic adverse events (headache, fever);
  — Local adverse events (pain, swelling);
  — Adverse pregnancy outcomes after vaccination during pregnancy: spontaneous abortion, foetal death, stillbirth, preterm birth (less than 37 weeks), pre‐eclampsia and eclampsia;
- Adverse neonatal outcomes after vaccination during pregnancy: congenital malformations (minor and major), neonatal death and small‐for‐gestational‐age.

### 2.2 Search Strategy

Comprehensive systematic literature searches were conducted by following the recommendation of PRESS (Peer Review of Electronic Search Strategies). The full electronic search strategies were peer‐reviewed by an information specialist (IK) and validated by checking whether the strategy identified studies already known. For this update of a systematic review [4] a search for literature published after 1 January 2020 was conducted on 24 July 2023 [4]. No language filters were applied (see Supporting Information S1: Appendix A for the complete search strategies).

Searches for published studies were conducted in Medline (via Ovid) and Embase (via Ovid). Searches for ongoing studies or unpublished completed studies were performed in ClinicalTrials.gov (www.clinicaltrials.gov). We used relevant studies and/or systematic reviews to search for additional references via the Pubmed similar articles function (https://www.nlm.nih.gov/bsd/disted/pubmedtutorial/020_190.html) and forward citation tracking. Reference lists of included studies were reviewed and experts in the field were contacted to enquire about any further relevant studies or unpublished data that may not have been retrieved by the electronic searches. Further, a search in sources including websites of regulatory agencies (EMA and FDA) was conducted.

### 2.3 Data Collection and Analysis

Titles and abstracts of the citations identified by the searches were independently screened in duplicate by two out of three reviewers (M.A., M.B., W.K.). Full texts were also independently checked for eligibility in duplicate by two out of three reviewers (M.A., T.B., W.K.), and reasons for exclusions were documented. Any disagreement was resolved by consensus, moderated by a third reviewer (T.H.). The Covidence software was used for literature screening. Two pairs of review authors (M.A., T.B., A.F., JSchl) extracted study data. Disagreements in extracted data between the two reviewers were resolved through discussion until consensus was reached, involving a third reviewer if necessary. If necessary, authors of studies were contacted to provide any missing information or clarify any issues.

### 2.4 Assessment of Risk of Bias in Included Studies

Risk of bias was independently assessed by pairs of two authors (M.A., A.F., J.S., T.B.) by outcome level. Any disagreement was resolved by consensus, moderated by a third reviewer (T.H.). Bias in a RCT was evaluated according to the revised Cochrane risk of bias tool for randomised trials (RoB 2) [11, 12]. Bias in a NRSI was evaluated according to the ‘Risk of Bias in Non‐randomised Studies of Interventions’ tool (ROBINS‐I) [13]. Funnel plots for small study effects were constructed and visually inspected if ≥ 10 studies were available addressing the same outcome [14].

### 2.5 Measures of Treatment Effect

Relative vaccine estimates (in terms of efficacy or effectiveness) were expressed in percentage and calculated as follows: rVE = (1 – vaccine effect ratio) × 100. Thereby, we used the vaccine effect ratio as reported in the primary study (e.g. odds ratio [OR], risk ratio [RR], hazard ratio [HR], or incidence rate ratio [IRR]). The precision of the vaccine effect estimates was summarised with the corresponding 95% confidence interval (CI).

### 2.6 Assessment of Heterogeneity

Heterogeneity was evaluated and statistically quantified, where appropriate, based on *I* ^2^, chi square statistical test and visual inspection of the forest plot [15].

### 2.7 Data Synthesis

Where appropriate, meta‐analyses were conducted separately for each intervention (type of influenza vaccine) and separately for RCTs and NRSIs. Effect estimates were pooled by applying the inverse variance method. For meta‐analysis the fixed‐effects model was used as primary model. Random‐effects models were used as sensitivity analysis. For all meta‐analyses the Mantel‐Haenszel method was used. Meta‐analyses were conducted with RevMan Web.

### 2.8 Summary of Findings and Certainty of the Evidence Assessment

We used the GRADEpro GDT to create a summary of findings table and included the following primary outcomes: laboratory‐confirmed influenza; influenza‐related hospitalisation; influenza‐related death; serious adverse events. The certainty of evidence (CoE) for these patient‐relevant outcomes was assessed using the Grading of Recommendations, Assessment, Development and Evaluation (GRADE) approach considering both, evidence from RCTs and NRSI [16]. According to GRADE, the CoE can be categorised as very low, low, moderate and high.

## 3 Results

### 3.1 Description of Studies

**FIGURE 1:**
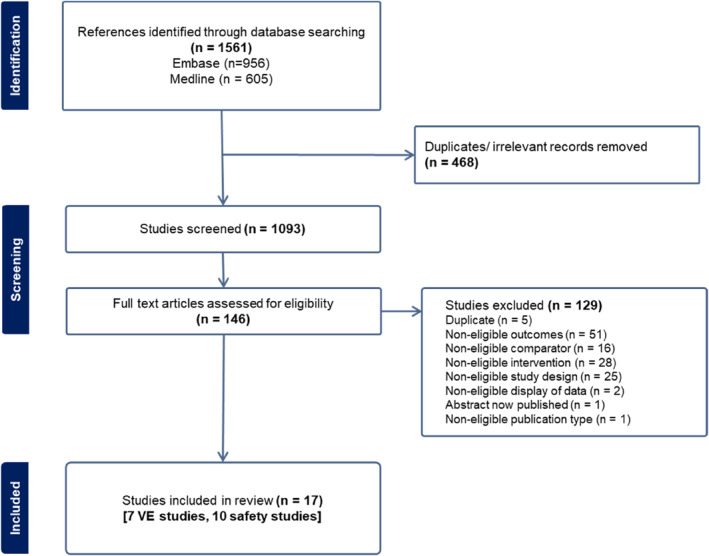
PRISMA flow diagram of the update search.

The literature search in the above‐mentioned sources resulted in 1561 records. After removing duplicates, 1093 records remained. During title and abstract screening, we judged 947 records to be irrelevant. From the remaining 146 records, we excluded 129 records during full‐text screening (see Supporting Information S1: Appendix B for records and exclusion reasons). Finally, we included 17 new studies in this update of the systematic review. Of those, 7 studies reported data on rVE and 10 studies provided data on safety. The flow of records is illustrated in Figure 1.

In the primary review [8, 9, 10, 11], a total of 110 studies were included. Of those, 10 studies reported on rVE and 32 studies on vaccine safety thereby met the inclusion criteria for this update review. As a result, the evidence body of this updated systematic review comprised 59 studies (42 studies from the primary review plus the 17 studies from this update review).

*Relative efficacy/effectiveness studies:* Details of studies that reported rVE data and were found in the update search are given in Table 1. We included one cluster‐RCT and 6 NRSI of which 2 were retrospective cohort studies and 4 had a test‐negative design. The studies were performed in the United States or Italy and had about 500 to ≥ 1 million participants. They reported rVE estimates for 1 to 4 influenza seasons between 2015/2016 and 2019/2020. In all included studies, newer/advanced influenza vaccines were compared to standard influenza vaccine; no head‐to‐head studies comparing two or more newer/enhanced against each other were identified. Two studies investigated the high‐dose influenza vaccine, one study the MF59‐adjuvanted vaccine, and another 2 studies reported rVE estimates for the cell‐based vaccine and the recombinant vaccine. No study reported on an mRNA‐based influenza vaccine. Three studies reported a total of 10 rVE estimates against laboratory‐confirmed influenza. The other four studies provided a total of 12 rVE estimates against laboratory‐confirmed influenza‐related hospitalisation. We did not identify rVE estimates for the other efficacy/effectiveness outcomes defined in the protocol.

**TABLE 1:** Key characteristic of relative vaccine efficacy/effectiveness studies included in the review update based on update search (7 studies).

Study	Intervention (comparison)	Study design	Country	Funding	Setting	Outcome	Influenza season	Population	*N* (vacc)	Mean age in years (SD)	Female sex %
Balasubramani 2020 [17]	HD‐3v (vs. SD‐3/4v)	Test‐negative	USA	Non‐industry funded	Outpatient	Influenza infection	2015–16, 2016–17, 2017–18, 2018–19	≥ 65 years	2993	HD 73.6 (6.9)	HD 62.3
										SD 73.3 (7.0)	SD 61.4
Doyle 2021 [18]	HD‐3v (vs. SD‐3/4v)	Test‐negative	USA	Non‐industry funded	Inpatient	Influenza‐related hospitalisation	2015–16, 2016–17	≥ 65 years	1107	NA	57.3
Klein 2020 [19]	Cell‐based‐3v (vs. SD‐3/4v)	Retro‐spective cohort	USA	Non‐industry funded	Outpatient	Influenza infection	2017–18	4–64 years	1,016,965	NA	Cell‐based 56.9 SD 56.4
Martin 2021 [20]	Cell‐based‐3v (vs. SD‐3/4v)	Retro‐spective cohort	USA	Non‐industry funded	Inpatient	Influenza‐related hospitalisation	2017–18	≥ 18 years	2350	NA	NA
Zimmerman 2023 [21]	Recombinant‐ 4v (vs. SD‐3/4v)	Test‐negative	USA	Industry funded	Outpatient	Medically attended outpatient influenza	2018–19, 2019–20	≥ 18 years, high‐risk condition, immuno‐compromised	1553	51.5 (18.8)	65.6
Hsiao 2022 [22]	Recombinant‐ 4v (vs. SD‐4v)	RCT	USA	Industry funded	Inpatient	Influenza‐related hospitalisation	2018–19, 2019–20	≥ 18–64 years	1,630,328	NA	NA
Domnich 2022 [23]	MF59‐3v (vs. SD‐4v)	Test‐negative	Italy	Non‐industry funded	Inpatient	Influenza‐related hospitalisation	2018–19, 2019–20	≥ 65 years	512	Cases 78.9 (7.5)	Cases 50.6 controls 41.0
										Controls 79.6 (7.6)	

Abbreviations: HD = high‐dose influenza vaccine; NA = not applicable; SD = standard influenza vaccine (egg‐based standard‐dose influenza vaccine containing 15 μg HA); 3v/4v = tri‐/quadrivalent.

In the primary review [4], there were a total of 10 studies identified that provided estimates of rVE against laboratory‐confirmed outcomes, compared to standard vaccine. For details on these studies, see Table 2 and the primary review [4]. Seven of these studies reported rVE data on the MF59‐adjuvanted vaccine, all of which were rVE estimates against laboratory‐confirmed influenza. One study reported rVE of the high‐dose vaccine against laboratory‐confirmed influenza. One study reported rVE of the cell‐based vaccine against laboratory‐confirmed influenza. Another study reported this outcome for the recombinant vaccine. We did not identify rVE estimates for the other efficacy/effectiveness outcomes.

**TABLE 2:** Key characteristic of relative vaccine efficacy/effectiveness studies of primary review, included in the evidence body of the review update (10 studies).

Study	Intervention (comparison)	Study design	Country	Setting	Outcome	Influenza season	Population	*N* (vacc)
Van Buynder 2013 [24]	MF59‐3v (vs. SD‐3v)	Case‐control	Canada	Multicentre	Laboratory‐confirmed influenza	2011–2012	Adults aged ≥ 65 years	282
Mira‐Iglesias 2019 [25]	MF59‐3v (vs. SD‐3v)	Case‐control	Spain	Hospital	Laboratory‐confirmed influenza	2017–2018	Adults aged ≥ 60 years	1477
Pebody 2020a [26]	MF59‐3v (vs. SD‐3v/4v)	Case‐control	United Kingdom	General practice and hospitals	Laboratory‐confirmed influenza hospitalisation	2018–2019	Adults aged ≥ 65 years	1439
Pebody 2020b [27]	MF59‐3v (vs. SD‐3v/4v)	Case‐control	United Kingdom	General practice	Laboratory‐confirmed influenza	2018–2019	Children and adults aged > 0 years	2326
Bellino 2019 [28]	MF59‐3v (versus SD‐3v/4)	Case‐control	Italy	General practice	Laboratory‐confirmed influenza	2018–2019	Children and adults aged ≥ 6 months	2526
Rondy 2017a [29]	MF59‐3v (vs. SD‐3v)	Case‐control	Europe	Multicentre, hospital	Laboratory‐confirmed influenza	2016–2017	Adults aged ≥ 65 years	640
Rondy 2017b [30]	MF59‐3v (vs. SD‐3v)	Case‐control	Europe	Multicentre, hospital	Laboratory‐confirmed influenza	2015–2016	Adults aged ≥ 65 years	1802
DiazGranados 2014 [31]	HD‐3v (vs. SD‐3v)	RCT	United States and Canada	Multicentre	Laboratory‐confirmed ILI	2011–2013	Adults aged ≥ 65 years	31,989
Bruxvoort 2019 [32]	Cell‐based‐3/4 versus SD‐v3/4	Case control	United States	Hospital	Laboratory‐confirmed influenza hospitalisation	2017–2018	Children and adults (aged ≥ 4 years)	8132
Dunkle 2017a [33]	Recombinant‐ 4v versus SD‐4v	RCT	United States	Multicentre	Culture‐confirmed influenza‐like illness, PCR‐confirmed ILI	2014–2015	Adults (aged ≥ 50 years)	9003
				Outpatients				

Abbreviations: HD = high‐dose influenza vaccine; NA = not applicable; SD = standard influenza vaccine (egg‐based standard‐dose influenza vaccine containing 15 μg HA); 3v/4v = tri‐/quadrivalent.

*Safety studies:* Details of studies that reported safety data of newer/enhanced influenza vaccines (as compared to standard influenza vaccines) and were found in the update search are provided in Table 3. We identified five RCTs. In addition, five NRSI (both retrospective cohort studies) were identified. The studies were performed in Australia, Belgium, Germany, Japan, France, Italy, the Netherlands, Poland, Taiwan and the USA and had about 40 to > 1 million participants. Five studies investigated the high‐dose influenza vaccine. Two studies provided data for the recombinant and two others for the MF59‐adjuvanted vaccine. No study reported on a mRNA‐based influenza vaccine. Nine studies reported on serious adverse events. For systemic reactions, six studies gave data on fever and four studies reported on headache. Regarding local reactions, six studies reported data on pain at the injection site and three on swelling.

**TABLE 3:** Key characteristics of vaccine safety studies included in the review update based on update search (10 studies).

Study	Intervention (comparison)	Study design	Country	Type of funding	Population	*N* vaccinated	Mean age in years (SD)	Female sex ‐ %	Safety outcomes available
Caldera 2020 [34]	HD‐3v (vs. SD‐4v)	RCT	USA	Non‐industry funded	Patients with inflammatory bowel disease on anti‐tumour necrosis factor alpha agents 18–64 years	40	Median (IQR) HD 29 (25–45) SD 43 (32–52)	HD 36 SD 33	Local and systemic reactions
Chen 2022 [35]	HD‐4v (vs. SD‐4v)	RCT	Taiwan	Industry‐funded	≥ 65 years	165	71.4 (5.52)	HD 57.3 SD 55.4	Local and systemic reactions SAE
Layton 2020 [36]	HD‐3v (vs. SD)	Retrospective cohort	USA	Not reported	≥ 65 years with end‐stage renal disease	520,876	74.7 (7.0)	49.5	Local and systemic reactions SAE
Pepin 2021 [37]	HD‐4v (vs. SD‐4v)	RCT	Belgium, France, Germany, Italy, Poland, The Netherlands	Industry‐funded	≥ 60 years	1533	66.6 (5.97)	50.4	Unsolicited non‐serious injection‐site AE Unsolicited non‐serious systemic AE, SAE, AESI
Sanchez 2023 [38]	HD‐4v (vs. SD‐4v)	RCT	Japan	Industry‐funded	> 60 years	2100	HD 68.2 (4.9) SD 68.4 (5.0)	HD 46.3 SD 47.9	Local and systemic reactions SAE
Pillsbury 2020 [39]	HD‐3v (vs. MF59)	Retrospective cohort	Australia	Non‐industry funded	≥ 65 years	47,307	Median (IQR) 71 (68–76)	54.0	Local and systemic reactions SAE
Schmader 2021 [40]	MF59 (vs. HD)	RCT	USA	Non‐industry funded	≥ 65 years	757	Median age (range) 72 (65–97)	55.0	Local and systemic reactions SAE
de Lusignan 2022 [41]	MF59 (vs. SD‐4v)	Retrospective cohort	UK	Non‐industry funded	0–100 years	1,024,160	NA	NA	Local and systemic reactions SAE
Hansen 2020 [42]	Recombinant‐3v (vs. SD‐3v)	Retrospective cohort	USA	Industry‐funded	≥ 18 years, pregnant women included	305,659	NA	Rec 52.7 SD 55.3	SAE Fever
Hsiao 2022 [43]	Recombinant‐4v (vs. SD‐4v)	Prospective cohort	USA	Industry‐funded	Chinese adults 18–64 years, pregnant women included	42,684	18–65 years	63.8	SAEs Fever

Abbreviations: AE = adverse event; AESI = adverse event; HD = high‐dose; influenza vaccine; NA = not applicable; SAE = serious adverse event; SD = standard influenza vaccine (egg‐based standard‐dose influenza vaccine containing 15 μg HA); 3v/4v = tri‐/quadrivalent.

In the primary review, there were 32 studies identified which reported data on the above‐mentioned safety outcomes, as compared to standard vaccine [4]. Study characteristics are reported in Table 4 and in the primary review [4]. Twelve of these studies reported safety data on the MF59‐adjuvanted vaccine and 7 studies had estimates for high‐dose vaccine. For the cell‐based vaccine, 6 studies reported safety estimates, while 7 studies were available for the recombinant vaccine.

**TABLE 4:** Key characteristics of vaccine safety studies of primary review, included in the evidence body of the review update (32 studies).

Study	Intervention (comparison)	Study design	Country	Population	*N* vaccinated	Safety outcomes available
Cowling 2020 [44]	MF59‐3v (vs. SD‐4v)	RCT	Hong Kong	Community dwelling Adults aged 65–82 years	1861	Local adverse events, systemic adverse events, serious adverse events
Cowling 2020 [44]	Recombinant‐3v (vs. SD‐4v)	RCT	Hong Kong	Community dwelling Adults aged 65–82 years	1861	Serious adverse events, hospitalisation
de Bruijn 2006 [45]	MF59‐3v (Subunit influenza vaccine)	RCT	Netherlands	Adults aged ≥ 61 years	386	Mortality, serious adverse events, local adverse events, systemic adverse events
Durando 2008 [46]	MF59‐3v (vs. SD‐3v)	RCT	Italy	Healthy adults aged ≥ 65 years	270	Serious adverse events, any adverse event
Frey 2003 [47]	MF59‐3v (vs. SD‐3v)	RCT	United States	Adults aged 18–64 years	301	Local adverse events, systemic adverse events
Frey 2003 [47]	MF59‐3v (vs. SD‐3v)	RCT	United States, Philippines, Panama and Columbia	Adults aged ≥ 65 years	7109	Mortality, local adverse events systemic adverse events
Gasparini 2001 [48]	MF59‐3v (vs. SD‐3v)	RCT	Italy	Adults aged 18–65 years, HIV seropositive	308	Serious adverse events, local adverse events, systemic adverse events
Li 2008 [49]	MF59‐3v (vs. SD‐3v)	RCT	China	Adults aged ≥ 60 years	600	Serious adverse events, local adverse events, systemic adverse events
Minutello 1999 [50]	MF59‐3v (vs. SD‐3v)	RCT	Italy	Adults aged ≥ 65 years	92	Serious adverse events, local adverse events, systemic adverse events
Ruf 2004 [51]	MF59‐3v (vs. SD‐3v)	RCT	Germany	Adults aged ≥ 60 years	827	Local and general symptoms, serious adverse events
Scheifele 2013 [52]	MF59‐3v (vs. SD‐3v)	RCT	Canada	Adults aged ≥ 65 years	922	Serious adverse events, mortality, local adverse events, systemic adverse events
Seo 2014 [53]	MF59‐3v (vs. SD‐3v)	RCT	South Korea	Healthy, independently‐living adults aged ≥ 65 years	354	Local adverse events, systemic adverse events
Sindoni 2009 [54]	MF59‐3v (vs. SD‐3v)	RCT	Italy	Adults (aged ≥ 65 years)	195	Serious adverse events, local adverse events, systemic adverse events
Couch 2007 [55]	HD‐v3 (vs. SD‐3v)	RCT	United States	Adults aged ≥ 65 years	414	Serious adverse events, mortality, local adverse events, systemic adverse events
DiazGranados 2015b [56]	HD‐v3 (vs. SD‐3v)	RCT	United States	Adults aged 50–64 years	300	Serious adverse events, mortality, local adverse events, systemic adverse events
Falsey 2009 [57]	HD‐v3 (vs. SD‐3v)	RCT	United States	Adults aged ≥ 65 years	3876	Mortality, local adverse events, systemic adverse events
Keitel 2006 [58]	HD‐v3 (vs. SD‐3v)	RCT	United States	Adults aged ≥ 65 years	202	Serious adverse events, mortality, local adverse events, systemic adverse events
Tsang 2014 [59]	HD‐v3 (vs. SD‐3v)	RCT	United States	Adults aged ≥ 65 years	1912	Serious adverse events, mortality, local adverse events, systemic adverse events
Noh 2019 [60]	HD‐4v (vs. SD‐4v)	RCT	Republic of Korea	Adults aged 19–64 years	40	Local adverse events, systemic adverse events
Pillet 2019 [61]	HD‐4v (vs. SD‐4v)	RCT	United States	Adults aged ≥ 18 years	750	Serious adverse events, local adverse events, systemic adverse events
Ehrlich 2012 [62]	Cell‐based‐3v (vs. SD‐3v)	RCT	United States	Adults aged > 50 years	3208	Serious adverse events, mortality, local adverse events, systemic adverse events
Frey 2010 [63]	Cell‐based‐3v (vs. SD‐3v)	RCT	United States, Poland and France	Healthy adults aged 18–49 years	11,404	Serious adverse events, local adverse events, systemic adverse events
Groth 2009 [64]	Cell‐based‐3v (vs. SD‐3v)	RCT	Germany	Adults aged ≥ 18 years	240	Serious adverse events, mortality, local adverse events, systemic adverse events
Halperin 2002 [65]	Cell‐based‐3v (vs. SD‐3v)	RCT	Canada	Adults and children aged ≥ 3 years	940	Local adverse events, systemic adverse events
Song 2015 [66]	Cell‐based‐3v (vs. SD‐3v)	RCT	Republic of Korea	Adults aged ≥ 19 years	1155	Serious adverse events, local adverse events, systemic adverse events
Szymczakiewicz‐Multanowska 2009 [67]	Cell‐based‐3v (vs. SD‐3v)	RCT	Poland	Adults aged ≥ 18 years	2654	Serious adverse events, mortality, local adverse events, systemic adverse events
Dunkle 2017a [68]	Recombinant‐3v (vs. SD‐4v)	RCT	United States	Adults aged ≥ 50 years	9003	Serious adverse events, mortality, local adverse events, systemic adverse events
Dunkle 2017b [33]	Recombinant‐3v (vs. SD‐4v)	RCT	United States	Adults aged 15–49 years	1350	Serious adverse events, mortality, local adverse events, systemic adverse events
Baxter 2011 [69]	Recombinant‐3v (vs. SD‐3v)	RCT	United States	Healthy adults aged 50–64 years	602	Serious adverse events, mortality, local adverse events, systemic adverse events
Izikson 2015 [70]	Recombinant‐3v (vs. SD‐3v)	RCT	United States	Adults aged ≥ 50 years	2640	Serious adverse events, mortality, local adverse events, systemic adverse events
Keitel 2009 [71]	Recombinant‐3v (vs. SD‐3v)	RCT	United States	Adults aged ≥ 65 years	869	Serious adverse events, mortality, local adverse events, systemic adverse events
Treanor 2006 [72]	Recombinant‐3v (vs. SD‐3v)	RCT	United States	Adults aged ≥ 18 years	399	Local adverse events, systemic adverse events

Abbreviations: AE = adverse event; AESI = adverse event; HD = high‐dose; influenza vaccine; NA = not applicable; SAE = serious adverse event; SD = standard influenza vaccine (egg‐based standard‐dose influenza vaccine containing 15 μg HA); 3v/4v = tri‐/quadrivalent.

In the following, only data on serious adverse events are presented. Local and systemic reactions are summarised in the Appendix (Supporting Information S1: Appendix E).

### 3.2 Risk of Bias

Across the 6 NRSI that were identified in the update search and reported data on VE outcomes, overall risk of bias was moderate for each outcome and study, respectively. Main reason for this assessment was that residual confounding (domain 1) could not be excluded in all studies (see Supporting Information S1: Appendix C for details). Risk of bias could not be assessed for the cluster‐RCT [22] since data were only presented in a conference abstract with limited information.

The overall risk of bias was moderate to critical in the 5 NRSI studies that were identified in the update search and reported data on safety outcomes (see Supporting Information S1: Appendix E). Main reason for this assessment was that residual confounding (domain 1) could not be excluded in all studies. For two studies confounding was assessed critical, since only unadjusted data were reported (see Supporting Information S1: Appendix C). Risk of bias assessments for all safety outcomes (SAE, pain, swelling, headache, fever) can be found in Supporting Information S1: Appendix C. No evidence for publication bias was found (see Supporting Information S1: Appendix D).

### 3.3 MF59‐Adjuvanted Influenza Vaccine

*Efficacy/effectiveness against laboratory‐confirmed influenza:* In the primary review, seven studies (all NRSI) were included that reported a total of 13 estimates while no additional study was identified in the update (Table 5). Relative VE estimates were highly heterogenous and ranged from −30% to 88%, with only two estimates being statistically significant. Due to heterogeneity, metanalysis was not performed.

**TABLE 5:** Relative effectiveness of MF59‐adjuvanted influenza vaccine versus standard influenza vaccine, for laboratory confirmed influenza.

Study	Study design	rVE	95% CI	Season
All strains				
Van Buynder 2013	NRSI	42%	−8% to 69%	2011–2012
Mira‐Iglesias 2019	NRSI	19%	−10% to 41%	2017–2018
Pebody 2020a	NRSI	30%	−83% to 73%	2018–2019
Pebody 2020b	NRSI	16%	−176% to 75%	2018–2019
Bellino 2019a	NRSI	−1%	−122% to 59%	2018–2019
A (H1N1)				
Mira‐Iglesias 2019	NRSI	−3%	−126% to 53%	2017–2018
Pebody 2020a	NRSI	3%	−358% to 79%	2018–2019
A (H3N2)				
Rondy 2017b	NRSI	88%	51% to 100%	2015–2016
Rondy 2017a	NRSI	−30%	−146% to 31%	2016–2017
Mira‐Iglesias 2019	NRSI	20%	−17% to 46%	2017–2018
Pebody 2020a	NRSI	43%	−134% to 86%	2018–2019
B				
Rondy 2017b	NRSI	87%	30% to 100%	2015–2016
Mira‐Iglesias 2019	NRSI	6%	−58% to 44%	2017–2018

Abbreviations: CI = confidence interval; NRSI = non‐randomised studies; rVE = relative vaccine efficacy/effectiveness.

*Relative Efficacy/effectiveness against influenza‐related hospitalisation:* No studies matching the inclusion criteria of this update were identified in the primary review.

In the update, we identified one NRSI [23]. The authors reported rVE against hospitalisation due to influenza (laboratory‐confirmed) from two consecutive seasons (2018–2020). Relative VE against all strains was 59.2% (95%CI: 14.6% to 80.5%). For influenza A, VE was 63.7% (95%CI: 22.8% to 82.9%).

*Relative Efficacy/effectiveness against other outcomes:* No studies reported on influenza‐related death, influenza‐related ICU admission or influenza‐associated cardiovascular disease, neither in the primary review nor in the update. For influenza‐like illness, one study was reported in the primary review [73] which used a case definition not covered by the protocol of this review.

*Serious adverse events:* In the primary review, 3 RCTs and 2 NRSI were identified to report serious adverse events (SAE). In the RCTs [49, 74, 75], a total of 3 SAE were identified in the MF59‐adjuvanted vaccine group, including 2 cases of Guillain‐Barré‐Syndrome, and 3 SAE were found in the standard vaccine group. The NRSIs reported no cases of narcolepsy in both study groups (MF59‐adjuvanted vaccine and standard vaccine) [76] and no group difference in hospitalised SAE [77]. In the update, no additional studies were identified. The pooled relative risk of SAE after vaccination with MF59‐adjuvanted influenza vaccine as compared to standard influenza vaccine was 0.95 (95%CI: 0.19 to 4.72; fixed‐effects model).

### 3.4 High‐Dose Influenza Vaccine

*Relative efficacy/effectiveness against laboratory‐confirmed influenza:* In the primary review, one RCT was included that reported a rVE of 24.2% (95%CI: 9.7% to 36.5%) against laboratory‐confirmed influenza (all strains) during two consecutive seasons (2011–2013) [31]. In the update, we identified one NRSI [17] which reported rVE estimates against influenza A during four consecutive seasons (2015–2019). Relative VE ranged between −9% and 19%, with none of the estimates being statistically significant (see Table 6).

**TABLE 6:** Relative vaccine effectiveness of high‐dose versus standard influenza vaccine against laboratory‐confirmed influenza and influenza‐related hospitalisation (laboratory‐confirmed).

Study	Study design	rVE	95% CI	Season
Laboratory confirmed influenza				
All strains				
Diaz‐Granados 2014	RCT	24.2%	9.7% to 36.5%	2011–2013
A				
Balasubramani 2020	NRSI	10%	−15% to 30%	2015–2019
Balasubramani 2020	NRSI	−9%	−158% to 54%	2015–2016
Balasubramani 2020	NRSI	2%	−69% to 43%	2016–2017
Balasubramani 2020	NRSI	6%	−55% to 43%	2017–2018
Balasubramani 2020	NRSI	19%	−27% to 48%	2018–2019
Influenza‐related hospitalisation (lab‐confirmed)				
All strains				
Doyle 2020	NRSI	27%	−1 to 48%	2015–2017
Doyle 2020	NRSI	24%	−46% to 61%	2015–2016
Doyle 2020	NRSI	27%	−8 to 50%	2016–2017
A				
Doyle 2020	NRSI	22%	−15% to 46%	2015–2017
B				
Doyle 2020	NRSI	44%	−13% to 73%	2015–2017

Abbreviations: CI = confidence interval; NRSI = non‐randomised studies; rVE = relative vaccine efficacy/effectiveness.

*Relative efficacy/effectiveness against influenza‐related hospitalisation:* No studies matching the inclusion criteria of this update were identified in the primary review. In the update, we identified one NRSI [18]. Relative VE against hospitalisation due to influenza (laboratory‐confirmed) was reported for two consecutive seasons, against influenza A, B and all strains separately. Relative VE against all strains was 27% (95%CI: −1 to 48). None of the rVE estimates was statistically significant (see Table 6).

*Relative efficacy/effectiveness against other outcomes:* No studies reported on influenza‐related death, influenza related ICU admissions or influenza‐associated cardiovascular disease, neither in the primary review nor in the update. For influenza‐like illness, one study was reported in the primary review [78] which used a case definition derived from claims data not covered by the protocol of this review.

*Serious adverse events:* In the primary review, 6 SAEs, incl. neuropathy, cranial nerve VI palsy, shock, Crohn's disease, myasthenia gravis and encephalomyelitis were reported in 3RCTs after high‐dose vaccine administration [56, 57, 79]. One NRSI reported no increased risk of Guillain‐Barré syndrome in the primary analysis [80]. In the update, we identified 3 RCTs and 1 NRSI which reported data on SAE. Two of the RCTs (Chen 2022, Sanchez 2023) did not observe SAEs in their study groups. One RCT [37] reported 5 SAEs (60–64 years: 1; ≥ 65 years: 4) in the high‐dose vaccine group and 7 SAEs (60–64 years: 2; ≥ 65 years: 5) in the standard vaccine group. One NRSI [36] did not find an increased risk of seizure (RR: 1.03 [95% CI: 0.81 to 1.32]), encephalopathy (RR: 0.94 [95% CI: 0.78 to 1.14]) or short‐term death (RR: 1.09 [95% CI: 0.8 to 1.48]) after high‐dose vaccine, as compared to standard vaccine. The pooled relative risk of SAE after vaccination with high‐dose influenza vaccine as compared to standard influenza vaccine was 1.02 (95%CI: 0.42 to 2.46; fixed‐effects model).

### 3.5 Cell‐Based Influenza Vaccine

*Relative efficacy/effectiveness against laboratory‐confirmed influenza:* In the primary review, one NRSI [32] was included that reported rVE against laboratory‐confirmed influenza (all strains and A/H3N2) during two seasons (2014–2015 and 2017–2018). In the update, we identified one additional NRSI [19], which reported rVE estimates against influenza A and B during one season (2017–2018). Relative VE in these two studies ranged between −5.8% and 21.4%, with none of the estimates being statistically significant (see Table 7 for details).

**TABLE 7:** Relative vaccine effectiveness of cell‐based versus standard influenza vaccine influenza vaccine against laboratory‐confirmed influenza and influenza‐related hospitalisation (laboratory‐confirmed).

Study	Study design	rVE	95% CI	Season
Laboratory confirmed influenza				
All strains				
Bruxvoort 2019	NRSI	6%	−46% to 39%	2014–2015
A (H3N2)				
Bruxvoort 2019	NRSI	4%	−70% to 37%	2014–2015
A				
Klein 2020	NRSI	−5.8%	−36.1% to 17.7%	2017–2018
B				
Klein 2020	NRSI	21.4%	−7.3% to 42.4%	2017–2018
Influenza‐related hospitalisation (lab‐confirmed)				
All strains				
Martin 2021	NRSI	8.5%	−75.9% to 52.3%	2017–2018
A				
Martin 2021	NRSI	24.9%	−78.8% to 68.5%	2017–2018
B				
Martin 2021	NRSI	1.8%	−254% to 72.8%	2017–2018

Abbreviations: CI = confidence interval; NRSI = non‐randomised studies; rVE = relative vaccine efficacy/effectiveness.

*Relative efficacy/effectiveness against influenza‐related hospitalisation:* No studies matching the inclusion criteria of this update were identified in the primary review. In the update, we identified one NRSI [20]. Relative VE against hospitalisation due to influenza (laboratory‐confirmed) was reported for one season (2017–2018), against influenza A and B separately. None of the rVE estimates ranging between 1.8% and 24.9% was statistically significant (see Table 7).

*Relative efficacy/effectiveness against other outcomes:* No studies reported on the other predefined outcomes, neither in the primary review nor in the update.

*Serious adverse events:* In the primary review, 1 SAE (hypersensitivity) was reported in 1 RCT after cell‐based vaccine administration [62]. The relative risk of SAE after vaccination with cell‐based influenza vaccine as compared to standard influenza vaccine was 0.39 (95%CI: 0.02 to 9.49; fixed‐effects model). No additional data were identified in the update.

### 3.6 Recombinant Influenza Vaccine

*Relative efficacy/effectiveness against laboratory‐confirmed influenza:* In the primary review, one RCT was included that reported rVE estimates from one season (2014–2015) for all strains and influenza A and B separately [33]. Relative VE against all strains was 30% (95%CI: 10% to 47%), while it was 36% (95%CI: 14% to 53%) against influenza A and 4% (95%CI: −42% to 56%) against influenza B. In the update, we identified one NRSI [21] which reported rVE estimates (all strains) during two consecutive seasons (2018–2019). Relative VE ranged between −3% and 6%, with none of the estimates being statistically significant (Table 8 for details).

**TABLE 8:** Relative vaccine effectiveness of recombinant versus standard influenza vaccine against laboratory‐confirmed influenza and influenza‐related hospitalisation (laboratory‐confirmed).

Study	Study design	rVE	95% CI	Season
Laboratory confirmed influenza				
All strains				
Dunkle 2017	RCT	30%	10% to 47%	2014–2015
Zimmerman 2023	NRSI	3%	−31% to 28%	2018–2020
Zimmerman 2023	NRSI	6%	−48% to 40%	2018–2019
Zimmerman 2023	NRSI	−3%	−52% to 30%	2019–2020
A				
Dunkle 2017	RCT	36%	14% to 53%	2014–2015
B				
Dunkle 2017	RCT	4%	−42% to 56%	2014–2015
Influenza‐related hospitalisation (lab‐confirmed)				
Age 18–49 years				
Hsiao 2022	RCT	−7.3%	−52.1% to 24.4%	2018–2020
Age 50–64 years				
Hsiao 2022	RCT	16.3%	−8.7% to 35.5%	2018–2020

Abbreviations: CI = confidence interval; NRSI = non‐randomised studies; rVE = relative vaccine efficacy/effectiveness.

*Relative efficacy/effectiveness against influenza‐related hospitalisation:* No studies matching the inclusion criteria of this update were identified in the primary review. In the update, we identified one cluster‐RCT [22] which reported rVE data for two separate age groups obtained during two consecutive seasons (2018–2020). VE was −7.3% (95%CI: −52.1% to 24.4%) for the age group 18–49 years and 16.3% (95%CI: −8.7% to 35.5%) for the age group 50–64 years (Table 8).

*Relative efficacy/effectiveness against other outcomes:* No studies reported on the other predefined outcomes, neither in the primary review nor in the update.

*Serious adverse events:* In the primary review, 2 RCTs reported 2 SAE (syncope; pericardial effusion) after administration of the recombinant vaccine [69, 81]. The pooled relative risk of SAE after vaccination with recombinant influenza vaccine as compared to standard influenza vaccine was 3.04 (95%CI: 0.32 to 29.10; fixed‐effects model). In the update, 2 NRSI were identified which reported on various SAE. One NRSI [43] reported no significantly increased risk of death (OR 0.49 [95%CI: 0.21 to 1.05]), idiopathic thrombocytopenic purpura (OR 0.90 [95%CI: 0.03 to 11.81]), non‐infectious pleural effusion (OR 1.76 [95%CI: 0.05 to 68.70]) and convulsion (OR 0.90 [95%CI: 0.03 to 11.81]) after recombinant vaccine, compared to standard vaccine. The other NRSI [42] found no increased risk of Guillain‐Barré syndrome in inpatient or emergency department settings (OR 0 [95%CI: 0 to 16.07]) or in outpatients (OR 0 [95%CI: 0 to 112.6]). Furthermore, they did not detect an increased risk of non‐infectious pleural effusion (OR 0 [95%CI: 0 to 4.8]) or narcolepsy/cataplexy (OR 0 [95%CI: 0 to 6]).

### 3.7 mRNA‐Based Influenza Vaccine

No studies reporting efficacy/effectiveness or safety outcomes after vaccination with mRNA‐based influenza vaccines were identified.

### 3.8 GRADE Certainty of Evidence (CoE)

For the MF59‐adjuvanted vaccine, CoE was assessed as being low for the outcome laboratory‐confirmed influenza and moderate for influenza‐related hospitalisation (1 NRSI, downgraded by 1 for risk of bias). For serious adverse events, CoE was low.

For the high‐dose vaccine, CoE was moderate for the outcome laboratory‐confirmed influenza (1 RCT; downgraded by 1 due to risk of bias). Certainty was assessed to be low for influenza‐related hospitalisation. For serious adverse events, CoE was low.

For the cell‐based vaccine, CoE was low for the outcome laboratory‐confirmed influenza (2 NRSI, downgraded by 2 due to risk of bias and inconsistency). Certainty was low for influenza‐related hospitalisation (1 NRSI; downgraded by 1 for risk of bias and 1 for imprecision). For serious adverse events, CoE was low.

For the recombinant vaccine, CoE was assessed to be moderate for laboratory‐confirmed influenza (1 RCT; downgraded by 1 due to risk of bias). For serious adverse events, CoE was low.

Due to lack of data, no certainty of the evidence assessment was possible for the mRNA‐based vaccine (see Supporting Information S1: Appendix E for all summary of findings tables).

## 4 Discussion

The aim of this update of an existing systematic review [4] was to reassess the evidence on the efficacy/effectiveness and safety of newer and enhanced influenza vaccines by updating the search, but also by narrowing the focus of the research question to relative efficacy/effectiveness (i.e. comparison with standard influenza vaccines) and allowing for new technologies (mRNA‐based vaccines). In addition, we intended to overcome some methodological limitations of the primary review. In particular, we no longer included effectiveness outcomes which had not been laboratory‐confirmed, with the exception of influenza‐like illness (ILI) where we included studies that used internationally accepted outcome definitions (e.g., by WHO or US‐CDC). Main reason for this decision was that non‐randomised studies (observational studies) which do not use laboratory‐confirmed outcomes to assess influenza vaccine effectiveness have been shown to be prone to healthy vaccinee bias as well as confounding by indication [82]. Moreover, it has been demonstrated that these forms of bias cannot be eliminated by statistical procedures to control for confounding [82]. Consequently, studies using non‐laboratory confirmed ICD‐codes or claims data (or compound outcomes derived from such data) which were included in the primary review [83, 84, 85, 86, 87, 88, 89, 90, 91, 92, 93, 94] were not used in this update.

While new data accumulated since 2020 were reassuring regarding the safety of the vaccines, the evidence base regarding the relative efficacy/effectiveness of these vaccines against laboratory‐confirmed outcomes has only slightly improved. For two of the new vaccines, that is high‐dose vaccine and recombinant vaccine, findings from recent NRSI even contradicted previous findings from RCTs regarding rVE against laboratory‐confirmed influenza. For the high‐dose vaccine, an RCT [31] reported a rVE (compared to standard vaccine) of more than 20%, whereas a recent test‐negative study [17] with moderate risk of bias did not observe a statistically significant rVE in any of the four consecutive influenza seasons investigated. Likewise, for the recombinant vaccine, an RCT [33] found a rVE of 30%, whereas a recent test‐negative study [21] (moderate risk of bias) did not find any effect during two consecutive seasons. While the GRADE certainty of evidence assessments was still based on the RCT data, the review of the evidence summarised might contribute as one of the elements for decision making.

However, there was one exception where the evidence base on VE has substantially improved. A test‐negative study [23] provided, for the first time, rVE estimates against laboratory‐confirmed influenza‐related hospitalisation for the MF59‐adjuvanted vaccine.

After the date of the last search of this update, a study on the rVE of recombinant influenza vaccine versus standard‐dose influenza vaccine was published [95]. This cluster RCT described a rVE of 15.3% (95%CI: 5.9 to 23.8) against laboratory‐confirmed influenza. Some data on the rVE against influenza‐related hospitalisation presented in this RCT were already published as a congress abstract and included in this update [22].

The new studies identified in this update which investigated rVE were all assessed to have moderate risk of bias. Compared to the primary review where a substantial number of included NRSI had serious risk of bias, we observed a considerable increase in overall study quality. However, there is still a lack of data regarding a number of laboratory‐confirmed outcomes for all vaccines investigated in this review update, including evidence gaps regarding safety of administering these vaccines during pregnancy. Even these studies, which reported to have included pregnant women [42, 43], did not present data separately for this subgroup information to be included in our review. Lastly, so far, no data on rVE or safety of the mRNA‐vaccines are available.

Regarding potential biases in the study identification process, there is a small chance that our search might have missed potentially relevant studies. However, this appears unlikely since the search string was built upon the successful strategy of the primary review and has been assessed by an experienced information specialist. In the screening process, there remains a small possibility that we have overlooked laboratory‐confirmed outcomes in studies which were therefore excluded. We think, however, that this is unlikely since the review process was conducted by pairs of experienced reviewers and a senior reviewer was involved in every case of uncertainty. Finally, risk of bias assessment is always subjective to some extent and therefore, other reviewers might have come to different assessment results. We tried to minimise subjectivity by conducting independent assessments by a pair of reviewers and allowing for an in‐depth team discussion of the results of the risk of bias judgements.

We are aware of another systematic review published in 2021 (data cut: 15 July 2020) which analysed the MF59‐adjuvanted vaccine [96]. This review came to more favourable conclusions regarding the relative effectiveness of this vaccine. However, the authors included non‐laboratory‐confirmed outcomes as well. Of note, the review was co‐authored by representatives of the manufacturer which constitutes a conflict of interest. A recently published systematic review and network‐meta‐analysis [97] concluded that the recombinant vaccine has the highest VE of all newer and enhanced influenza vaccines. However, this review had different inclusion criteria (e.g., used all‐cause mortality as an outcome) and focussed on comparison to placebo.

## 5 Conclusions

This update of a systematic review shows that the evidence on relative efficacy/effectiveness of newer and/or enhanced influenza vaccines, compared to standard influenza vaccines, is still limited and partially heterogeneous.

Low to moderate relative vaccine effectiveness has been shown for the MF59‐adjuvanted vaccine, the high‐dose and the recombinant vaccine for laboratory confirmed influenza. Low‐to‐moderate relative vaccine effectiveness was also found for the MF59‐adjuvanted vaccine and the high‐dose vaccine for laboratory‐confirmed influenza‐related hospitalisation. A larger evidence base is available on safety, demonstrating an overall favourable safety profile for all vaccines. Further studies are needed, preferably using randomised designs and particularly regarding laboratory‐confirmed outcomes and safety data including during pregnancy to allow for more substantial conclusions on the potential benefits of these vaccines. As several countries have implemented policies in recent years to consider all enhanced influenza vaccines equivalent for the purposes of vaccine programs (e.g. USA, UK, Canada), it remains important to continue following this evidence landscape, since it could have significant implications for global vaccine programs if one product is continuously outperforming others.

## Author Contributions

All authors attest they meet the ICMJE criteria for authorship. Mona Askar, Madeleine Batke, Timo Brugger, Annika Falman, Wiebe Külper‐Schiek, Joerg J Meerpohl, Vanessa Piechotta, Johanna Schlaberg, Christine Schmucker, Waldemar Siemens, Jan Stratil, Thomas Harder: Writing original draft, reviewing, editing, investigation, formal analysis. Karam Adel, Anna Hayman Robertson, Jaime Jesús Pérez, Kari Johansen, Jorgen de Jonge, Tyra Grove Krause, Angeliki Melidou, Hanna Nohynek, Carmen Olmedo, Kate Olsson, Ioanna Pavlopoulou, Johanna Rubin, Veronika Učakar, Ole Wichmann: Idea conception, protocol development, reviewing, editing, expert input.

## Ethics Statement

The authors have nothing to report.

## Conflicts of Interest

The authors declare no conflicts of interest.

## Supporting information

Supporting Information S1

## Data Availability

The data that supports the findings of this study are available in the supplementary material of this article.
