# Supplementary material for: Relative Efficacy, Effectiveness and Safety of Newer and/or Enhanced Seasonal Influenza Vaccines for the Prevention of Laboratory‐Confirmed Influenza in Individuals Aged 18 years and Over: Update of a Systematic Review
Source: Rev Med Virol. 2025 Feb 24;35(2):e70020. doi: 10.1002/rmv.70020 (PMC11850296; doi:10.1002/rmv.70020)
Supplement: Supplementary file 1 — Supporting Information S1 [file RMV-35-e70020-s001.docx]

**Supplementary material**

**Relative efficacy, effectiveness and safety of newer and/or enhanced seasonal influenza vaccines for the prevention of laboratory-confirmed influenza in individuals aged 18 years and over: Update of a systematic review**

# Appendix A – Search Strategies

**Medline [Ovid]**

Date run: 24.07.2023

| **#** | **Searches** | **Results** |
| --- | --- | --- |
| 1 | Influenza, Human/ | 57851 |
| 2 | Influenza A virus/ or exp Influenza B virus/ | 25030 |
| 3 | influenza.ti. | 79308 |
| 4 | 1 or 2 or 3 | 97222 |
| 5 | vaccines/ or vaccines, attenuated/ or vaccines, inactivated/ or vaccines, subunit/ or vaccines, synthetic/ or nucleic acid-based vaccines/ or mrna vaccines/ or vaccines, conjugate/ or vaccines, virosome/ or vaccines, virus-like particle/ or vaccines, live, unattenuated/ or viral vaccines/ or influenza vaccines/ | 112523 |
| 6 | exp Vaccination/ or Immunization/ or Immunotherapy, Active/ | 160514 |
| 7 | (vaccin* or immuni* or inocul*).ti,ab,kf. | 834488 |
| 8 | 5 or 6 or 7 | 870327 |
| 9 | 4 and 8 | 39133 |
| 10 | influenza vaccines/ | 26853 |
| 11 | ((vaccin* or immuni* or inocul*) adj4 influenza).ti,ab,kf. | 29925 |
| 12 | 9 or 10 or 11 | 46127 |
| 13 | ((trivalent or quadrivalent or tetravalent or tetra*) adj8 vaccin*).ti,ab,kf. | 6642 |
| 14 | (((high adj2 dose*) or highdose) adj8 vaccin*).ti,ab,kf. | 1079 |
| 15 | (TIV or QIV).ti,ab,kf. | 1075 |
| 16 | (cell adj3 vaccin*).ti,ab,kf. | 11331 |
| 17 | ((adjuvant* or squalene* or emulsion*) adj8 vaccin*).ti,ab,kf. | 16599 |
| 18 | (recombinant adj6 vaccin*).ti,ab,kf. | 15694 |
| 19 | (MF59* or "MF-59").ti,ab,kf. | 732 |
| 20 | Nucleic Acid-Based Vaccines/ or mRNA Vaccines/ | 1137 |
| 21 | ((gene or genetic or nucleic acid or RNA or mRNA) adj3 vaccin*).ti,ab,kf. | 13577 |
| 22 | 13 or 14 or 15 or 16 or 17 or 18 or 19 or 20 or 21 | 59710 |
| 23 | 12 and 22 | 6639 |
| 24 | (fluad or fluzone or flucelvax or flublok or supemtek or optaflu).ti,ab,kf,nm. | 271 |
| 25 | ((cell-based or cell-derived or cellular or "whole cell" or "whole cells") adj3 vaccin* adj6 influenza).ti,ab,kf. | 125 |
| 26 | (aIIV3 or "aIIV4 HD-IIV3" or "HD-IIV4" or ccIIV3 or ccIIV4 or RIV3 or RIV4 or aQIV).ti,ab,kf. | 80 |
| 27 | 23 or 24 or 25 or 26 | 6732 |
| 28 | limit 27 to yr="2020 -Current" | 1408 |
| 29 | animals/ not humans/ | 5107621 |
| 30 | (rat or rats or mouse or mice or swine or porcine or murine or sheep or lambs or pigs or piglets or rabbit or rabbits or cat or cats or dog or dogs or cattle or bovine or monkey or monkeys or trout or marmoset* or equine or uquines or "ex vivo" or "in vitro").ti. and (Animal Experimentation/ or models, biological/ or disease models, animal/ or exp In Vitro Techniques/ or cytological techniques/ or exp cell culture techniques/) | 402856 |
| 31 | 29 or 30 | 5213363 |
| 32 | 28 not 31 | 1229 |
| 33 | (randomized controlled trial or controlled clinical trial).pt. | 687755 |
| 34 | (randomized or randomised or placebo or randomly or trial or groups).ab. | 3535662 |
| 35 | 33 or 34 | 3676435 |
| 36 | 32 and 35 | 384 |
| 37 | exp cohort studies/ or exp epidemiologic studies/ or exp clinical trial/ or exp evaluation studies as topic/ or exp statistics as topic/ | 6599991 |
| 38 | ((control and (group* or study)) or (time and factors) or program or survey* or ci or cohort or comparative stud* or evaluation studies or follow-up*).mp. | 8617974 |
| 39 | 37 or 38 | 11344166 |
| 40 | 32 and 39 | 617 |
| 41 | (case adj2 control).ti,ab,kf. | 160691 |
| 42 | ((test-negative or test-negativity) adj6 (study or studies or design*)).ti,ab,kf. | 755 |
| 43 | 41 or 42 | 161027 |
| 44 | 32 and 43 | 40 |
| 45 | 36 or 40 or 44 | 719 |
| 46 | comment.pt. | 1014506 |
| 47 | editorial.pt. | 658111 |
| 48 | case reports.pt. | 2347767 |
| 49 | (case adj (study or studies or series or report or reports)).ti. | 415260 |
| 50 | 46 or 47 or 48 or 49 | 3912415 |
| 51 | 45 not 50 | 709 |
| 52 | (("COVID-19" or "SARS-CoV-2" or "Coronavirus Disease 2019") not "influenza").ti. | 292163 |
| 53 | exp covid-19/ or SARS-CoV-2/ | 239107 |
| 54 | 52 or 53 | 339554 |
| 55 | 51 not 54 | 605 |

**Embase (via Ovid)**

Date run: 24.07.2023

| **#** | **Searches** | **Results** |
| --- | --- | --- |
| 1 | exp influenza/ | 116528 |
| 2 | influenza.ti. | 92329 |
| 3 | exp Influenza virus A/ or influenza virus B/ | 25654 |
| 4 | 1 or 2 or 3 | 154279 |
| 5 | vaccine/ | 85225 |
| 6 | conjugate vaccine/ or inactivated vaccine/ or live vaccine/ or nucleic acid vaccine/ or recombinant vaccine/ or subunit vaccine/ or virosome vaccine/ or virus like particle vaccine/ or virus vaccine/ | 54768 |
| 7 | immunization/ or vaccination/ or active immunization/ or immunoprophylaxis/ or mass immunization/ | 347400 |
| 8 | (vaccin* or immuni* or inocul*).ti,ab,kf. | 1051488 |
| 9 | 5 or 6 or 7 or 8 | 1125136 |
| 10 | 4 and 9 | 58931 |
| 11 | ((vaccin* or immuni* or inocul*) adj4 influenza).ti,ab,kf. | 38268 |
| 12 | influenza vaccine/ | 46635 |
| 13 | 11 or 12 | 58440 |
| 14 | 10 or 13 | 76555 |
| 15 | ((trivalent or quadrivalent or tetravalent or tetra*) adj8 vaccin*).ti,ab,kf. | 8775 |
| 16 | (((high adj2 dose*) or highdose) adj8 vaccin*).ti,ab,kf. | 1408 |
| 17 | (TIV or QIV).ti,ab,kf. | 1556 |
| 18 | ((cell-based or cell-derived or cellular or "whole cell" or "whole cells") adj3 vaccin* adj6 influenza).ti,ab,kf. | 161 |
| 19 | (cell adj3 vaccin*).ti,ab,kf. | 14947 |
| 20 | cell-based vaccine/ | 353 |
| 21 | ((adjuvant* or squalene* or emulsion*) adj8 vaccin*).ti,ab,kf. | 20599 |
| 22 | (recombinant adj6 vaccin*).ti,ab,kf. | 18356 |
| 23 | recombinant vaccine/ | 8001 |
| 24 | (aIIV3 or "aIIV4 HD-IIV3" or "HD-IIV4" or ccIIV3 or ccIIV4 or RIV3 or RIV4 or aQIV).ti,ab,kf. | 89 |
| 25 | (MF59 or "MF-59").ti,ab,kf. | 885 |
| 26 | exp rna vaccine/ or nucleic acid vaccine/ | 16297 |
| 27 | ((gene or genetic or nucleic acid or RNA or mRNA) adj3 vaccin*).ti,ab,kf. | 16815 |
| 28 | 15 or 16 or 17 or 18 or 19 or 20 or 21 or 22 or 23 or 24 or 25 or 26 or 27 | 86613 |
| 29 | 14 and 28 | 10275 |
| 30 | (fluad or fluzone or flucelvax or flublok or supemtek or optaflu).ti,ab,kf. | 338 |
| 31 | (aIIV3 or "aIIV4 HD-IIV3" or "HD-IIV4" or ccIIV3 or ccIIV4 or RIV3 or RIV4 or aQIV).ti,ab,kf. | 89 |
| 32 | ((cell-based or cell-derived or cellular or "whole cell" or "whole cells") adj3 vaccin* adj6 influenza).ti,ab,kf. | 161 |
| 33 | 30 or 31 or 32 | 543 |
| 34 | 29 or 33 | 10369 |
| 35 | limit 34 to yr="2020 -Current" | 2620 |
| 36 | (rat or rats or mouse or mice or swine or porcine or murine or sheep or lambs or pigs or piglets or rabbit or rabbits or cat or cats or dog or dogs or cattle or bovine or monkey or monkeys or trout or marmoset* or equine).ti. and (exp animal experiment/ or exp in vitro study/ or exp biological model/) | 1749669 |
| 37 | animal experiment/ not (human experiment/de or human/) | 2550346 |
| 38 | exp in vitro study/ not exp in vivo study/ | 3539450 |
| 39 | exp veterinary study/ not (human experiment/de or human/) | 84 |
| 40 | 36 or 37 or 38 or 39 | 6226442 |
| 41 | 35 not 40 | 2102 |
| 42 | randomized controlled trial/ | 777203 |
| 43 | controlled clinical trial/ | 470881 |
| 44 | random*.ti,ab. | 1965836 |
| 45 | randomization/ | 98226 |
| 46 | intermethod comparison/ | 298666 |
| 47 | placebo*.ti,ab. | 370124 |
| 48 | (compare or compared or comparison).ti. | 631873 |
| 49 | ((evaluated or evaluate or evaluating or assessed or assess) and (compare or compared or comparing or comparison)).ab. | 2753018 |
| 50 | (open adj1 label).ti,ab. | 107633 |
| 51 | ((double or single or doubly or singly) adj1 (blind or blinded or blindly)).ti,ab. | 277996 |
| 52 | double blind procedure/ | 211385 |
| 53 | (parallel adj1 group*).ti,ab. | 32449 |
| 54 | (crossover or "cross over").ti,ab. | 125039 |
| 55 | ((assign* or match or matched or allocation) adj6 (alternate or group or groups or intervention or interventions or patient or patients or subject or subjects or participant or participants)).ti,ab. | 457167 |
| 56 | (assigned or allocated).ti,ab. | 488550 |
| 57 | (controlled adj8 (study or design or trial)).ti,ab. | 456608 |
| 58 | (volunteer or volunteers).ti,ab. | 287193 |
| 59 | human experiment/ | 634989 |
| 60 | trial.ti. | 404863 |
| 61 | 42 or 43 or 44 or 45 or 46 or 47 or 48 or 49 or 50 or 51 or 52 or 53 or 54 or 55 or 56 or 57 or 58 or 59 or 60 | 6360891 |
| 62 | exp cohort analysis/ | 1023923 |
| 63 | exp comparative study/ | 1705484 |
| 64 | controlled study/ or exp case control study/ or exp controlled clinical trial/ | 9942972 |
| 65 | clinical study/ or exp case control study/ or exp clinical trial/ or exp community trial/ or exp intervention study/ or exp major clinical study/ or exp postmarketing surveillance/ or exp prospective study/ or exp retrospective study/ | 7381597 |
| 66 | ((control or controlled or compare* or compara*) adj8 (group or groups or population or populations or intervention or interventions or patient or patients or subject or subjects or participant or participants or program* or vaccin*)).ti,ab,kf. | 3487545 |
| 67 | (control or controlled or compare* or compara*).ti,ab,kf. and (epidemiology/ or observational study/) | 220940 |
| 68 | ((control or controlled or compare* or compara* or evaluat*) adj8 stud*).ti,ab,kf. | 2821254 |
| 69 | follow up/ or follow-up.ti,ab,kf. | 2578286 |
| 70 | cohort.ti,ab,kf. | 1330861 |
| 71 | (case adj2 control).ti,ab,kf. | 214060 |
| 72 | ((test-negative or test negativity) adj4 (study or studies or design*)).ti,ab,kf. | 866 |
| 73 | 62 or 63 or 64 or 65 or 66 or 67 or 68 or 69 or 70 or 71 or 72 | 17357049 |
| 74 | 61 or 73 | 18738508 |
| 75 | 41 and 74 | 1315 |
| 76 | comment/ or editorial/ | 742623 |
| 77 | limit 75 to (editorial or erratum or letter or note) | 39 |
| 78 | exp case study/ | 104758 |
| 79 | (case adj (study or studies or series or report or reports)).ti. | 534084 |
| 80 | 76 or 77 or 78 or 79 | 1346968 |
| 81 | 75 not 80 | 1256 |
| 82 | (("COVID-19" or "SARS-CoV-2" or "Coronavirus Disease 2019") not "influenza").ti. | 316922 |
| 83 | (exp Severe acute respiratory syndrome coronavirus 2/ or exp coronavirus disease 2019/) not (exp influenza/ or exp Influenza virus A/ or influenza virus B/) | 347784 |
| 84 | 82 or 83 | 402127 |
| 85 | 81 not 84 | 956 |

# Appendix B – Publications excluded by full-text

**Non-eligible outcomes (n= 51)**

1. Belongia EA, Levine MZ, Olaiya O, Gross FL, King JP, Flannery B, McLean HQ. Clinical trial to assess immunogenicity of high-dose, adjuvanted, and recombinant influenza vaccines against cell-grown A(H3N2) viruses in adults 65 to 74 years, 2017-2018. Vaccine. 2020;38(15):3121-8.
2. Boikos C, Fischer L, O'Brien D, Vasey J, Sylvester GC, Mansi JA. Relative Effectiveness of the Cell-derived Inactivated Quadrivalent Influenza Vaccine Versus Egg-derived Inactivated Quadrivalent Influenza Vaccines in Preventing Influenza-related Medical Encounters during the 2018-2019 Influenza Season in the United Sta. Clinical Infectious Diseases. 2021;73(3):E692-E8.
3. Boikos C, Fischer L, O'Brien D, Vasey J, Sylvester GC, Mansi JA. Relative Effectiveness of Adjuvanted Trivalent Inactivated Influenza Vaccine Versus Egg-derived Quadrivalent Inactivated Influenza Vaccines and High-dose Trivalent Influenza Vaccine in Preventing Influenza-related Medical Encounters in US Adults >= 65 Yea. Clinical Infectious Diseases. 2021;73(5):816-23.
4. Boikos C, Imran M, Nguyen VH, Ducruet T, Sylvester GC, Mansi JA. Effectiveness of the adjuvanted influenza vaccine in older adults at high risk of influenza complications. Vaccines. 2021;9(8):862.
5. Boikos C, Imran M, Nguyen VH, Ducruet T, Sylvester GC, Mansi JA. Effectiveness of the Cell-Derived Inactivated Quadrivalent Influenza Vaccine in Individuals at High Risk of Influenza Complications in the 2018-2019 United States Influenza Season. Open Forum Infectious Diseases. 2021;8(7):ofab167.
6. Boikos C, McGovern I, Ortiz JR, Puig-Barbera J, Versage E, Haag M. Relative Vaccine Effectiveness of Adjuvanted Trivalent Influenza Vaccine over Three Consecutive Influenza Seasons in the United States. Vaccines. 2022;10(9):1456.
7. Boikos C, Sylvester GC, Sampalis JS, Mansi JA. Relative effectiveness of the cell-cultured quadrivalent influenza vaccine compared to standard, egg-derived quadrivalent influenza vaccines in preventing influenza-like illness in 2017-2018. Clinical Infectious Diseases. 2020;71(10):E665-E71.
8. Butler AM, Layton JB, Dharnidharka VR, Sahrmann JM, Seamans MJ, Weber DJ, McGrath LJ.

Comparative Effectiveness of High-Dose Versus Standard-Dose Influenza Vaccine Among Patients Receiving Maintenance Hemodialysis. American Journal of Kidney Diseases. 2020;75(1):72-83.

1. Chaves SS, Naeger S, Lounaci K, Zuo Y, Loiacono MM, Pilard Q, et al. High-dose influenza vaccine is associated with reduced mortality among older adults with breakthrough influenza even when there is poor vaccine-strain match. Clinical infectious diseases : an official publication of the Infectious Diseases Society of America. 2023((Chaves, Naeger, Mahe) Sanofi Vaccines, Lyon, France(Lounaci, Zuo, Pilard, Genin) Quinten Health, Lyon, France(Loiacono) Sanofi Vaccines, PA, United States(Nealon) School of Public Health, Li Ka Shing Faculty of Medicine, University of Hong Kong, Hong Kon).
2. Cocchio S, Gallo T, Zotto SD, Clagnan E, Iob A, Furlan P, et al. Preventing the risk of hospitalization for respiratory complications of influenza among the elderly: Is there a better influenza vaccination strategy? a retrospective population study. Vaccines. 2020;8(3):1-11.
3. Davidson H, Han L, McConeghy K, Saade E, Canaday D, Mor V, Gravenstein S. Can adjuvanted influenza vaccine given as standard of care reduce the risk for influenza outbreaks in nursing homes? evidence from a cluster-randomized trial of 823 nursing homes. Journal of the American Geriatrics Society. 2020;68(SUPPL 1):S217.
4. Dawood FS, Naleway AL, Flannery B, Levine MZ, Murthy K, Sambhara S, et al. Comparison of the Immunogenicity of Cell Culture-Based and Recombinant Quadrivalent Influenza Vaccines to Conventional Egg-Based Quadrivalent Influenza Vaccines Among Healthcare Personnel Aged 18-64 Years: A Randomized Open-Label Trial. Clinical Infectious Diseases. 2021;73(11):1973-81.
5. Divino V, Krishnarajah G, Pelton SI, Mould-Quevedo J, Anupindi VR, DeKoven M, Postma MJ. A real-world study evaluating the relative vaccine effectiveness of a cell-based quadrivalent influenza vaccine compared to egg-based quadrivalent influenza vaccine in the US during the 2017-18 influenza season. Vaccine. 2020;38(40):6334-43.
6. Divino V, Postma M, Pelton SI, Mould-Quevedo JF, Anupindi R, DeKoven M, Levin MJ. Relative Vaccine Effectiveness Against Influenza-Related and Any Respiratory-Related Hospital Encounter during the 2019/20 High Influenza Activity Period: A Comprehensive Real-World Analysis to Compare Quadrivalent Cell-based and Egg-based Influenza Vacci. Open Forum Infectious Diseases. 2021;8(SUPPL 1):S61.
7. Divino V, Ruthwik Anupindi V, Dekoven M, Mould-Quevedo J, Pelton SI, Postma MJ, Levin MJ. A Real-World Clinical and Economic Analysis of Cell-Derived Quadrivalent Influenza Vaccine Compared to Standard Egg-Derived Quadrivalent Influenza Vaccines during the 2019-2020 Influenza Season in the United States. Open Forum Infectious Diseases. 2022;9(1):ofab604.
8. Gravenstein S, McConeghy K, Davidson H, Han L, Canaday D, Saade E, Mor V. Secondary analysis comparative effectiveness of adjuvanted vs non-adjuvanted influenza vaccine on reducing hospital days in a long-term care population. Journal of the American Geriatrics Society. 2020;68(SUPPL 1):S218.
9. Gravenstein S, McConeghy KW, Saade E, Davidson HE, Canaday DH, Han L, et al. Adjuvanted Influenza Vaccine and Influenza Outbreaks in US Nursing Homes: Results From a Pragmatic Cluster-Randomized Clinical Trial. Clinical Infectious Diseases. 2021;73(11):E4229-E36.
10. Helene B, Marie-Cecile L, Nada A, Pascal C, Alexandre D, Karine M, et al. The relative effectiveness of a high-dose quadrivalent influenza vaccine vs standard-dose quadrivalent influenza vaccines in older adults in France: a retrospective cohort study during the 2021-22 influenza season. medRxiv. 2023((Helene, Marie-Cecile, Karine, Ayman) Sanofi Vaccines, France(Nada, Benjamin, Fanny) HEVA, Pole Epidemiologie, Lyon, France(Pascal) Ecole des hautes etudes en sante publique, CNRS, Universite de Rennes, ARENES - UMR 6051, Recherche sur les services et le).
11. Imran M, Ortiz JR, McLean HQ, Fisher L, O'Brien D, Bonafede M, et al. Relative Effectiveness of Cell-Based Versus Egg-Based Quadrivalent Influenza Vaccines in Adults During the 2019-2020 Influenza Season in the United States. Open Forum Infectious Diseases. 2022;9(10):ofac532.
12. Imran M, Puig-Barbera J, Ortiz JR, Fischer L, O'Brien D, Bonafede M, et al. Relative Effectiveness of MF59 Adjuvanted Trivalent Influenza Vaccine vs Nonadjuvanted Vaccines during the 2019-2020 Influenza Season. Open Forum Infectious Diseases. 2022;9(5):ofac167.
13. Izurieta HS, Chillarige Y, Kelman J, Wei Y, Lu Y, Xu W, et al. Relative Effectiveness of Influenza Vaccines among the United States Elderly, 2018-2019. Journal of Infectious Diseases. 2020;222(2):278-87.
14. Izurieta HS, Lu M, Kelman J, Lu Y, Lindaas A, Loc J, et al. Comparative Effectiveness of Influenza Vaccines Among US Medicare Beneficiaries Ages 65 Years and Older During the 2019-2020 Season. Clinical Infectious Diseases. 2021;73(11):E4251-E9.
15. Krishnarajah G, Divino V, Postma MJ, Pelton SI, Anupindi VR, Dekoven M, Mould-Quevedo J. Clinical and economic outcomes associated with cell-based quadrivalent influenza vaccine vs. Standard-dose egg-based quadrivalent influenza vaccines during the 2018-19 influenza season in the United States. Vaccines. 2021;9(2):1-17.
16. Lapi F, Domnich A, Marconi E, Rossi A, Cricelli C. Adjuvanted versus non-adjuvanted standard-dose influenza vaccines in preventing all-cause hospitalizations in the elderly: a cohort study with nested case-control analyses over 18 influenza seasons. Expert Review of Vaccines. 2022;21(11):1647-53.
17. Lee H, Hong B, Kim S, Kim JH, Choi NK, Jung SY, Shin JY. Post-marketing surveillance study on influenza vaccine in South Korea using a nationwide spontaneous reporting database with multiple data mining methods. Scientific reports. 2022;12(1):20256.
18. Levin MJ, Divino V, Pelton SI, Postma M, Shah D, Mould-Quevedo JF, DeKoven M. Relative Effectiveness of Adjuvanted Trivalent Influenza Vaccine Compared to Egg-Based Trivalent High-Dose Influenza Vaccine among U.S. Older Adults during 2019-20 Influenza Season. Open Forum Infectious Diseases. 2021;8(SUPPL 1):S136.
19. Levin MJ, Divino V, Shah D, Dekoven M, Mould-Quevedo J, Pelton SI, Postma MJ. Comparing the clinical and economic outcomes associated with adjuvanted versus high-dose trivalent influenza vaccine among adults aged >= 65 years in the us during the 2019-20 influenza season-a retrospective cohort analysis. Vaccines. 2021;9(10):1146.
20. Liberg R, Schofield C, Richard SA, Collins L, Spooner C, Seshadri S, et al. Impact of COVID-19 Pandemic on Influenza-like Illness (ILI) Experience among Healthcare Workers in Military Treatment Facilities. Open Forum Infectious Diseases. 2022;9(Supplement 2):S895.
21. Loeb N, Andrew MK, Loeb M, Kuchel GA, Haynes L, McElhaney JE, Verschoor CP. Frailty is associated with increased hemagglutination- inhibition titers in a 4-year randomized trial comparing standard- And high-dose influenza vaccination. Open Forum Infectious Diseases. 2020;7(5):ofaa148.
22. MacHado MA, Moura CS, Abrahamowicz M, Ward BJ, Pilote L, Bernatsky S. Better outcomes with high-dose trivalent influenza vaccine in seniors. Pharmacoepidemiology and Drug Safety. 2021;30(SUPPL 1):88-9.
23. Machado MAA, Moura CS, Abrahamowicz M, Ward BJ, Pilote L, Bernatsky S. Relative effectiveness of influenza vaccines in elderly persons in the United States, 2012/2013-2017/2018 seasons. npj Vaccines. 2021;6(1):108.
24. McConeghy KW, Davidson HE, Canaday DH, Han L, Saade E, Mor V, Gravenstein S. Cluster-randomized Trial of Adjuvanted Versus Nonadjuvanted Trivalent Influenza Vaccine in 823 US Nursing Homes. Clinical Infectious Diseases. 2021;73(11):E4237-E43.
25. McElhaney JE, Verschoor CP, Haynes L, Pawelec G, Loeb M, Andrew MK, Kuchel GA. Key Determinants of Cell-Mediated Immune Responses: A Randomized Trial of High Dose Vs. Standard Dose Split-Virus Influenza Vaccine in Older Adults. Frontiers in aging. 2021;2(9918231199706676).
26. McLean HQ, Levine MZ, King JP, Flannery B, Belongia EA. Serologic response to sequential vaccination with enhanced influenza vaccines: Open label randomized trial among adults aged 65-74 years. Vaccine. 2021;39(49):7146-52.
27. Naleway AL, Kim SS, Flannery B, Levine MZ, Murthy K, Sambhara S, et al. Immunogenicity of High-Dose Egg-Based, Recombinant, and Cell Culture-Based Influenza Vaccines Compared With Standard-Dose Egg-Based Influenza Vaccine Among Health Care Personnel Aged 18-65 Years in 2019-2020. Open Forum Infectious Diseases. 2023;10(6):ofad223.
28. Paudel M, Mahmud S, Buikema A, Korrer S, Van Voorhis D, Brekke L, Chit A. Relative vaccine efficacy of high-dose versus standard-dose influenza vaccines in preventing probable influenza in a Medicare Fee-for-Service population. Vaccine. 2020;38(29):4548-56.
29. Pelton S, Postma M, Divino V, Mould J, Dekoven M, Krishnarajah G. Relative vaccine effectiveness of adjuvanted trivalent influenza vaccine compared to egg-based trivalent high dose and other egg-based vaccines. International Journal of Infectious Diseases. 2020;101(Supplement 1):465.
30. Pelton S, Postma M, Divino V, Mould-Quevedo JF, DeKoven M, Krishnarajah G. PIN3 RELATIVE VACCINE EFFECTIVENESS OF QUADRIVALENT CELL-BASED VERSUS EGG-BASED INFLUENZA VACCINES AMONG ADULTS 50-64 YEARS OLD: A U.S. OBSERVATIONAL COHORT STUDY. Value in Health. 2020;23(Supplement 1):S168.
31. Pelton SI, Divino V, Postma MJ, Shah D, Mould-Quevedo J, DeKoven M, Krishnarajah G. A retrospective cohort study assessing relative effectiveness of adjuvanted versus high-dose trivalent influenza vaccines among older adults in the United States during the 2018-19 influenza season. Vaccine. 2021;39(17):2396-407.
32. Pelton SI, Divino V, Shah D, Mould-Quevedo J, Dekoven M, Krishnarajah G, Postma MJ. Evaluating the relative vaccine effectiveness of adjuvanted trivalent influenza vaccine compared to high-dose trivalent and other egg-based influenza vaccines among older adults in the us during the 2017-2018 influenza season. Vaccines. 2020;8(3):1-17.
33. Pelton SI, Postma M, Divino V, Mould-Quevedo JF, Anupindi R, DeKoven M, Levin MJ. Relative Vaccine Effectiveness Against Influenza-related Hospitalizations and Respiratory Events during the 2019/20 Influenza seAson in U.S. Children and Adults. A Real-World Evidence Comparison between Quadrivalent Cell-based and Egg-based Influenza Vacc. Open Forum Infectious Diseases. 2021;8(SUPPL 1):S758.
34. Pelton SI, Postma M, Divino V, Shah D, Mould-Quevedo JF, DeKoven M, Krishnarajah G. Mf59 assurance 2: A real-world study to estimate the relative vaccine effectiveness of adjuvanted trivalent influenza vaccine compared to egg-based trivalent high-dose among u.s. older adults during 2018-19 influenza season. Open Forum Infectious Diseases. 2020;7(SUPPL 1):S3.
35. Postma M, Pelton SI, Divino V, Mould-Quevedo JF, Shah D, DeKoven M. A comprehensive real-world analysis to compare adjuvanted trivalent influenza vaccine and trivalent high dose influenza vaccine by age and period of high influenza activity for the 2018-19 season among u.s. elderly. Open Forum Infectious Diseases. 2020;7(SUPPL 1):S29-S30.
36. Richard SA, Schofield C, Collins L, Spooner C, Seshadri S, Ganesan A, et al. Pathogen Co-infections and Trends in Influenza-like Illness in PAIVED. Open Forum Infectious Diseases. 2022;9(Supplement 2):S897.
37. Saade E, Davidson H, McConeghy K, Han L, Mor V, Canaday D, Gravenstein S. Effectiveness of adjuvanted vs non-adjuvanted influenza vaccination in U.S. Nursing homes. Journal of the American Geriatrics Society. 2020;68(SUPPL 1):S57.
38. Saade EA, Abul Y, McConeghy K, Edward Davidson H, Han L, Joyce N, et al. High-dose influenza vaccines for the prevention of hospitalization due to cardiovascular events in older adults in the nursing home: Post-hoc analysis of a cluster-randomized trial. Vaccine. 2022;40(47):6700-5.
39. Sanchez-de Prada L, Sanz-Munoz I, de Lejarazu RO, Eiros JM, Garcia-Sastre A, Aydillo T. Immunodominance hierarchy after seasonal influenza vaccination. Emerging Microbes and Infections. 2022;11(1):2670-9.
40. Smith CL, Bednarchik B, Aung H, Wilk DJ, Boxer RS, Daddato AE, et al. Humoral and Cellular Immunity Induced by Adjuvanted and Standard Trivalent Influenza

Vaccine in Older Nursing Home Residents. The Journal of infectious diseases. 2023((Smith) Department of Pathology, Case Western Reserve University School of Medicine, Cleveland 44106, United States(Bednarchik) Department of Medicine, University Hospitals Case Medical Center, Cleveland 44106, United States(Aung, Wilk) Case Western Reser).

1. van Aalst R, Gravenstein S, Mor V, Mahmud SM, Wilschut J, Postma M, Chit A. Comparative effectiveness of high dose versus adjuvanted influenza vaccine: A retrospective cohort study. Vaccine. 2020;38(2):372-9.
2. Vardeny O, Kim K, Udell JA, Joseph J, Desai AS, Farkouh ME, et al. Effect of High-Dose Trivalent vs Standard-Dose Quadrivalent Influenza Vaccine on Mortality or Cardiopulmonary Hospitalization in Patients with High-risk Cardiovascular Disease: A Randomized Clinical Trial. JAMA - Journal of the American Medical Association. 2021;325(1):39-49.
3. Young-Xu Y, Snider JT, Mahmud SM, Russo EM, van Aalst R, Thommes EW, et al. High-dose influenza vaccination and mortality among predominantly male, white, senior veterans, United States, 2012/13 to 2014/15. Eurosurveillance. 2020;25(19).

**Non-eligible intervention (n=28)**

1. Basu I, Supe PD, Agarwal M. Comparison of Immunogenicity and Safety between Two Quadrivalent Influenza Vaccines in Healthy Indian Adults (18-60 Years) and Elderly (? 61 Years) - A Phase III, Active-Controlled, Randomized Clinical Study. The Journal of the Association of Physicians of India. 2020;68(1):105.
2. Branagan AR, Duffy E, Gan G, Li F, Foster C, Verma R, et al. Tandem high-dose influenza vaccination is associated with more durable serologic immunity in patients with plasma cell dyscrasias. Blood Advances. 2021;5(5):1535-9.
3. Cross JW, Joy M, McGee C, Akinyemi O, Gatenby P, de Lusignan S. Adverse events of interest vary by influenza vaccine type and brand: Sentinel network study of eight seasons (2010-2018). Vaccine. 2020;38(22):3869-80.
4. Drori Y, Pando R, Sefty H, Rosenberg A, Mendelson E, Keinan-Boker L, et al. Influenza vaccine effectiveness against laboratory-confirmed influenza in a vaccine-mismatched influenza B-dominant season. Vaccine. 2020;38(52):8387-95.
5. El Sahly HM, Yildirim I, Frey SE, Winokur P, Jackson LA, Bernstein DI, et al. Safety and Immunogenicity of a Delayed Heterologous Avian Influenza A(H7N9) Vaccine Boost Following Different Priming Regimens: A Randomized Clinical Trial. The Journal of infectious diseases. 2023(ih3, 0413675).
6. Endo M, Tanishima M, Ibaragi K, Hayashida K, Fukuda T, Tanabe T, et al. Clinical phase II and III studies of an AS03-adjuvanted H5N1 influenza vaccine produced in an EB66 R cell culture platform. Influenza and other respiratory viruses. 2020;14(5):551-63.
7. Fadlyana E, Dhamayanti M, Tarigan R, Prodjosoewojo S, Rahmadi AR, Sari RM, et al. Immunogenicity and safety of Quadrivalent Influenza HA vaccine compared with Trivalent Influenza HA vaccine and evaluation of Quadrivalent Influenza HA vaccine batch-to-batch consistency in Indonesian children and adults. medRxiv. 2023((Fadlyana, Dhamayanti, Tarigan, Rusmil, Kartasasmita) Department of Child Health, Faculty of Medicine, Universitas Padjadjaran, Hasan Sadikin Hospital, Bandung, Indonesia(Prodjosoewojo, Rahmadi) Department of Internal Medicine, Faculty of Medicine, Univer).
8. Folschweiller N, Vanden Abeele C, Chu L, Van Damme P, Garcia-Sastre A, Krammer F, et al. Reactogenicity, safety, and immunogenicity of chimeric haemagglutinin influenza split-virion vaccines, adjuvanted with AS01 or AS03 or non-adjuvanted: a phase 1-2 randomised controlled trial. The Lancet Infectious Diseases. 2022;22(7):1062-75.
9. Fonseca HAR, Furtado RHM, Zimerman A, Lemos PA, Franken M, Monfardini F, et al. Influenza vaccination strategy in acute coronary syndromes: the VIP-ACS trial. European Heart Journal. 2022;43(41):4378-88.
10. Gaglani M, Vasudevan A, Raiyani C, Murthy K, Chen W, Reis M, et al. Effectiveness of Trivalent and Quadrivalent Inactivated Vaccines against Influenza B in the United States, 2011-2012 to 2016-2017. Clinical Infectious Diseases. 2021;72(7):1147-57.
11. Gorse GJ, Grimes S, Buck H, Mulla H, White P, Hill H, et al. A phase 1 dose-sparing, randomized clinical trial of seasonal trivalent inactivated influenza vaccine combined with MAS-1, a novel water-in-oil adjuvant/delivery system. Vaccine. 2022;40(9):1271-81.
12. Gorse GJ, Grimes S, Buck H, Mulla H, White P, Hill H, et al. MAS-1, a novel water-in-oil adjuvant/delivery system, with reduced seasonal influenza vaccine hemagglutinin dose may enhance potency, durability and cross-reactivity of antibody responses in the elderly. Vaccine. 2022;40(10):1472-82.
13. Hsu PS, Lian IB, Chao DY. A population-based propensity score-matched study to assess the impact of repeated vaccination on vaccine effectiveness for influenza-associated hospitalization among the elderly. Clinical Interventions in Aging. 2020;15((Hsu) Department of Family Medicine, Taichung Hospital, Ministry of Health and Welfare, Taichung, Taiwan (Republic of China)(Hsu, Chao) Graduate Institute of Microbiology and Public Health, College of Veterinary Medicine, National Chung-Hsing University):301-12.
14. Kumabe A, Kenzaka T, Yahata S, Goda K, Okayama M. Evaluation of Adverse Reactions to Influenza Vaccination: A Prospective Cohort Study. Vaccines. 2022;10(10):1664.
15. Leroux-Roels I, Waerlop G, Tourneur J, De Boever F, Maes C, Bruhwyler J, et al. Randomized, Double-Blind, Reference-Controlled, Phase 2a Study Evaluating the Immunogenicity and Safety of OVX836, A Nucleoprotein-Based Influenza Vaccine. Frontiers in Immunology. 2022;13((Leroux-Roels, Waerlop, De Boever, Maes, Leroux-Roels) Center for Vaccinology (CEVAC), Ghent University and University Hospital, Ghent, Belgium(Tourneur, Bruhwyler, Guyon-Gellin, Moris, Del Campo, Willems, Le Vert, Nicolas) OSIVAX, Lyon, France):852904.
16. Mir H, Haq I, Koul PA. Poor vaccine effectiveness against influenza b-related severe acute respiratory infection in a temperate north indian state (2019-2020): A call for further data for possible vaccines with closer match. Vaccines. 2021;9(10):1094.
17. Neuzil KM, Anderson EJ, Frenck RW, Frey SE, Walter EB, Rupp R, et al. Safety and Immunogenicity of Influenza A/H5N8 Virus Vaccine in Healthy Adults: Durability and Cross-reactivity of Antibody Responses. Clinical infectious diseases : an official publication of the Infectious Diseases Society of America. 2023((Neuzil, Rotrosen, Ortiz) Center for Vaccine Development and Global Health, University of Maryland, Baltimore, MD, United States(Anderson) Departments of Pediatrics and Medicine, Emory University School of Medicine, Atlanta, GA, United States(Frenck, Brad).
18. Ritveeradej E, Boonnak K, Yoowannakul S. Efficacy of double-dose influenza vaccine with a booster compared with standard dose in hemodialysis patients: Randomized controlled trial. Journal of the American Society of Nephrology. 2020;31((Ritveeradej, Yoowannakul) Bhumibol Adulyadej Hospital, Bangkok, Thailand(Boonnak) Mahidol University, Faculty of Tropical Medicine, Bangkok, Thailand):363.
19. Shasha D, Valinsky L, Hershkowitz Sikron F, Glatman-Freedman A, Mandelboim M, Toledano A, et al. Quadrivalent versus trivalent influenza vaccine: clinical outcomes in two influenza seasons, historical cohort study. Clinical Microbiology and Infection. 2020;26(1):101-6.
20. Shinde V, Cai R, Plested J, Cho I, Fiske J, Pham X, et al. Induction of Cross-Reactive Hemagglutination Inhibiting Antibody and Polyfunctional CD4+ T-Cell Responses by a Recombinant Matrix-M-Adjuvanted Hemagglutinin Nanoparticle Influenza Vaccine. Clinical Infectious Diseases. 2021;73(11):E4278-E87.
21. Shinde V, Cho I, Plested JS, Agrawal S, Fiske J, Cai R, et al. Comparison of the safety and immunogenicity of a novel Matrix-M-adjuvanted nanoparticle influenza vaccine with a quadrivalent seasonal influenza vaccine in older adults: a phase 3 randomised controlled trial. The Lancet Infectious Diseases. 2022;22(1):73-84.
22. Teh B, Leung V, Mordant F, Sullivan S, Joyce T, Harrison S, et al. A randomised trial of two 2-dose influenza vaccination strategies for patients following autologous haematopoietic stem cell transplantation. HemaSphere. 2020;4(Supplement 1):116-7.
23. Teh BW, Leung VKY, Mordant FL, Sullivan SG, Joyce T, Harrison SJ, et al. A randomized trial of two 2-dose influenza vaccination strategies for patients following autologous hematopoietic stem cell transplantation. Clinical Infectious Diseases. 2021;73(11):E4269-E77.
24. Thompson MG, Soto G, Peretz A, Newes-Adeyi G, Yoo YM, Hirsch A, et al. Influenza vaccine effectiveness within prospective cohorts of healthcare personnel in Israel and Peru 2016-2019. Vaccine. 2021;39(47):6956-67.
25. Vanni T, da Graca Salomao M, Viscondi JYK, Braga PE, da Silva A, de Oliveira Piorelli R, et al. A randomized, double-blind, non-inferiority trial comparing the immunogenicity and safety of two seasonal inactivated influenza vaccines in adults. Vaccine. 2023;41(22):3454-60.
26. Watanabe A, Nishida S, Burcu T, Shibahara T, Kusakabe T, Kuroda E, et al. Safety and immunogenicity of a quadrivalent seasonal influenza vaccine adjuvanted with hydroxypropyl-beta-cyclodextrin: A phase 1 clinical trial. Vaccine. 2022;40(31):4150-9.
27. Winokur P, El Sahly HM, Mulligan MJ, Frey SE, Rupp R, Anderson EJ, et al. Immunogenicity and safety of different dose schedules and antigen doses of an MF59-adjuvanted H7N9 vaccine in healthy adults aged 65 years and older. Vaccine. 2021;39(8):1339-48.
28. Zheng C, Duffy J, Liu ILA, Sy LS, Chen W, Qian L, et al. Risk for Shoulder Conditions After Vaccination: A Population-Based Study Using Real-World Data. Annals of Internal Medicine. 2022;175(5):634-43.

**Non-eligible study design (n=25)**

1. Abbasi J. Pfizer Launches Phase 1 mRNA Flu Vaccine Trial. JAMA - Journal of the American Medical Association. 2021;326(18):1784.
2. Al Qahtani AA, Selim M, Hamouda NH, Al Delamy AL, Macadangdang C, Al Shammari KH, Al Shamary SF. Seasonal influenza vaccine effectiveness among health-care workers in Prince Sultan Military Medical City, Riyadh, KSA, 2018-2019. Human Vaccines and Immunotherapeutics. 2020((Al Qahtani, Selim, Hamouda, Al Delamy, Macadangdang, Al Shammari, Al Shamary) Preventive Medicine Division, Family and Community Medicine Administration, Prince Sultan Military Medical City, Riyadh, Saudi Arabia(Selim) Department of Public Health and Com):1-5.
3. Al Qahtani AA, Selim M, Hamouda NH, Al Delamy AL, Macadangdang C, Al Shammari KH, Al Shamary SF. Seasonal influenza vaccine effectiveness among health-care workers in Prince Sultan Military Medical City, Riyadh, KSA, 2018-2019. Human vaccines & immunotherapeutics. 2021;17(1):119-23.
4. Ando S. Estimation of the effectiveness of quadrivalent influenza vaccines by distinguishing between influenza a (H1N1) pdm09 and Influenza A (H3N2) using rapid influenza diagnostic tests during the 2018-2019 season. Internal Medicine. 2020;59(7):933-40.
5. Anonymous. Soon even flu vaccines based on mRNA?: Moderna and Sanofi start phase I trial. Deutsche Apotheker Zeitung. 2021;161(28):A24.
6. Bellino S, Piovesan C, Bella A, Rizzo C, Pezzotti P, Ramigni M. Determinants of vaccination uptake, and influenza vaccine effectiveness in preventing deaths and hospital admissions in the elderly population; Treviso, Italy, 2014/2015-2016/2017 seasons. Human Vaccines and Immunotherapeutics. 2020;16(2):301-12.
7. Bhatt AS, Vardeny O, Udell JA, Joseph J, Kim K, Solomon SD. Influenza vaccination: a 'shot' at INVESTing in cardiovascular health. European Heart Journal. 2021;42(20):2015-8.
8. Bianchini M, Wright GC, Anderson HD, Perraillon MC, Lindrooth RC. Impact of High-Dose Vs Standard-Dose Influenza Vaccine on Respiratory Hospitalizations Among Adults. Value in Health. 2022;25(7 Supplement):S303.
9. Burgess T, Richard SA, Collins L, Spooner C, Seshadri S, Schofield C, et al. Pragmatic Assessment of Influenza Vaccine Effectiveness in the Department of Defense (PAIVED): Updates from Year 4 of a Multi-site Trial. Open Forum Infectious Diseases. 2022;9(Supplement 2):S898-S9.
10. Dingermann T. Adjuvanted and high-dose: Comparison of influenza vaccines in the elderly. Pharmazeutische Zeitung. 2022;167(23):36-9.
11. Fallani E, Orsi A, Signori A, Icardi G, Domnich A. An exploratory study to assess patterns of influenza- and pneumonia-related mortality among the Italian elderly. Human Vaccines and Immunotherapeutics. 2021;17(12):5514-21.
12. Fruhwein M, Schelling J, Wahle K, Beier D, Kwetkat A, Schwarz TF. Enhanced targeted influenza vaccines - New evidence shows higher effectiveness in older adults. Deutsche Medizinische Wochenschrift. 2023;148(9):556-62.
13. Grohskopf LA, Blanton LH, Ferdinands JM, Chung JR, Broder KR, Talbot HK, et al. Prevention and Control of Seasonal Influenza with Vaccines: Recommendations of the Advisory Committee on Immunization Practices - United States, 2022-23 Influenza Season. MMWR Recommendations and reports : Morbidity and mortality weekly report Recommendations and reports. 2022;71(1):1-28.
14. Hollingsworth R, Palmu A, Pepin S, Dupuy M, Shrestha A, Jokinen J, et al. Effectiveness of the quadrivalent high-dose influenza vaccine for prevention of cardiovascular and respiratory events in people aged 65 years and above: Rationale and design of a real-world pragmatic randomized clinical trial. American Heart Journal. 2021;237((Hollingsworth, Samson) Global Medical Affairs, Sanofi Pasteur, Swiftwater, PA, United States(Palmu, Syrjanen) Finnish Institute for Health and Welfare, Tampere, Finland(Pepin, De Bruijn) Global Clinical Sciences, Sanofi Pasteur, Marcy L'Etoile, France(Du):54-61.
15. Kamath A, Maity N, Nayak MA. Facial Paralysis Following Influenza Vaccination: A Disproportionality Analysis Using the Vaccine Adverse Event Reporting System Database. Clinical Drug Investigation. 2020;40(9):883-9.
16. Lee H, Kim HJ, Choe YJ, Shin JY. Signals and trends of Guillain-Barre syndrome after the introduction of live-attenuated vaccines for influenza in the US and South Korean adverse event reporting systems. Vaccine. 2020;38(34):5464-73.
17. Lee IT, Nachbagauer R, Ensz D, Schwartz H, Carmona L, Schaefers K, et al. Safety and immunogenicity of a phase 1/2 randomized clinical trial of a quadrivalent, mRNA-based seasonal influenza vaccine (mRNA-1010) in healthy adults: interim analysis. Nature Communications. 2023;14(1):3631.
18. Lucero-Obusan CA, Schirmer P, Oda G, Holodniy M. Influenza Surveillance in the Veterans Health Administration (VHA): 2020-2021 Season. Open Forum Infectious Diseases. 2021;8(SUPPL 1):S803.
19. Lungu M, Telehuz A, Niculet E, Voinescu CD, Elkan EM, Trifan A, et al. Recurrent guillain barre syndrome after administration of influenza vaccine. Cases reports and update of literature data. Acta Medica Mediterranea. 2021;37(1):457-60.
20. Moro PL, Woo EJ, Marquez P, Cano M. Monitoring the safety of high-dose, trivalent inactivated influenza vaccine in the vaccine adverse event reporting system (VAERS), 2011 - 2019. Vaccine. 2020;38(37):5923-6.
21. Panatto D, Haag M, Lai PL, Tomczyk S, Amicizia D, Lino MM. Enhanced Passive Safety Surveillance (EPSS) confirms an optimal safety profile of the use of MF59-adjuvanted influenza vaccine in older adults: Results from three consecutive seasons. Influenza and other Respiratory Viruses. 2020;14(1):61-6.
22. Schmader KE, Liu CK, Flannery B, Rountree W, Auerbach H, Barnett ED, et al. Immunogenicity of adjuvanted versus high-dose inactivated influenza vaccines in older adults: a randomized clinical trial. Immunity and Ageing. 2023;20(1):30.
23. Tsai SY, Yeh TY, Chiu NC, Huang CT. National safety surveillance of quadrivalent recombinant influenza vaccine in Taiwan during NH 20/21. Vaccine. 2022;40(26):3701-4.
24. Valeri M, Wang Z, Armstrong L, Liu B. High-dose quadrivalent influenza vaccine for older populations. Medicine Today. 2022;23(4):61-3.
25. Woo EJ, Moro PL. Postmarketing safety surveillance of high-dose quadrivalent influenza vaccine: Reports to the Vaccine Adverse Event Reporting System. Vaccine. 2022;40(7):1026-30.

**Non-eligible comparator (n=16)**

1. Carreras JJ, Lluch JA, Taboada JA, Pastor-Villalba E, Nartallo-Penas V, Diez-Domingo J. Adverse events in pregnant women with the tetravalent influenza vaccine obtained from cell cultures. Enfermedades Infecciosas y Microbiologia Clinica. 2022((Carreras, Diez-Domingo) Area de Investigacion en Vacunas, FISABIO - Salud Publica, Valencia, Spain(Lluch, Pastor-Villalba) Direccion General de Salud Publica y Adicciones, Conselleria de Sanitat Universal i Salut Publica, Valencia (Comunidad Valenciana)).
2. Essink B, Fierro C, Rosen J, Figueroa AL, Zhang B, Verhoeven C, et al. Immunogenicity and safety of MF59-adjuvanted quadrivalent influenza vaccine versus standard and alternate B strain MF59-adjuvanted trivalent influenza vaccines in older adults. Vaccine. 2020;38(2):242-50.
3. Fabiani M, Volpe E, Faraone M, Bella A, Pezzotti P, Chini F. Effectiveness of influenza vaccine in reducing influenza-associated hospitalizations and deaths among the elderly population; Lazio region, Italy, season 2016-2017. Expert Review of Vaccines. 2020;19(5):479-89.
4. Fujimori M, Hasegawa S, Sasaoka S, Iguchi K, Nakamura M. A study of the association between seasonal influenza vaccines and the increased risk of Guillain-Barre syndrome using Vaccine Adverse Event Reporting System, 2018-2019. Pharmazie. 2021;76(9):437-43.
5. Fujimori M, Nakamura M. Association between seasonal influenza vaccines and the increased risk of acute disseminated encephalomyelitis, estimated using the Vaccine Adverse Event Reporting System. Die Pharmazie. 2022;77(7):262-9.
6. Gandhi-Banga S, Wague S, Shrestha A, Syrkina O, Talanova O, Nissila M, et al. Enhanced passive safety surveillance of high-dose and standard-dose quadrivalent inactivated split-virion influenza vaccines in Germany and Finland during the influenza season 2021/22. Influenza and other Respiratory Viruses. 2023;17(1):e13071.
7. Kato M, Kunkel T, Bram D, Newman J, Lopez A, Santana P, et al. Effectiveness of high dose influenza vaccine in hiv-positive patients for the winter 2017-2018 season. Open Forum Infectious Diseases. 2020;7(SUPPL 1):S36-S7.
8. Mira-Iglesias A, Lopez-Labrador FX, Garcia-Rubio J, Mengual-Chulia B, Tortajada-Girbes M, Mollar-Maseres J, et al. Influenza vaccine effectiveness and waning effect in hospitalized older adults. Valencia region, Spain, 2018/2019 season. International Journal of Environmental Research and Public Health. 2021;18(3):1-17.
9. Munoz FM, Patel SM, Jackson LA, Swamy GK, Edwards KM, Frey SE, et al. Safety and immunogenicity of three seasonal inactivated influenza vaccines among pregnant women and antibody persistence in their infants. Vaccine. 2020;38(33):5355-63.
10. Peikert A, Claggett BL, Kim KM, Udell JA, Joseph J, Desai AS, et al. Association of Post-Vaccination Adverse Reactions After Influenza Vaccine With Mortality and Cardiopulmonary Outcomes in Patients With High-Risk Cardiovascular Disease: The INVESTED Trial. Circulation. 2022;146(Supplement 1).
11. Pebody RG, Whitaker H, Ellis J, Andrews N, Marques DFP, Cottrell S, et al. End of season influenza vaccine effectiveness in primary care in adults and children in the United Kingdom in 2018/19. Vaccine. 2020;38(3):489-97.
12. Peikert A, Claggett BL, Kim K, Udell JA, Joseph J, Desai AS, et al. Association of post-vaccination adverse reactions after influenza vaccine with mortality and cardiopulmonary outcomes in patients with high-risk cardiovascular disease: the INVESTED trial. European Journal of Heart Failure. 2023;25(2):299-310.
13. Redlberger-Fritz M, Kundi M, Popow-Kraupp T. Heterogeneity of Circulating Influenza Viruses and Their Impact on Influenza Virus Vaccine Effectiveness During the Influenza Seasons 2016/17 to 2018/19 in Austria. Frontiers in Immunology. 2020;11((Redlberger-Fritz, Popow-Kraupp) Centre of Virology, Medical University Vienna, Vienna, Austria (Kundi) Department of Environmental Health, Medical University Vienna, Vienna, Austria):434.
14. Rizzo C, Gesualdo F, Loconsole D, Pandolfi E, Bella A, Orsi A, et al. Moderate vaccine effectiveness against severe acute respiratory infection caused by a(H1N1)pdm09 influenza virus and no effectiveness against a(H3N2)) influenza virus in the 2018/2019 season in Italy. Vaccines. 2020;8(3):1-10.
15. Winokur PL, Hegmann TE, Keitel WA, Bernstein DI, Frey SE, Bryant C. Safety and Immunogenicity of a monovalent inactivated influenza A/H5N8 virus vaccine given with and without AS03 or MF59 adjuvants in healthy adults. Clinical infectious diseases: an official publication of the Infectious Diseases Society of America. 2023((Winokur, Hegmann) Division of Infectious Diseases, Department of Internal Medicine, University of Iowa, Iowa City, IA, United States (Keitel) Departments of Molecular Virology & Microbiology and Medicine, Baylor College of Medicine, Houston, TX, United St).
16. Zimmerman RK, Patricia Nowalk M, Dauer K, Clarke L, Raviotta JM, Balasubramani GK. Vaccine effectiveness of recombinant and standard dose influenza vaccines against influenza related hospitalization using a retrospective test-negative design. Vaccine. 2023.

**Duplicate (n=5)**

1. Colmegna I, Useche ML, Rodriguez K, McCormack D, Alfonso G, Patel A, et al. Immunogenicity and safety of high-dose versus standard-dose inactivated influenza vaccine in rheumatoid arthritis patients: a randomised, double-blind, active-comparator trial. The Lancet Rheumatology. 2020;2(1):e14-e23.
2. Cowling BJ, Perera RAPM, Valkenburg SA, Leung NHL, Iuliano AD, Tam YH, et al. Comparative immunogenicity of several enhanced influenza vaccine options for older adults: A randomized, controlled trial. Clinical Infectious Diseases. 2020;71(7):1704-14.
3. Cowling BJ, Thompson MG, Ng TWY, Fang VJ, Perera RAPM, Leung NHL, et al. Comparative reactogenicity of enhanced influenza vaccines in older adults. Journal of Infectious Diseases. 2020;222(8):1383-91.
4. Pebody R, Whitaker H, Zhao H, Andrews N, Ellis J, Donati M, Zambon M. Protection provided by influenza vaccine against influenza-related hospitalisation in >=65 year olds: Early experience of introduction of a newly licensed adjuvanted vaccine in England in 2018/19. Vaccine. 2020;38(2):173-9.
5. Sanchez L, Matsuoka O, Inoue S, Inoue T, Meng Y, Nakama T, et al. Immunogenicity and safety of high-dose quadrivalent influenza vaccine in Japanese adults >=65 years of age: a randomized controlled clinical trial. Human Vaccines and Immunotherapeutics. 2020;16(4):858-66.

**Non-eligible display of data (n=2)**

1. de Lusignan S, Tsang RSM, Amirthalingam G, Akinyemi O, Sherlock J, Tripathy M, et al. Adverse events of interest following influenza vaccination, a comparison of cell culture-based with egg-based alternatives: English sentinel network annual report paper 2019/20. The Lancet regional health Europe. 2021;2(101777707):100029.
2. Stapleton JT, Wagner N, Tuetken R, Bellamy AR, Hill H, Kim S, Winokur PL. High dose trivalent influenza vaccine compared to standard dose vaccine in patients with rheumatoid arthritis receiving TNF-alpha inhibitor therapy and healthy controls: Results of the DMID 10-0076 randomized clinical trial. Vaccine. 2020;38(23):3934-41.

**Abstract meanwhile published as manuscript (n=1)**

1. Liu C, Schmader K, Harrington T, Roundtree W, Auerbach H, Walter E, et al. Comparative safety of adjuvanted versus high-dose inactivated influenza vaccines in older adults. Journal of the American Geriatrics Society. 2020;68(SUPPL 1):S140.

**Non-eligible publication type (n=1)**

1. Fischer L, O'Brien D, Vasey J, Sylvester GC, Mansi JA. Relative effectiveness of AIIV3 versus IIV4 and HD-IIV3 in preventing influenza-related medical encounters in adults >=65 years of age at high risk for influenza complications during the U.S. 2017-2018 and 2018-2019 influenza seasons. Open Forum Infectious Diseases. 2020;7(SUPPL 1):S844.

# Appendix C - Risk of Bias in included studies

**Efficacy/effectiveness studies**

*Outcome:* ***Laboratory confirmed influenza***

Suppl. Figure 1: Risk of bias VE-studies (assessed with ROBINS-I); outcome: laboratory confirmed influenza


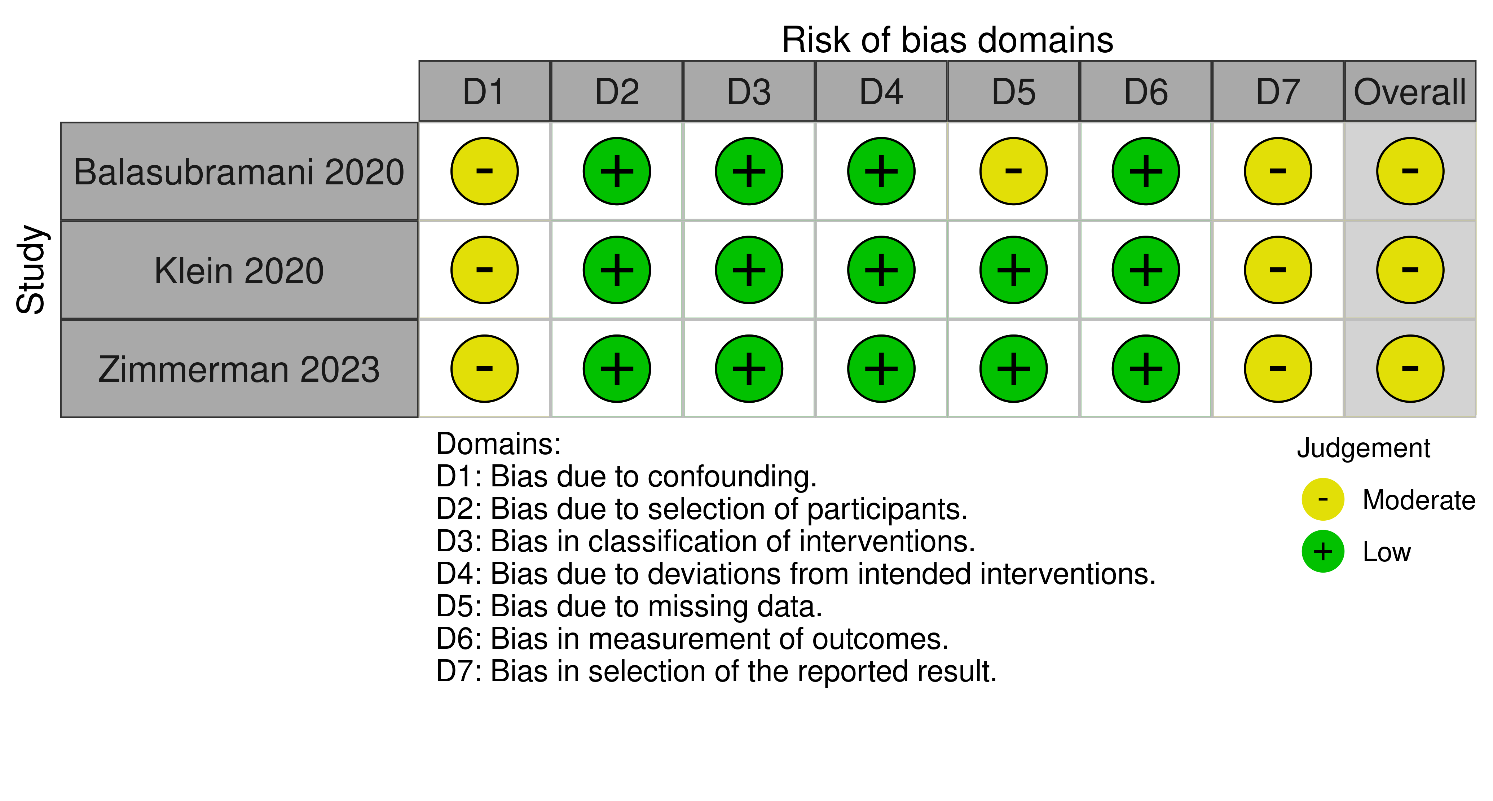


*Outcome:* ***Laboratory confirmed hospitalisation***

Suppl. Figure 2: Risk of bias in VE-studies (assessed with ROBINS-I); outcome: laboratory confirmed hospitalisation


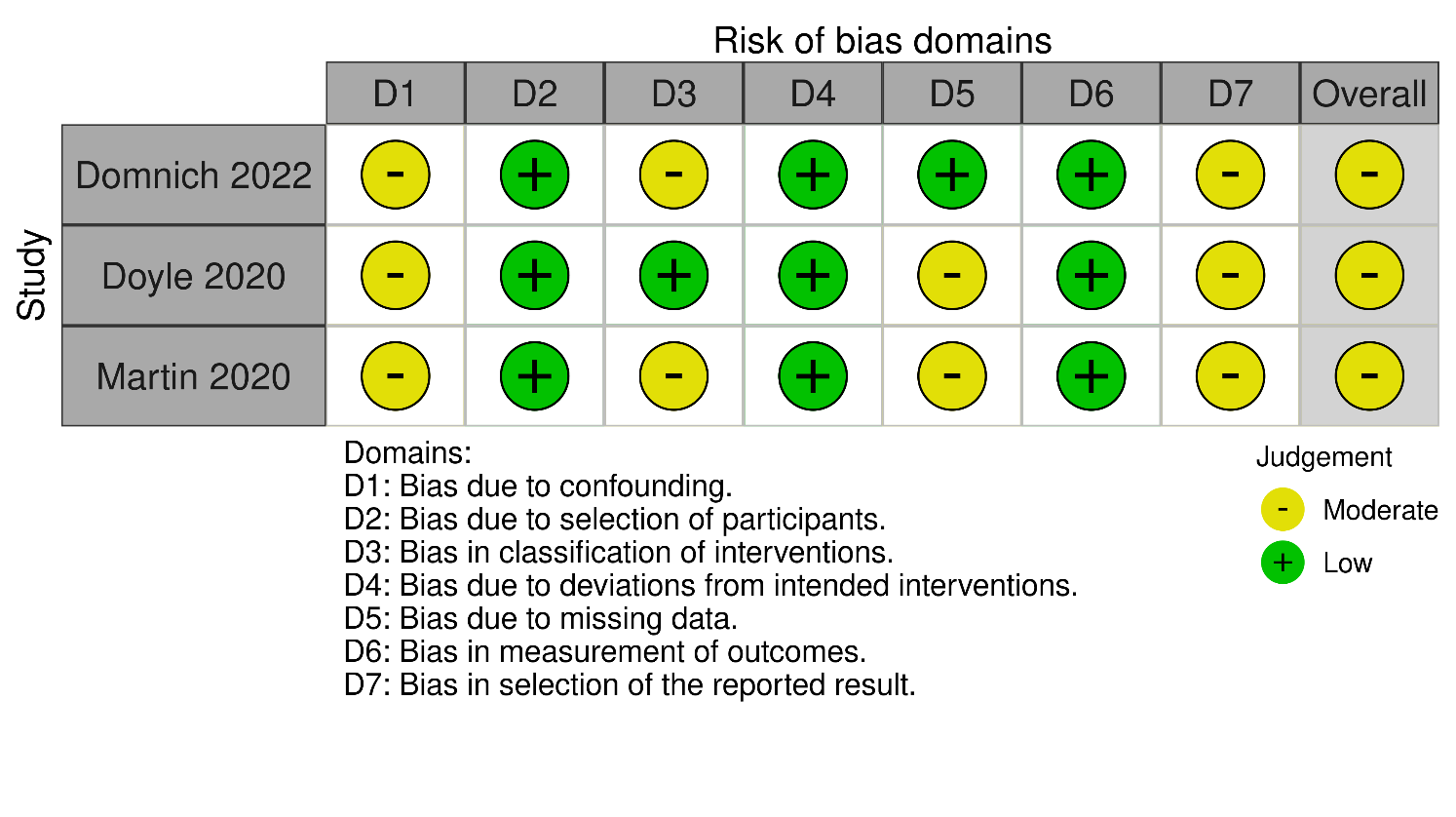


**Safety studies (main outcomes)**

*Outcome:* ***SAE***

Suppl. Figure 3: Risk of bias in NRSI on safety (assessed with ROBINS-I); outcome: SAE (serious adverse events)

*
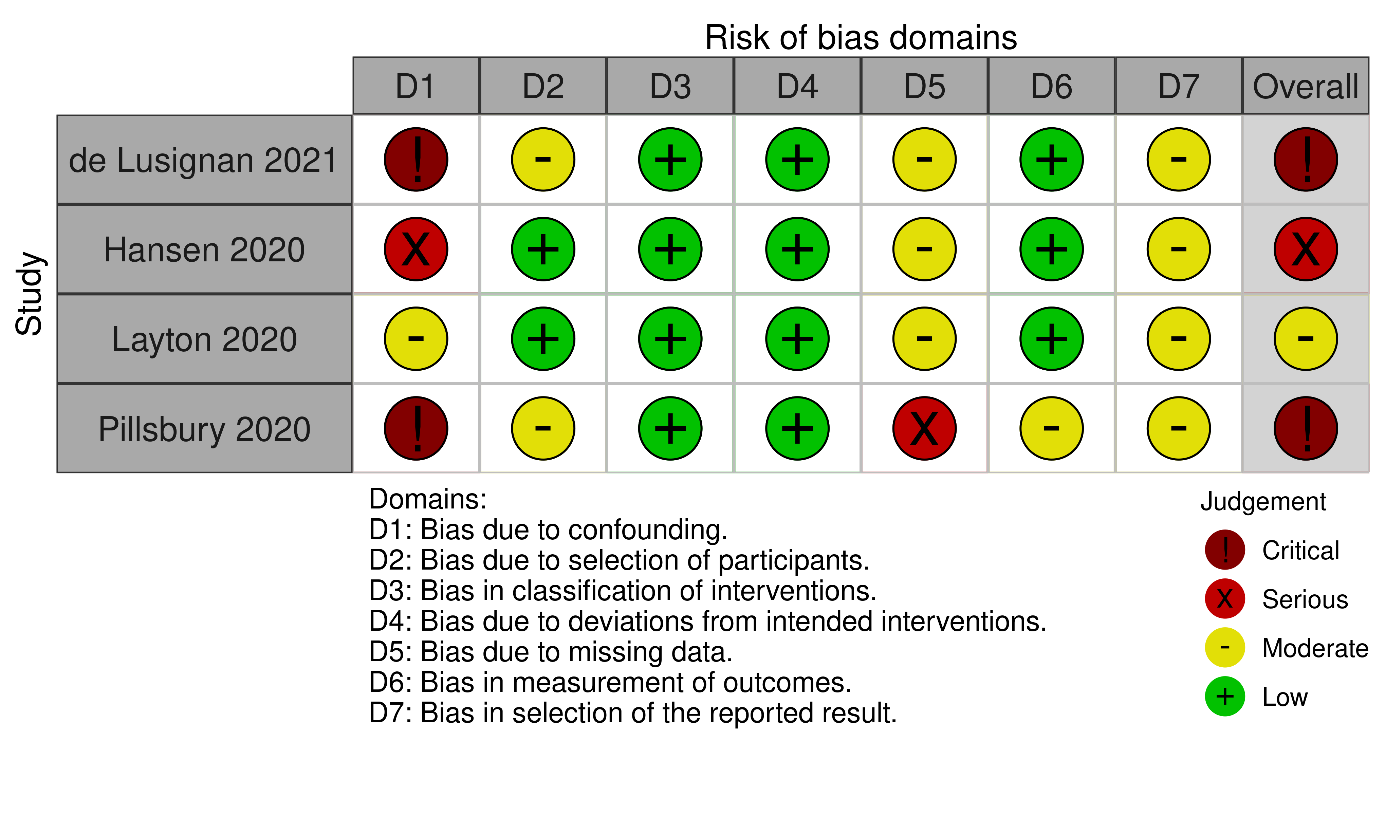
*

*Outcome:* ***Pain***

Suppl. Figure 4: Risk of bias in NRSI on safety (assessed with ROBINS-I); outcome: Pain

*
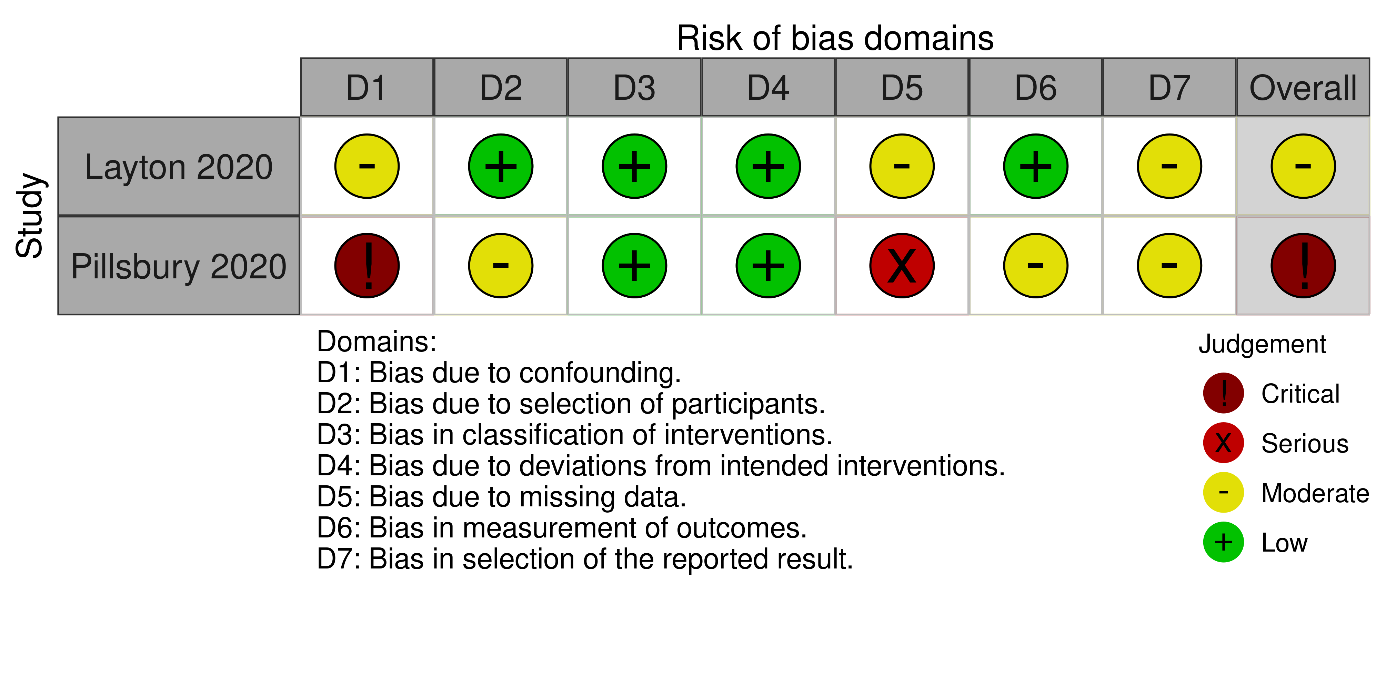
*

*Outcome:* ***Swelling***

Suppl. Figure 5: Risk of bias in NRSI on safety (assessed with ROBINS-I); outcome: Swelling

*
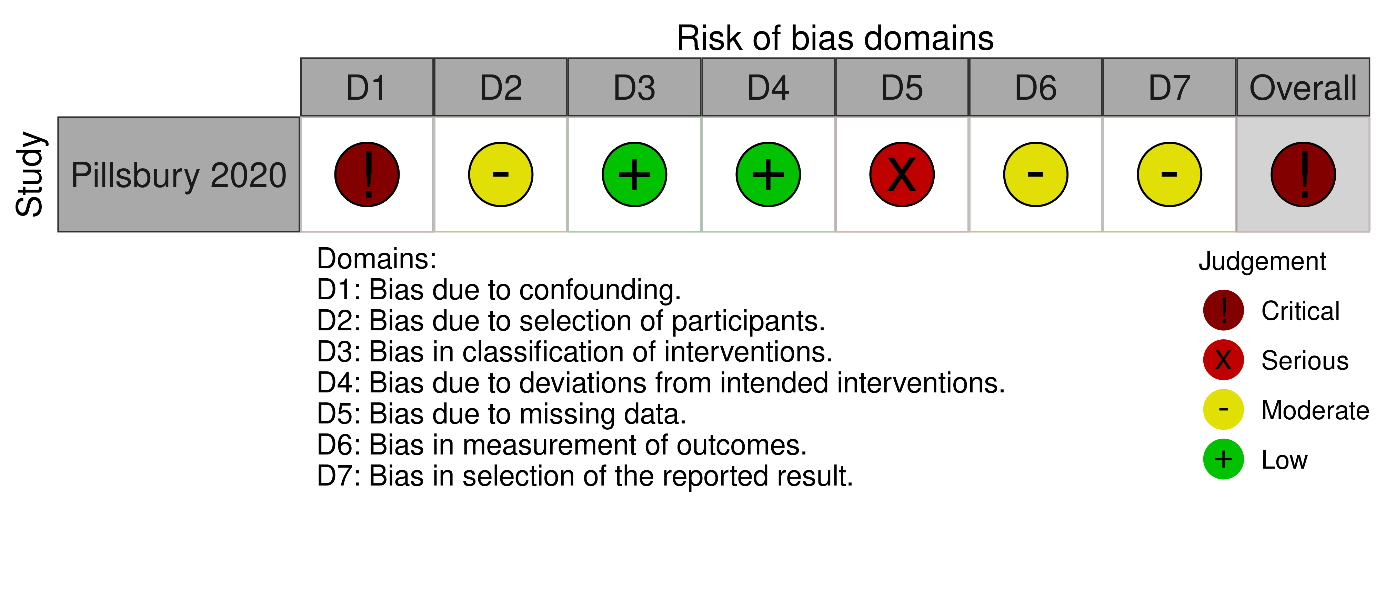
*

*Outcome:* ***Headache***

Suppl. Figure 6: Risk of bias in NRSI on safety (assessed with ROBINS-I); outcome: Headache

*
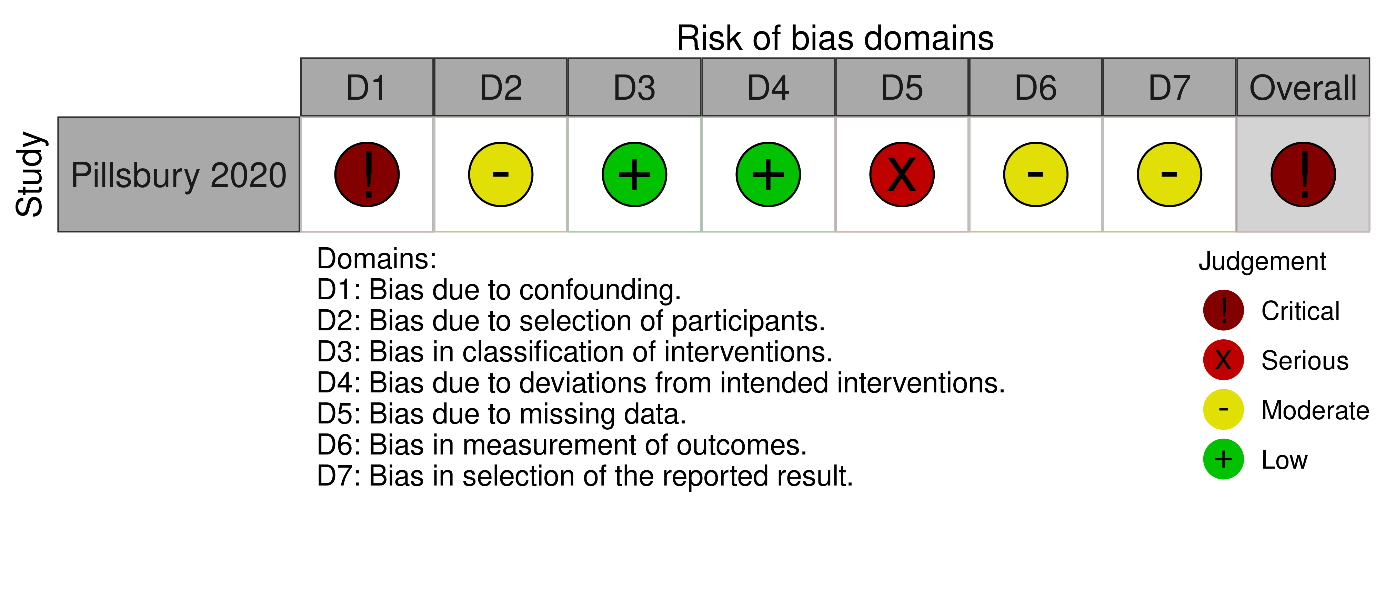
*

*Outcome:* ***Fever***

Suppl. Figure 7: Risk of bias in NRSI on safety (assessed with ROBINS-I); outcome: Fever

*
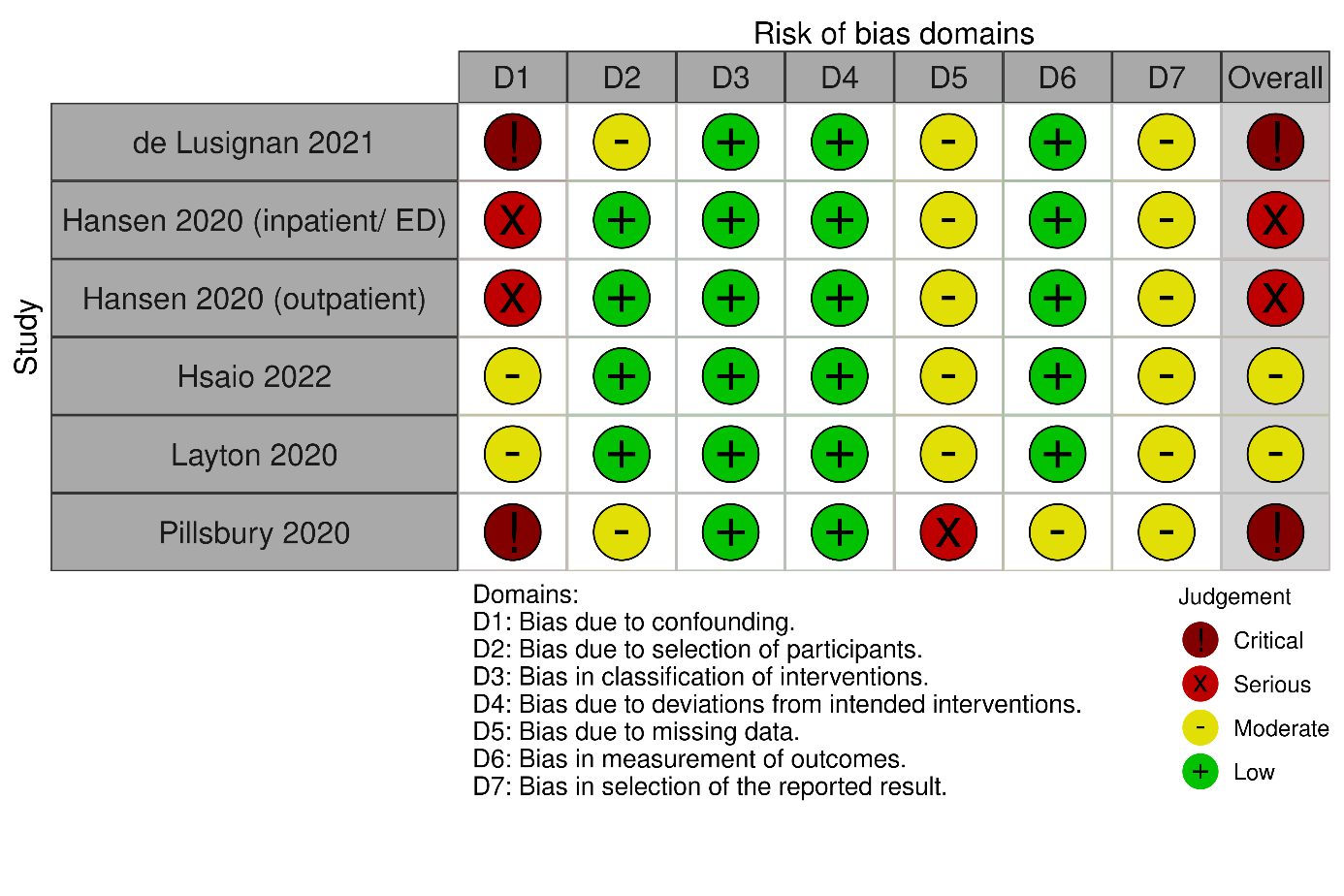
*

***Additional outcomes in NRSI safety-studies:***

Suppl. Figure 8: Risk of bias in NRSI on safety (assessed with ROBINS-I); outcome: anaphylaxis


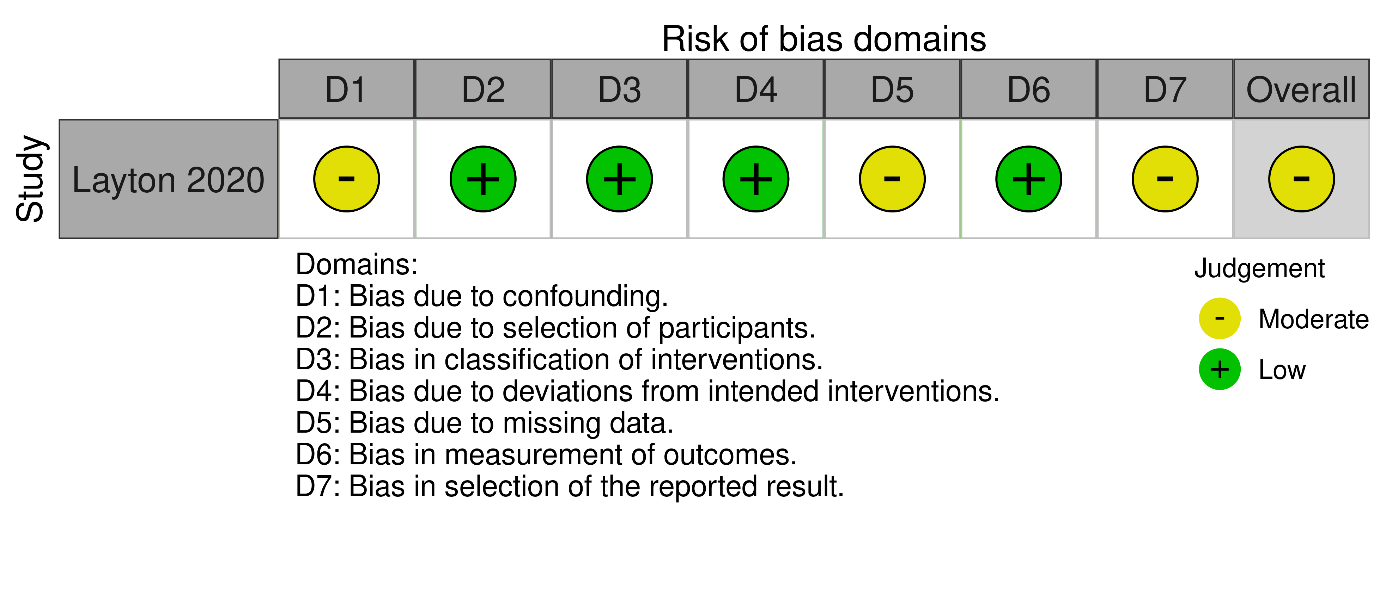


Suppl. Figure 9: Risk of bias in NRSI on safety (assessed with ROBINS-I); outcome: angioedema


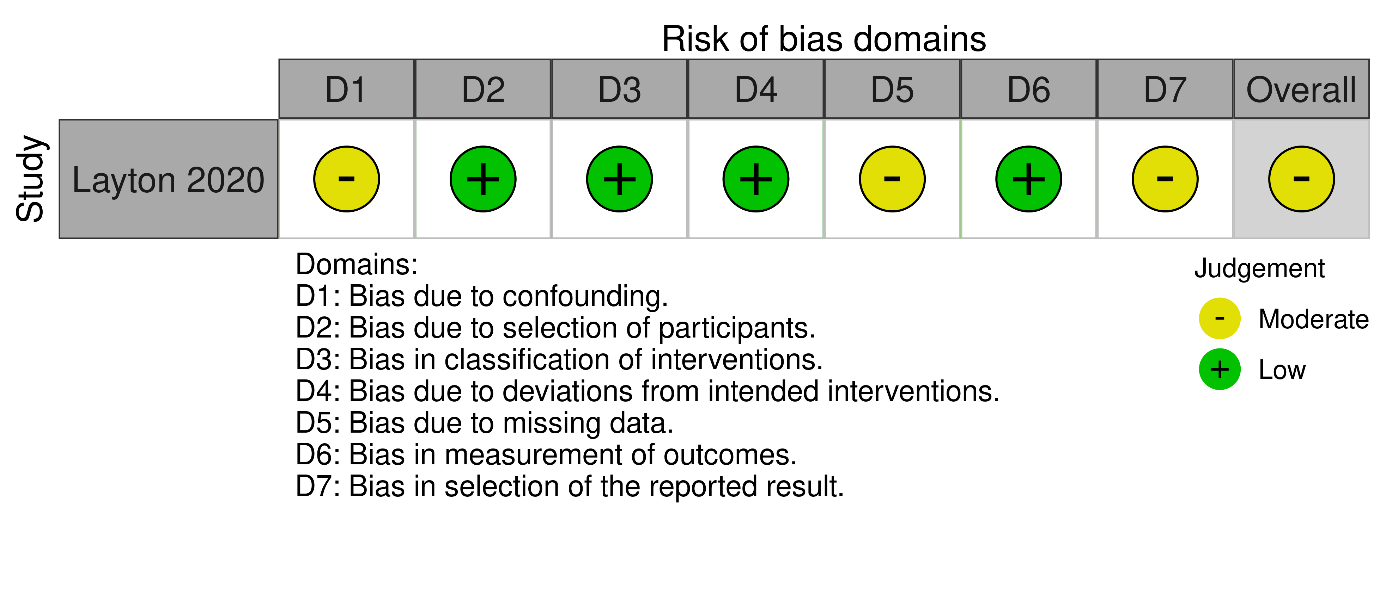


Suppl. Figure 10: Risk of bias in NRSI on safety (assessed with ROBINS-I); outcome: asthma


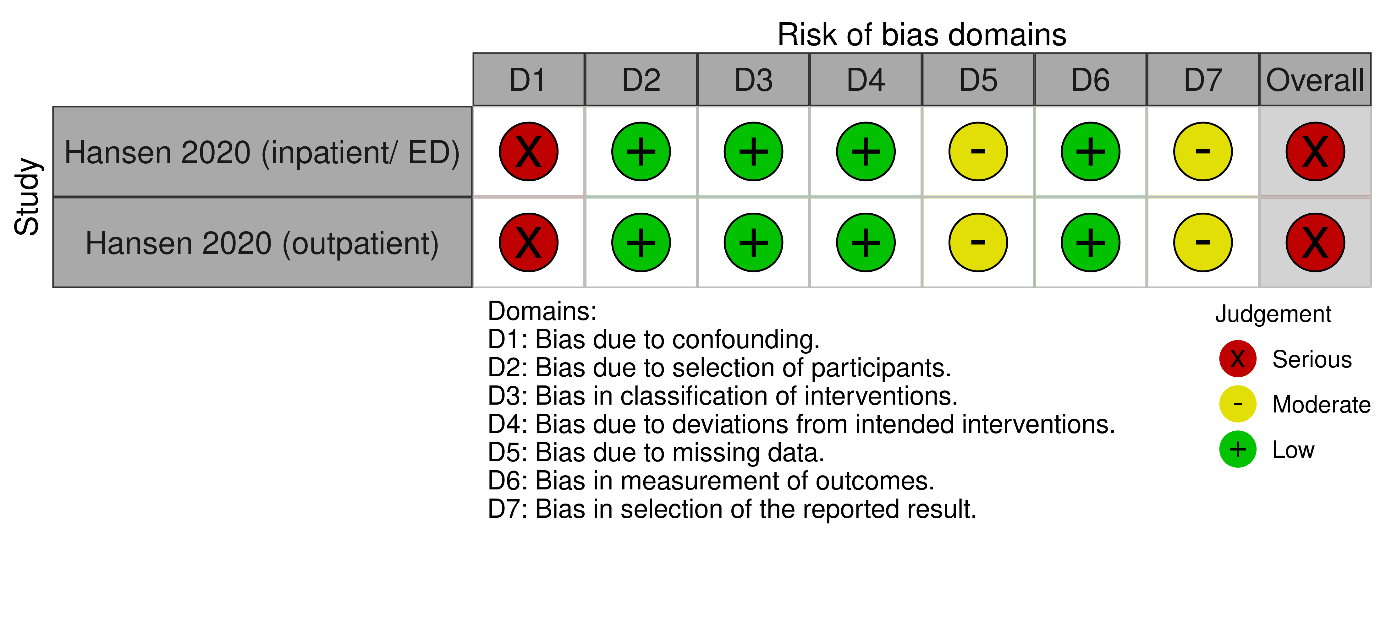


Suppl. Figure 11: Risk of bias in NRSI on safety (assessed with ROBINS-I); outcome: death


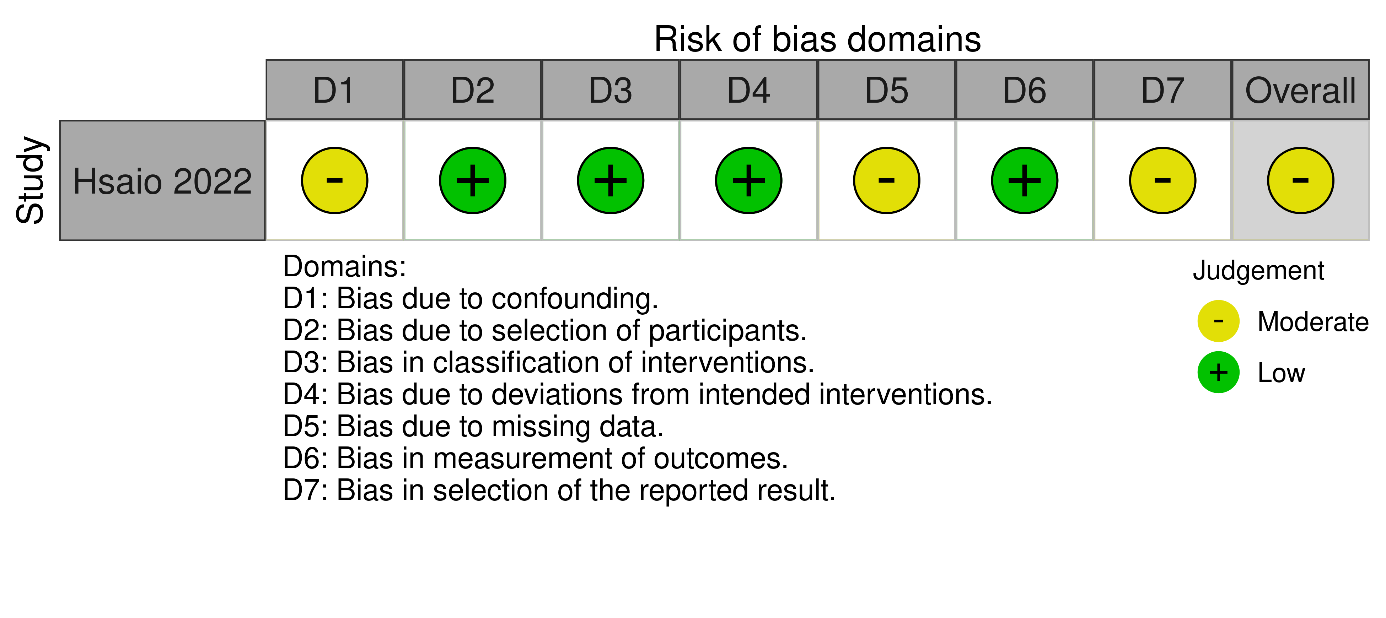


Suppl. Figure 12: Risk of bias in NRSI on safety (assessed with ROBINS-I); outcome: encephalopathy


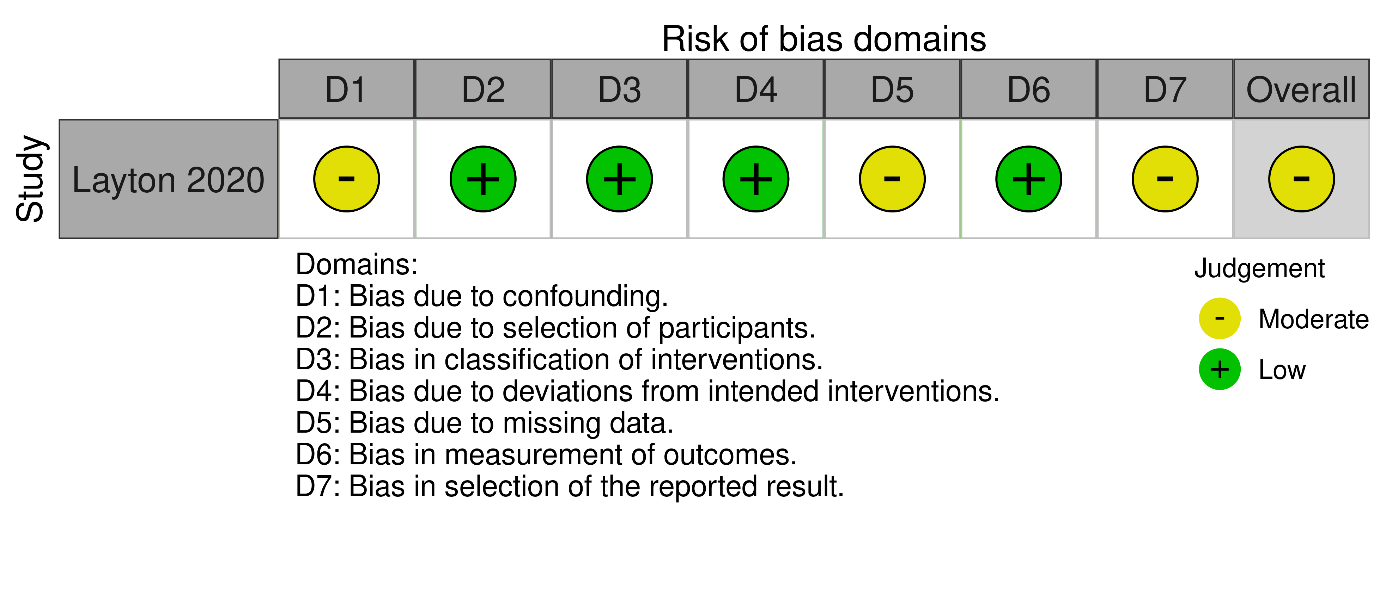


Suppl. Figure 13: Risk of bias in NRSI on safety (assessed with ROBINS-I); outcome: idiopathic thrombocytopenic purpura/ Henoch-Schönlein purpura


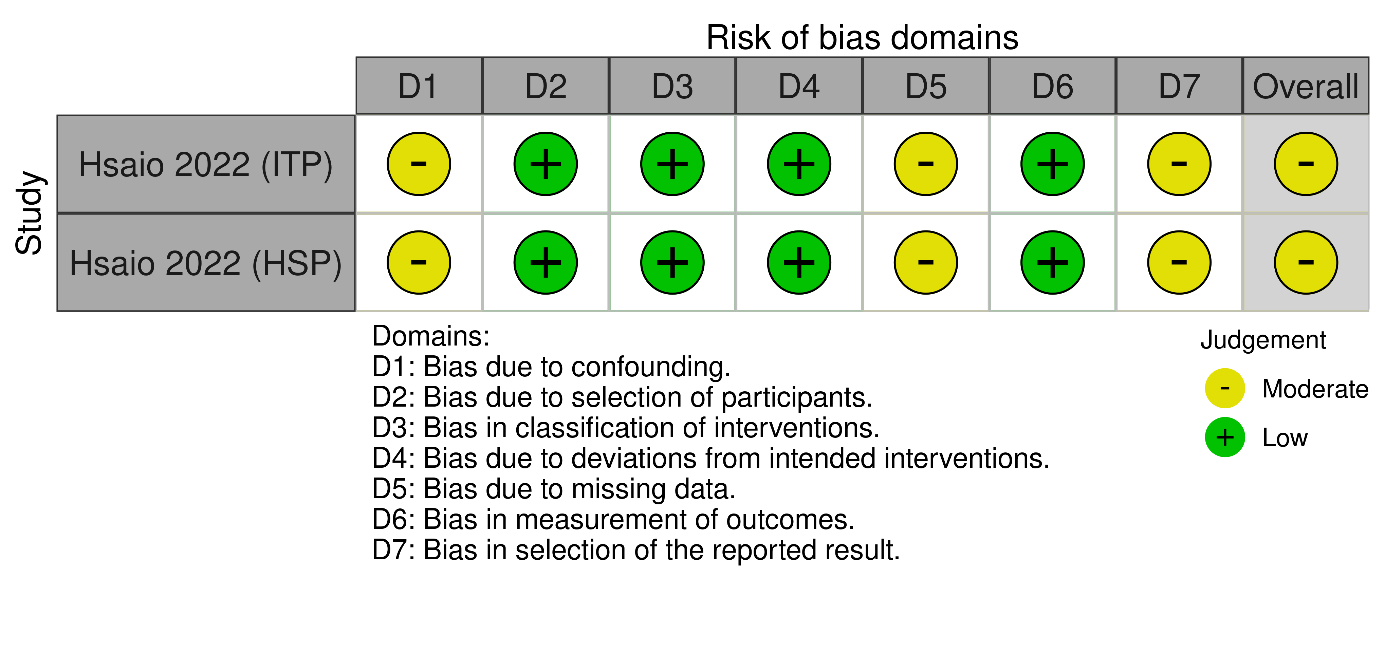


Suppl. Figure 14: Risk of bias in NRSI on safety (assessed with ROBINS-I); outcome: narcolepsy/cataplexy


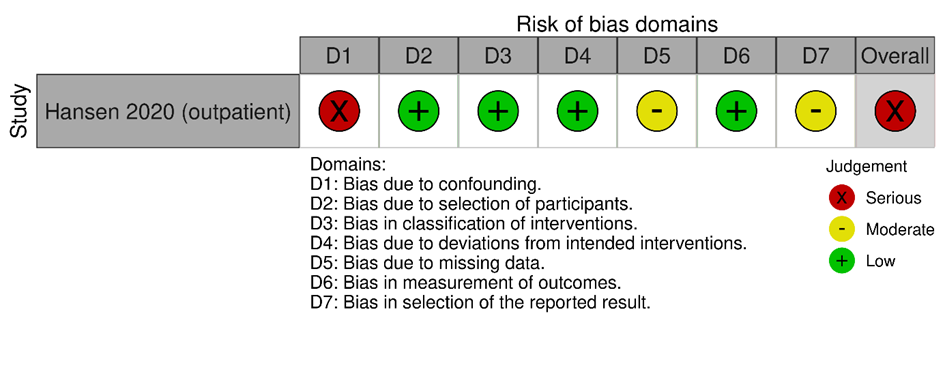


Suppl. Figure 15: Risk of bias in NRSI on safety (assessed with ROBINS-I); outcome: non-infectious pleural effusion


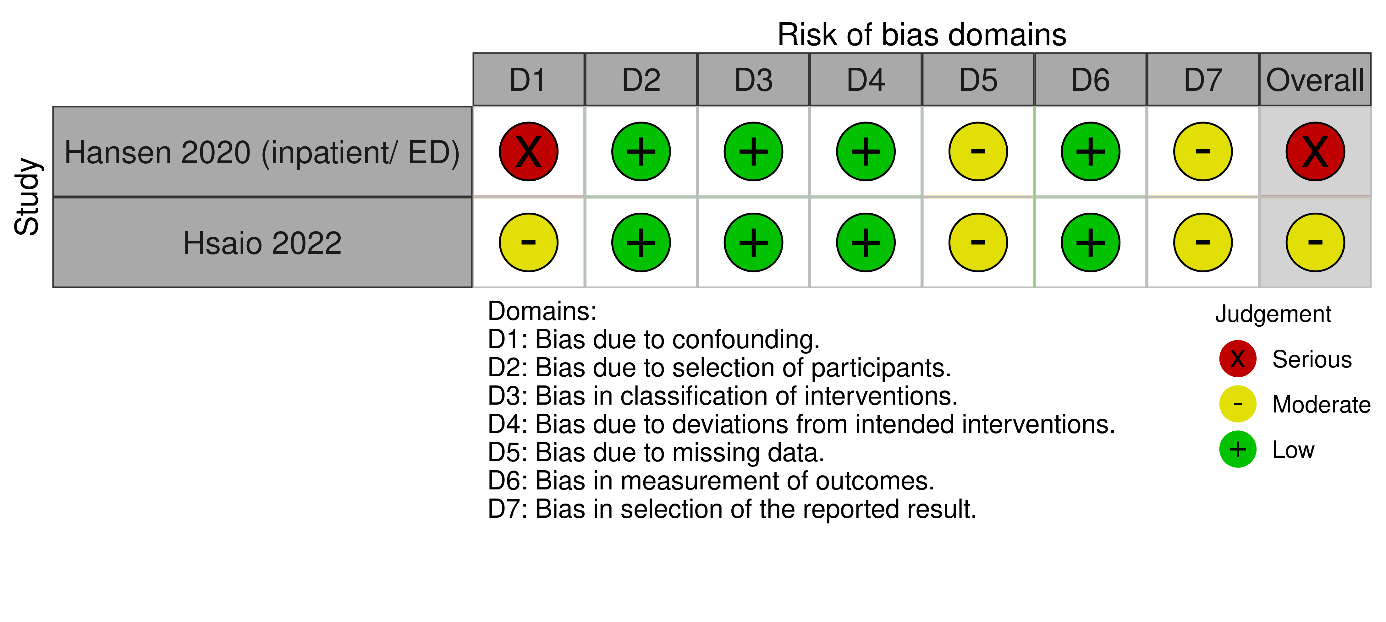


Suppl. Figure 16: Risk of bias in NRSI on safety (assessed with ROBINS-I); outcome: acute non-infectious pericarditis


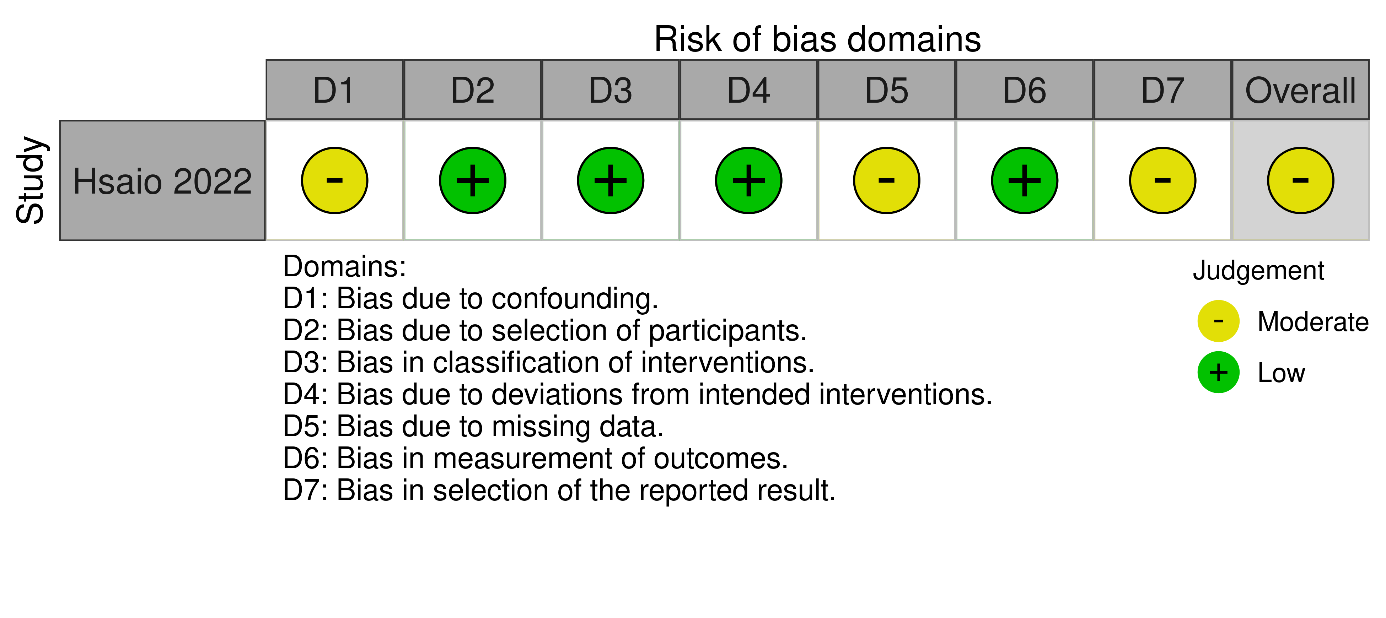


Suppl. Figure 17: Risk of bias in NRSI on safety (assessed with ROBINS-I); outcome: seizure/ convulsion


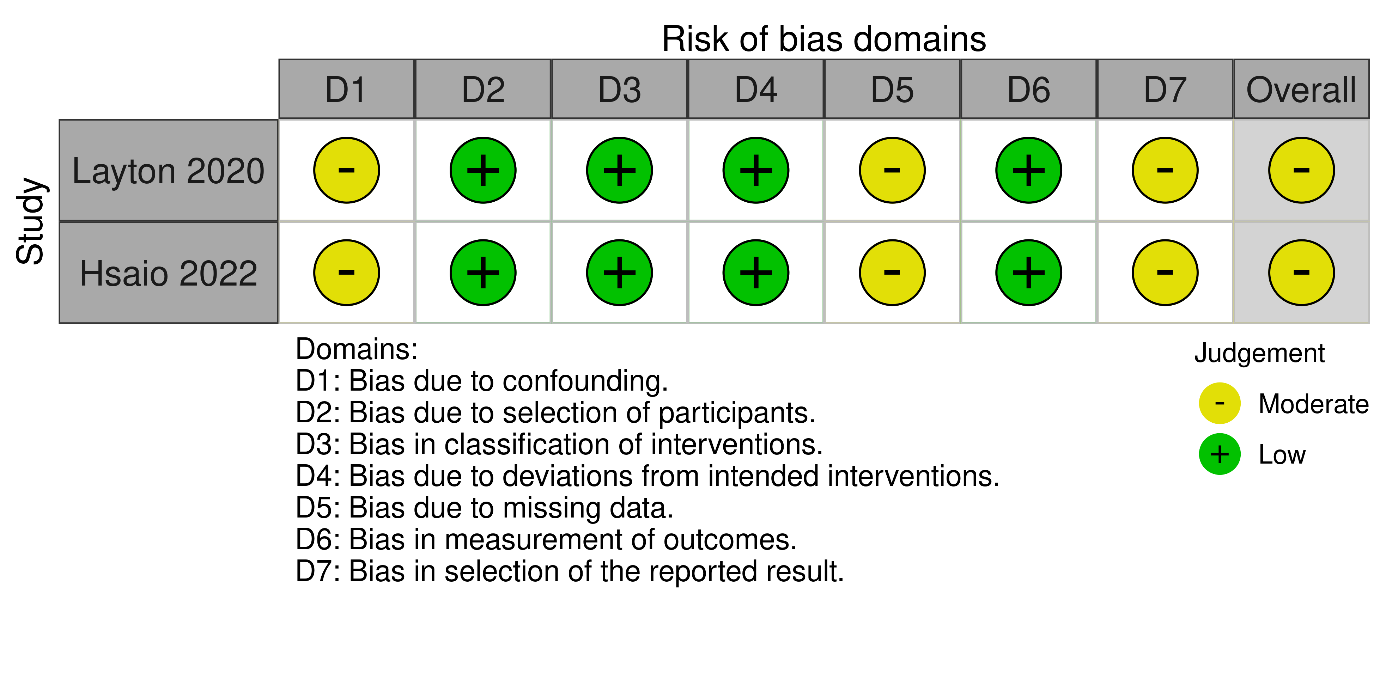


Suppl. Figure 18: Risk of bias in NRSI on safety (assessed with ROBINS-I); outcome: short-term mortality


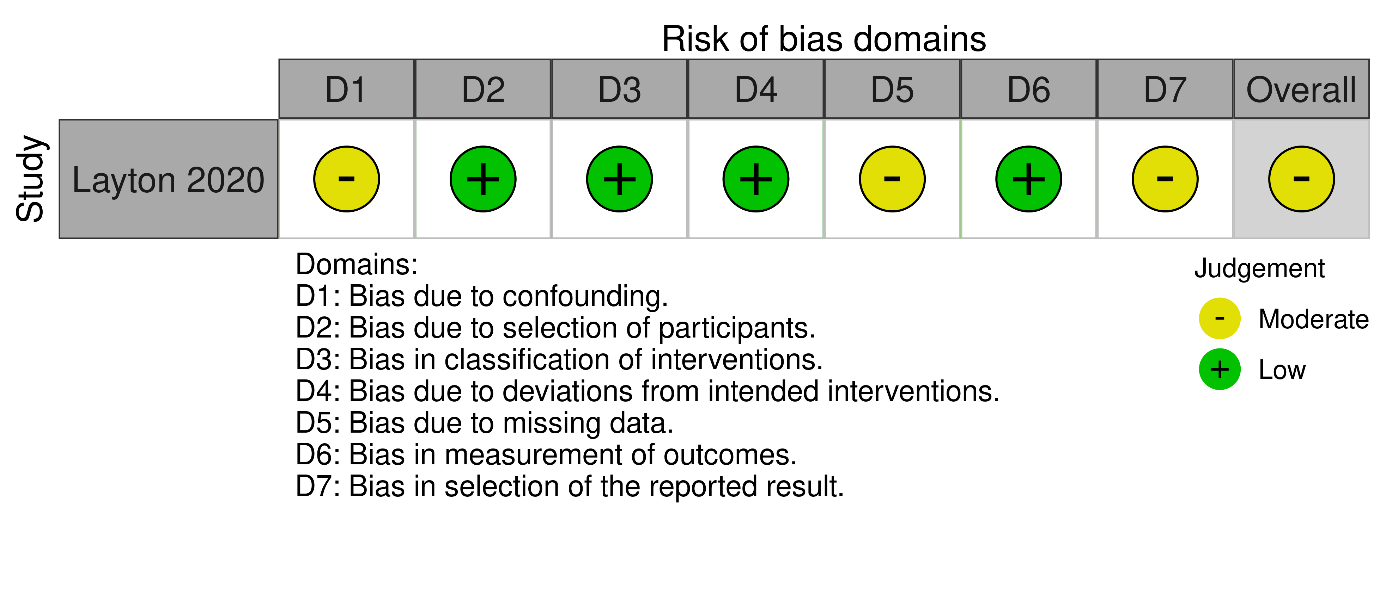


***Additional outcomes in RCT safety-studies:***

Suppl. Figure 19: Risk of bias in RCT safety-studies (assessed with RoB 2); outcome: AESI


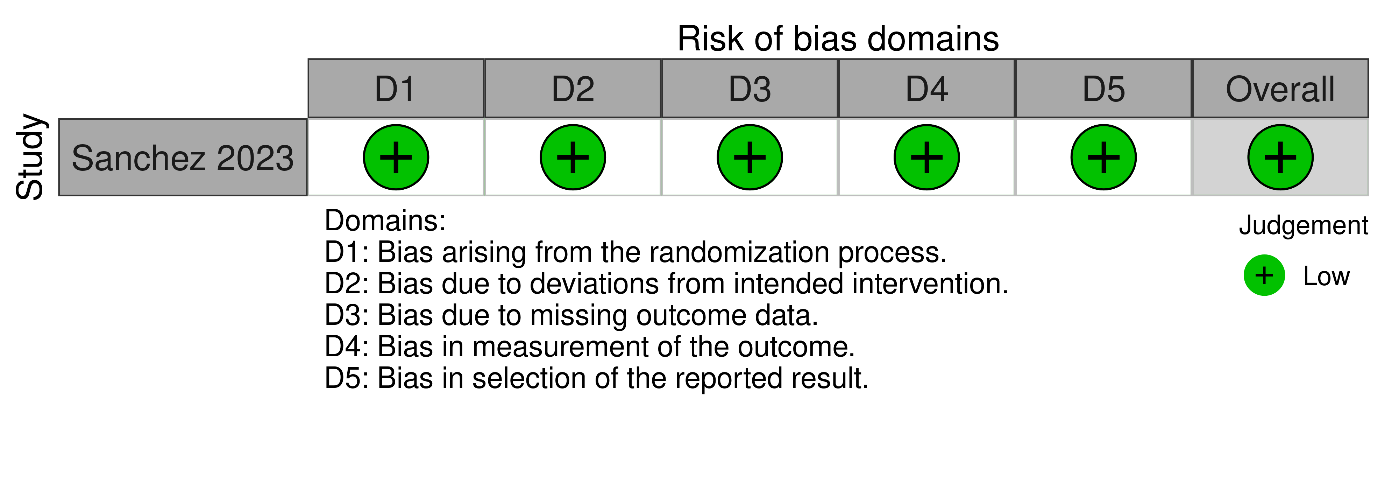


Suppl. Figure 20: Risk of bias in RCT safety-studies (assessed with RoB 2); outcome: death


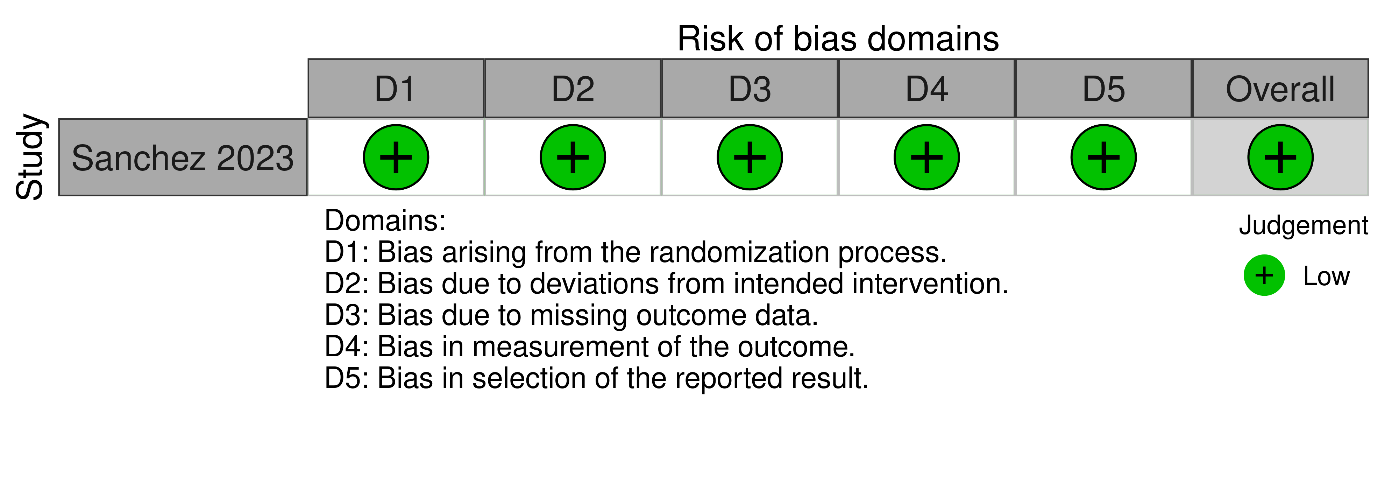


# Appendix D - Funnel Plots

For comparisons of outcomes with 10 or more studies, funnel plots were constructed and visually inspected for small study effects. In none of them evidence for publication bias was detected.

**MF59-adjuvanted influenza vaccine versus standard influenza vaccine**

**
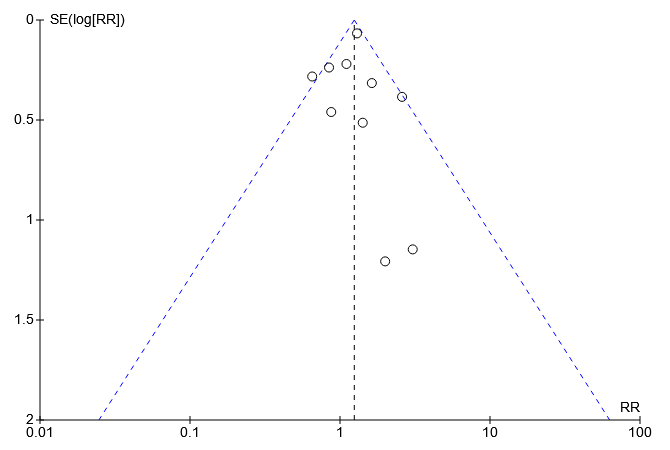
**

Suppl. Figure 21: Funnel plot for safety outcome headache after vaccination with MF59-adjuvanted influenza vaccine versus standard influenza vaccine (10 studies)


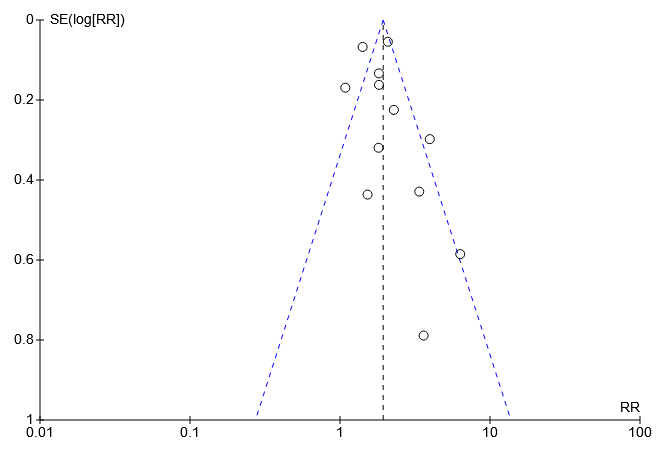


Suppl. Figure 22: Funnel plot for safety outcome pain after vaccination with MF59-adjuvanted influenza vaccine versus standard influenza vaccine (12 studies)

**High-dose influenza vaccine versus standard influenza vaccine**
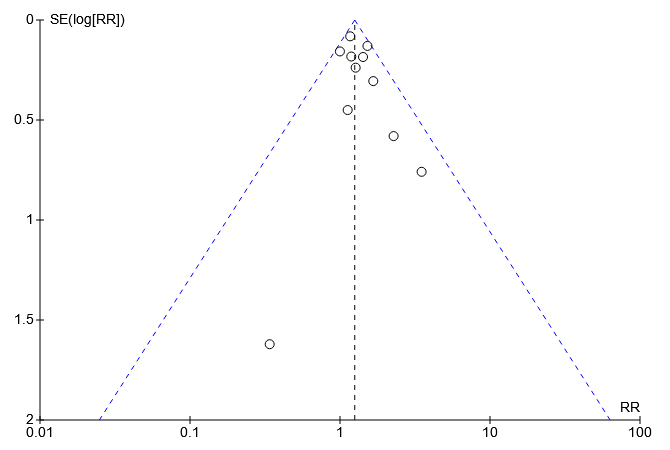


Suppl. Figure 23: Funnel plot for safety outcome headache after vaccination with high-dose influenza vaccine versus standard influenza vaccine (11 studies)


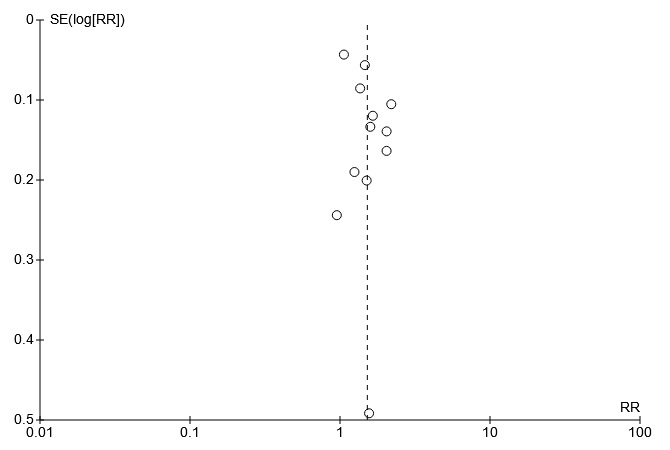


Suppl. Figure 24: Funnel plot for safety outcome pain after vaccination with high-dose influenza vaccine versus standard influenza vaccine (12 studies)

# Appendix E - Local and systemic adverse events

### **MF59-adjuvanted influenza vaccine**

*Secondary safety outcomes*

Systemic adverse events

In the basic review, 10 RCTs were included which reported on headache after vaccination. The pooled risk ratio was 1.25 (95%CI: 1.11 to 1.39) in the fixed-effects model and 1.18 (95%CI: 0.94 to 1.48) according to the random effects model (Suppl. Figure 25-26). In the update, no additional studies were identified.
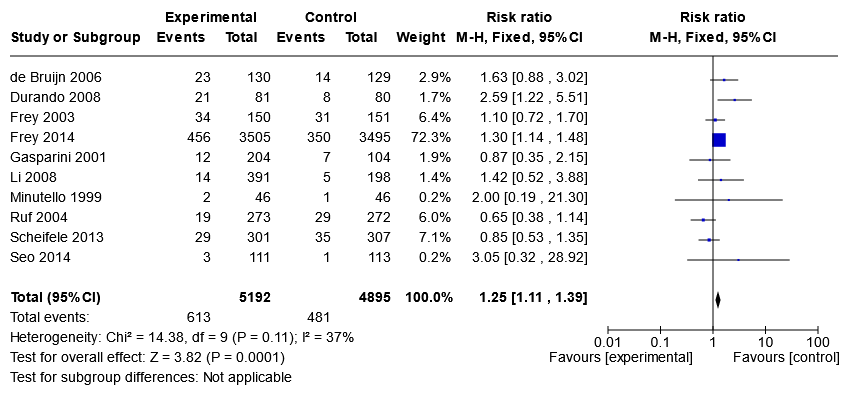


Suppl. Figure 25: Relative risk of headache after vaccination with MF59-adjuvanted influenza vaccine versus standard influenza vaccine (fixed-effects model)


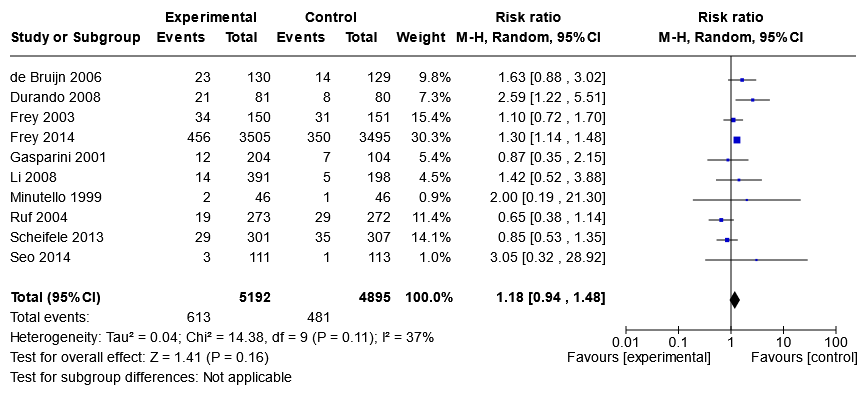


Suppl. Figure 26: Relative risk of headache after vaccination with MF59-adjuvanted influenza vaccine versus standard influenza vaccine (random-effects model)

9 RCTs were included in the primary review which reported on fever after vaccination. The pooled risk ratio was 1.83 (95%CI: 1.49 to 2.23) in the fixed effects model and 1.95 (95%CI: 1.35 to 2.80) according to the random effects model (Suppl. Figure 27-28). In the update, no additional studies were identified.
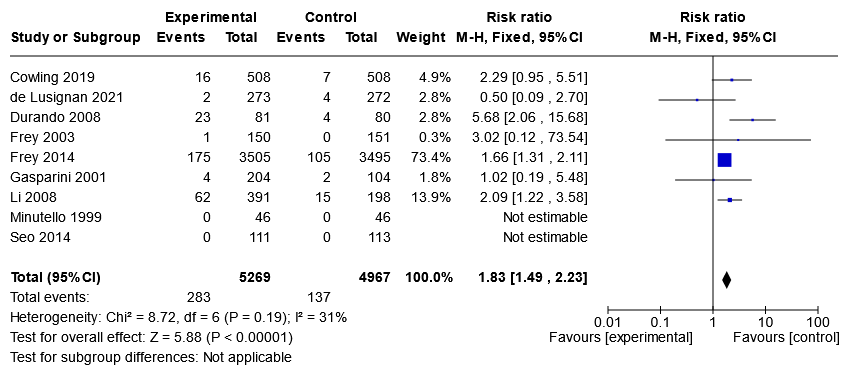


Suppl. Figure 27: Relative risk of fever after vaccination with MF59-adjuvanted influenza vaccine versus standard influenza vaccine (fixed-effects model)


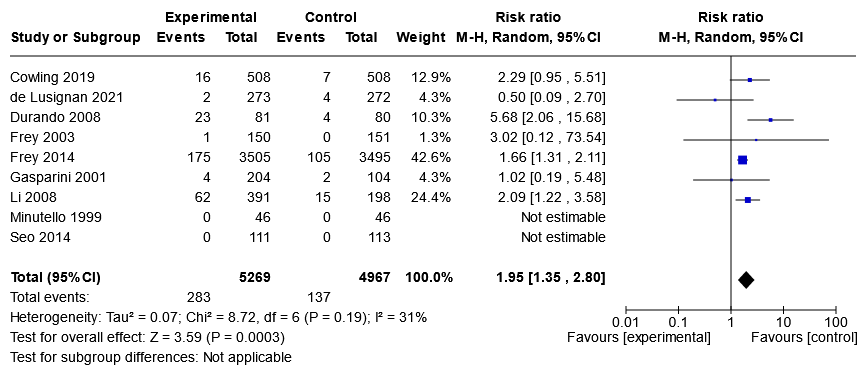


Suppl. Figure 28: Relative risk of fever after vaccination with MF59-adjuvanted influenza vaccine versus standard influenza vaccine (random-effects model)

Local adverse events

In the primary review, 12 RCTs reported on pain at the injection site after vaccination. The pooled risk ratio of the fixed effects model was 1.94 (95%CI: 1.80 to 2.10) and 1.94 (95%CI: 1.58 to 2.40) according to the random effects model (Suppl. Figure 29-30). In the update, no additional studies were identified.


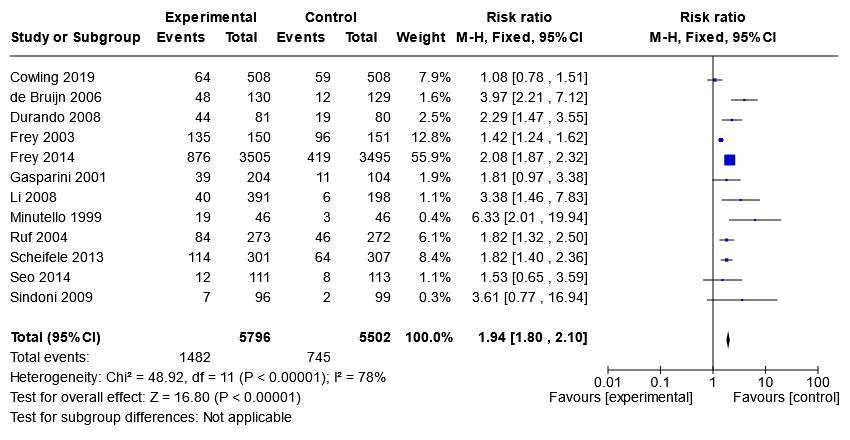


Suppl. Figure 29: Relative risk of pain after vaccination with MF59-adjuvanted influenza vaccine versus standard influenza vaccine (fixed-effects model)


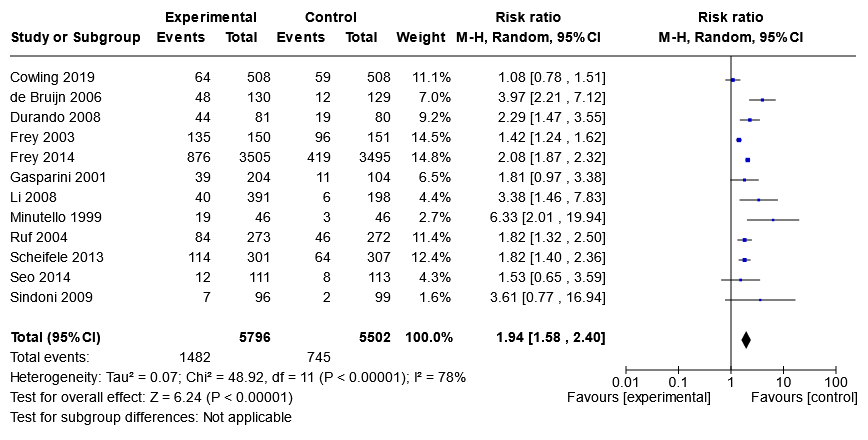


Suppl. Figure 30: Relative risk of pain after vaccination with MF59-adjuvanted influenza vaccine versus standard influenza vaccine (random-effects model)

Swelling at the injection site was reported in 5 RCTs included in the primary review. The pooled risk ratio was 1.24 (95%CI: 0.97 to 1.60) in the fixed effects model and 1.26 (95%CI: 0.91 to 1.74) according to the random effects model (Suppl. Figure 31-32). In the update, no additional studies were identified.


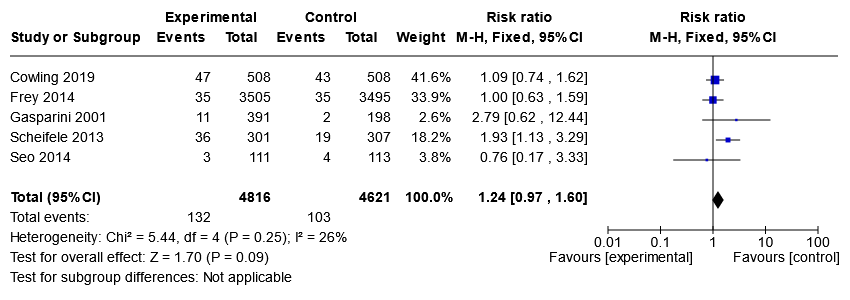


Suppl. Figure 31: Relative risk of swelling after vaccination with MF59-adjuvanted influenza vaccine versus standard influenza vaccine (fixed-effects model)


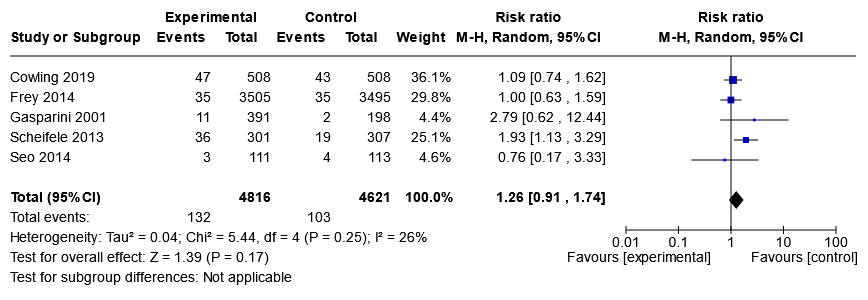


Suppl. Figure 32: Relative risk of swelling after vaccination with MF59-adjuvanted influenza vaccine versus standard influenza vaccine (random-effects model)

Adverse pregnancy outcomes after vaccination during pregnancy

No studies reported on this outcome, neither in the primary review nor in the update.

Adverse neonatal outcomes after vaccination during pregnancy

No studies reported on this outcome, neither in the primary review nor in the update.

### **High-dose influenza vaccine**

*Secondary safety outcomes*

Systemic adverse events

For headache, the primary review included data from 7 RCTs that resulted in a pooled RR of 1.24 (95%CI: 1.09 to 1.40; fixed effects model; random-effects model: 1.36; 95%CI: 1.02 to 1.77). In the update, we identified three additional RCTs (34, 35, 37) which provided 4 estimates. Adding these data to the evidence base led to an updated pooled RR of 1.25 (95%CI: 1.13 to 1.39; fixed effects model; random effects model: 1.25 (95%CI: 1.13 to 1.40) (Suppl. Figure 33-34).


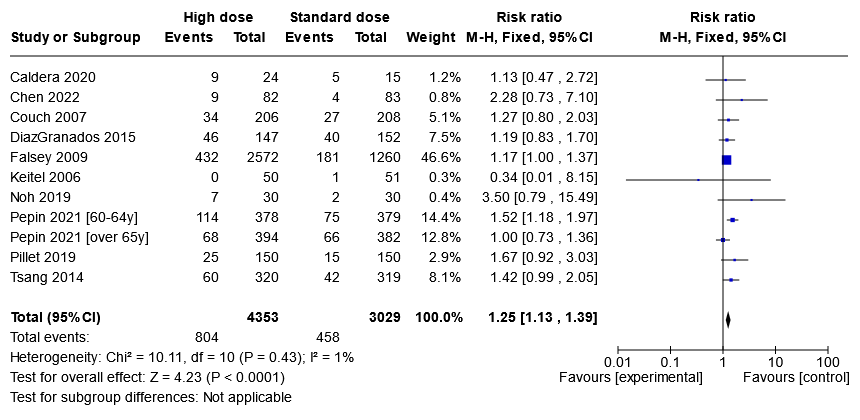


Suppl. Figure 33: Relative risk of headache after vaccination with high-dose influenza vaccine versus standard influenza vaccine (fixed-effects model)


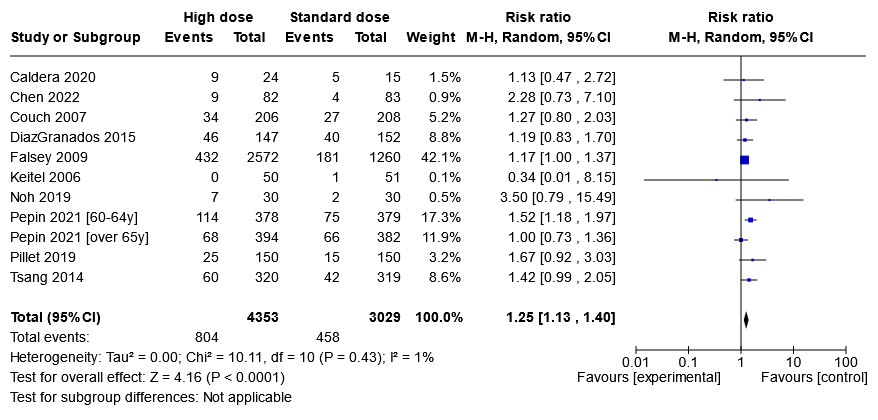


Suppl. Figure 34: Relative risk of headache after vaccination with high-dose influenza vaccine versus standard influenza vaccine (random-effects model)

Fever was reported in 7 RCTs in the primary review. The pooled RR was 1.83 (95%CI: 1.29 to 2.60) by fixed effects model and 2.06 (95%CI: 0.84 to 5.06) by random effects model. In the update, 3 additional studies (2 RCT, 1 NRSI) were found (34-36). Adding the RCT data to the evidence base resulted in an updated pooled RR of 1.85 (95%CI: 1.31 to 2.61; fixed effects model; random effects model: 1.78 (95%CI: 1.25 to 2.54) (Suppl. Figure 35-36). In addition, the NRSI (36) reported a RR of 0.92 (95%CI: 0.78 to 1.08).


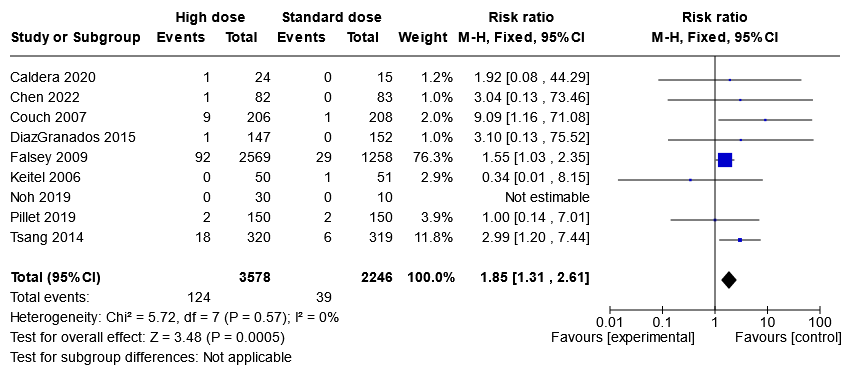


Suppl. Figure 35: Relative risk of fever after vaccination with high-dose influenza vaccine versus standard influenza vaccine (fixed-effects model)


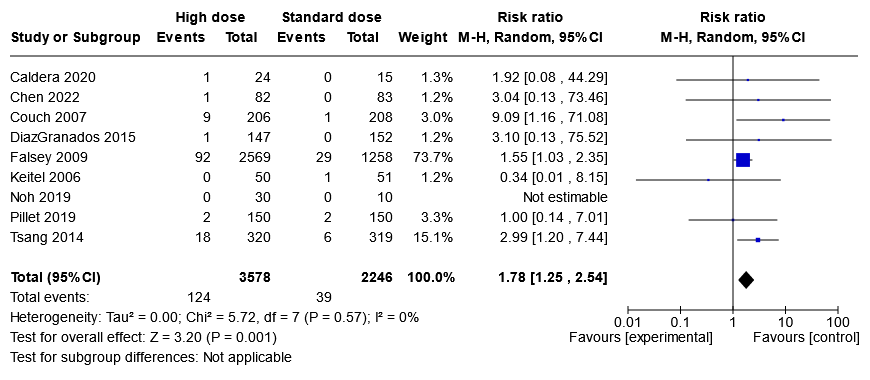


Suppl. Figure 36: Relative risk of fever after vaccination with high-dose influenza vaccine versus standard influenza vaccine (random-effects model)

Local adverse events

Pain at the injection site after vaccination was reported in 7 RCTs in the primary review. Pooled RR after high-dose vaccine, compared to standard vaccine, was 1.55 (95%CI: 1.43 to 1.67) using the fixed effects model and 1.56 (95%CI: 1.26 to 1.93) according to random effects model. The update identified 5 additional studies (4 RCTs, 1 NRSI) reporting 6 estimates. After adding the RCT data to the evidence base, the updated pooled RR was 1.40 (95%CI: 1.33 to 1.48) in the fixed effects model; random effects model: 1.52 (95%CI: 1.29 to 1.80) (Suppl. Figure 37-38). The NRSI (36) reported a RR of 1.23 (95%CI: 1.12 to 1.34).
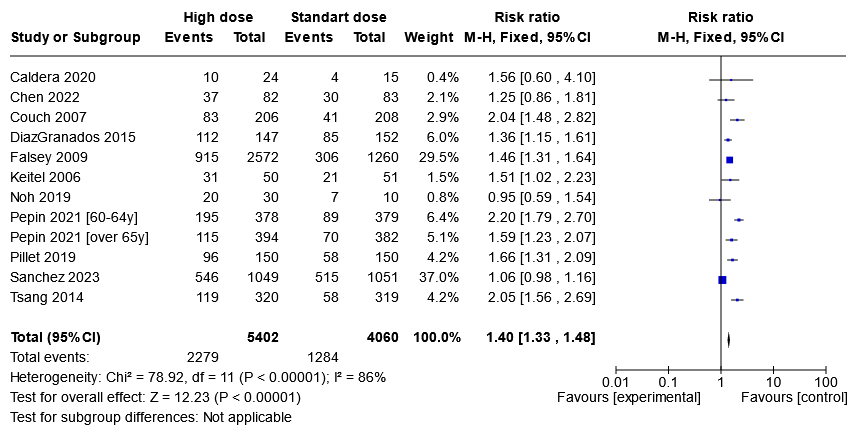


Suppl. Figure 37: Relative risk of pain after vaccination with high-dose influenza vaccine versus standard influenza vaccine (fixed-effects model)


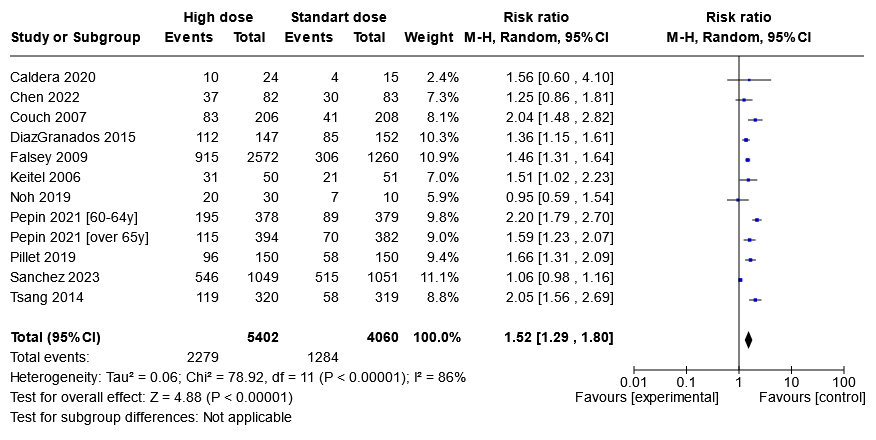


Suppl. Figure 38: Relative risk of pain after vaccination with high-dose influenza vaccine versus standard influenza vaccine (random-effects model)

Injection site swelling after vaccination was reported in 6 RCTs in the primary review. The pooled RR across these studies was 1.84 (95%CI: 1.49 to 2.27) according to fixed effects model and 2.20 (95%CI: 1.12 to 4.32) by random effects model. In the update, 2 additional RCTs were identified (34, 35). Adding their data to the evidence base resulted in an updated pooled RR of 1.81 (95%CI: 1.48 to 2.23; fixed effects model; random effects model: 1.85 (95%CI: 1.27 to 2.71) (Suppl. Figure 39-40).
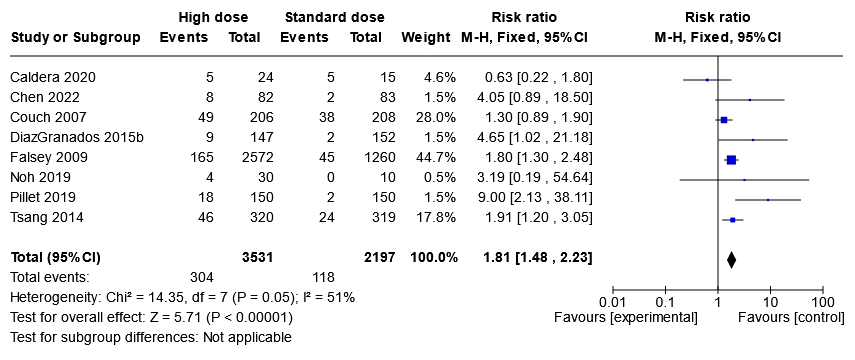


Suppl. Figure 39: Relative risk of swelling after vaccination with high-dose influenza vaccine versus standard influenza vaccine (fixed-effects model)


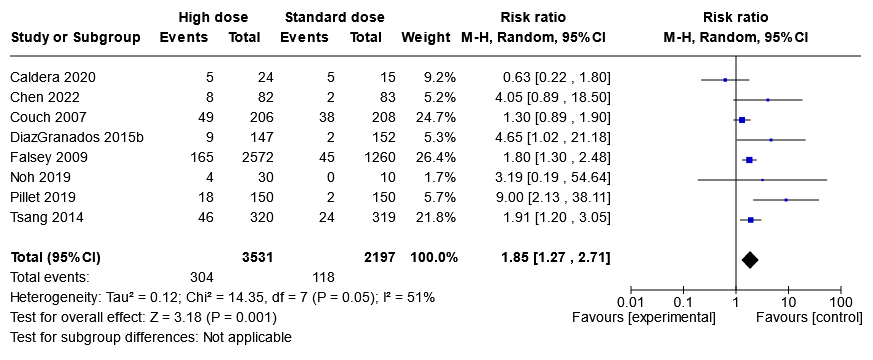


Suppl. Figure 40: Relative risk of swelling after vaccination with high-dose influenza vaccine versus standard influenza vaccine (random-effects model)

Adverse pregnancy outcomes after vaccination during pregnancy

No studies reported on this outcome, neither in the primary review nor in the update.

Adverse neonatal outcomes after vaccination during pregnancy

No studies reported on this outcome, neither in the primary review nor in the update.

### **Cell-based influenza vaccine**

*Secondary safety outcomes*

Systemic adverse events

In the primary review, headache was reported from 6 RCTs. Pooled RR was 1.03 (95%CI: 0.94 to 1.12; fixed effects model; random-effects model: 1.02; 95%CI: 0.94 to 1.11) (Suppl. Figure 41-42). No additional studies were identified in the update.


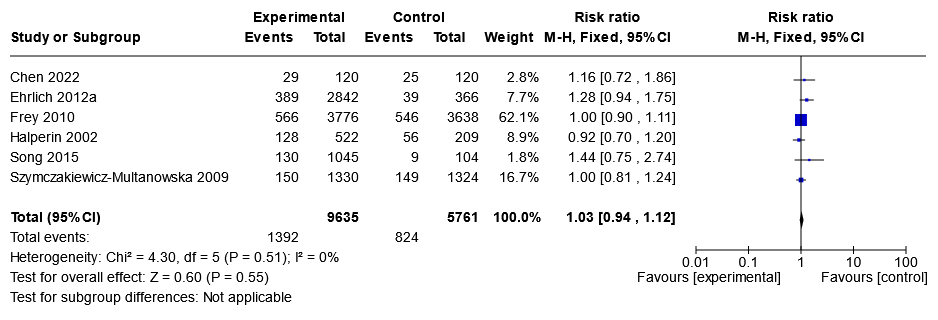


Suppl. Figure 41: Relative risk of headache after vaccination with cell-based influenza vaccine versus standard influenza vaccine (fixed-effects model)


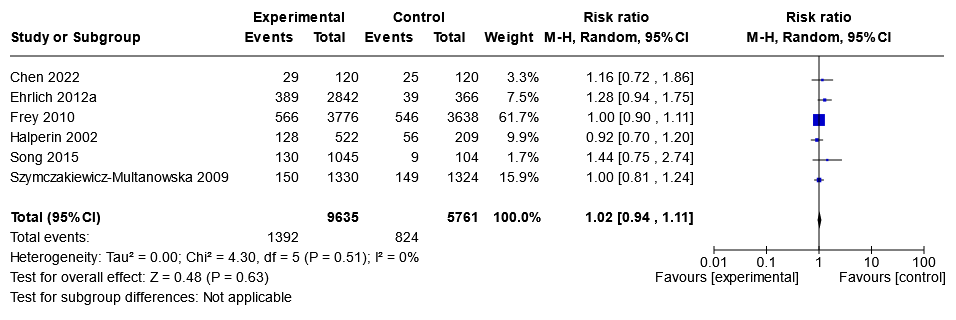


Suppl. Figure 42: Relative risk of headache after vaccination with cell-based influenza vaccine versus standard influenza vaccine (random-effects model)

Six RCTs were identified in the primary review which provided data on fever after vaccination. Using a fixed effects model, the pooled RR was 1.05 (95%CI: 0.73 to 1.52); by random effects model, pooled RR was 1.00 (95%CI: 0.69 to 1.45) (Suppl. Figure 43-44). No additional studies were identified in the update.
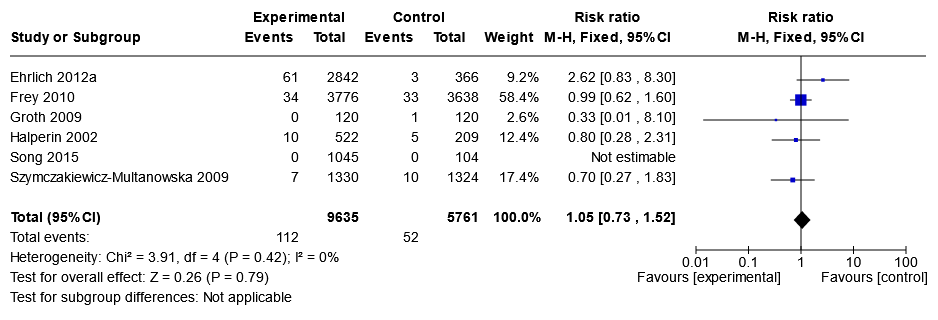


Suppl. Figure 43: Relative risk of fever after vaccination with cell-based influenza vaccine versus standard influenza vaccine (fixed-effects model)


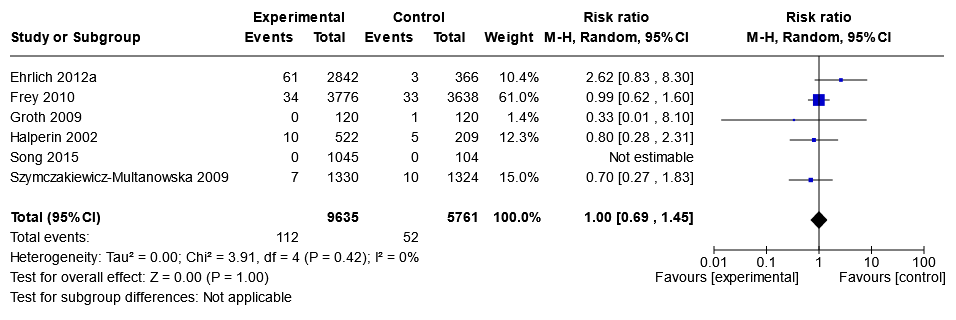


Suppl. Figure 44: Relative risk of fever after vaccination with cell-based influenza vaccine versus standard influenza vaccine (random-effects model)

Local adverse events

For pain at the injection site after vaccination, the primary review reported data from 5 RCTs, with a pooled RR of 1.22 (95%CI: 1.15 to 1.31, fixed effects model; random effects model: 1.19 (95%CI: 1.03 to 1.37) (Suppl. Figure 45-46). No additional data were identified in the update.


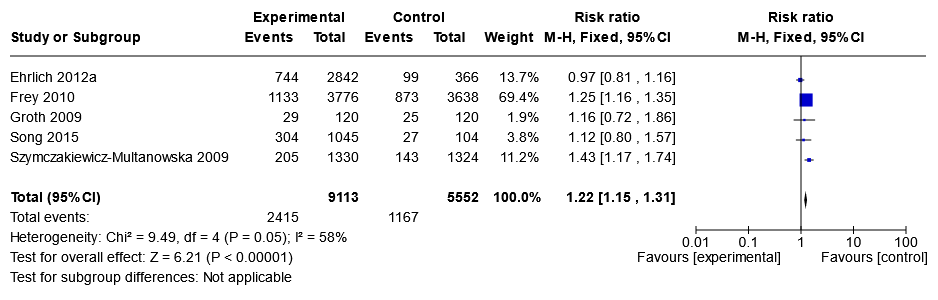


Suppl. Figure 45: Relative risk of pain after vaccination with cell-based influenza vaccine versus standard influenza vaccine (fixed-effects model)


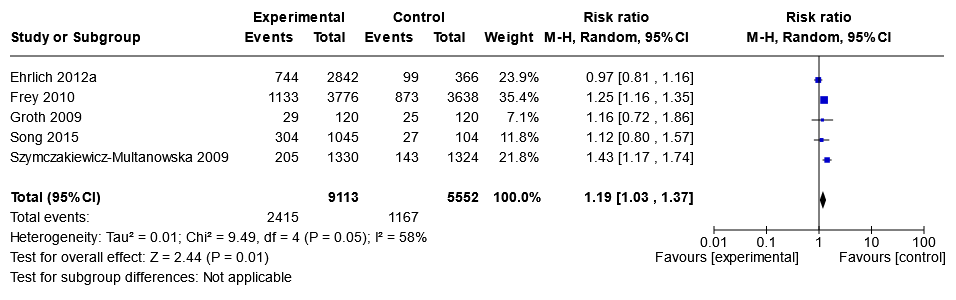


Suppl. Figure 46: Relative risk of pain after vaccination with cell-based influenza vaccine versus standard influenza vaccine (random-effects model)

Swelling at the injection site after vaccination was reported in 6 RCTs in the primary review. Using a fixed effects model, the RR was 1.15 (95%CI: 0.99 to 1.34), while the RR using a random effects model was 1.10 (95%CI: 0.88 to 1.37) (Suppl. Figure 47-48). No additional data were identified in the update.
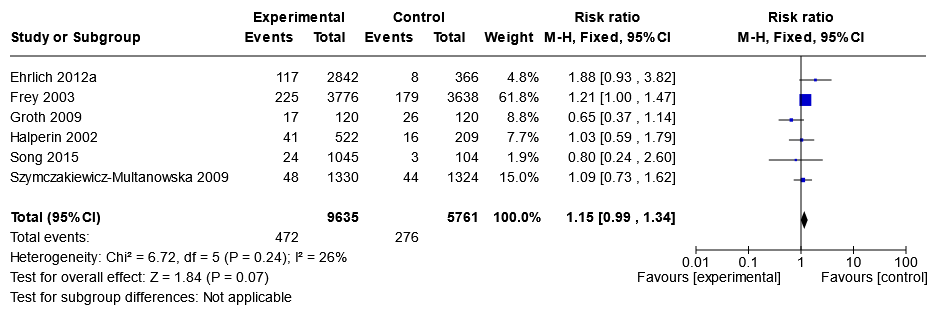


Suppl. Figure 47: Relative risk of swelling after vaccination with cell-based influenza vaccine versus standard influenza vaccine (fixed-effects model)


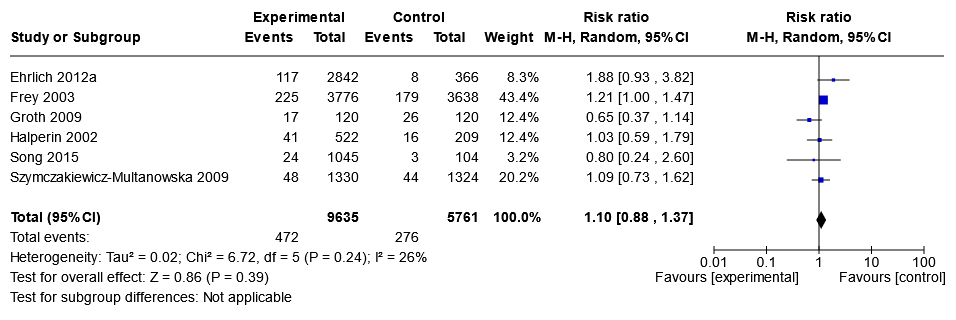


Suppl. Figure 48: Relative risk of swelling after vaccination with cell-based influenza vaccine versus standard influenza vaccine (random-effects model)

Adverse pregnancy outcomes after vaccination during pregnancy

No studies reported on this outcome, neither in the primary review nor in the update.

Adverse neonatal outcomes after vaccination during pregnancy

No studies reported on this outcome, neither in the primary review nor in the update.

### **Recombinant influenza vaccine**

*Secondary safety outcomes*

Systemic adverse events

Headache after administration of the recombinant vaccine was reported by 5 RCTs in the primary review. According to fixed effects model, pooled RR was 0.87 (95%CI: 0.76 to 1.01), while it was 0.80 (95%CI: 0.52 to 1.24) by random effects model (Suppl. Figure 49-50). No additional studies were identified in the update.
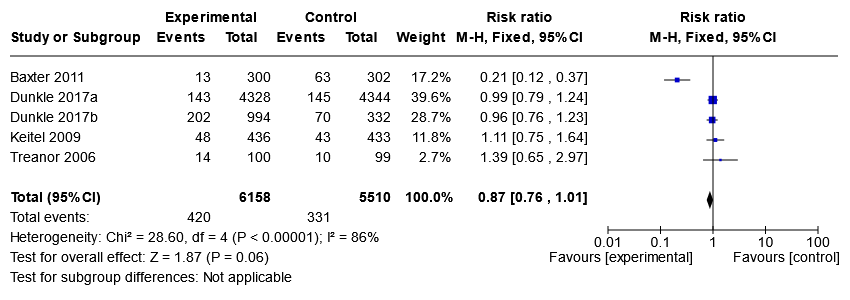


Suppl. Figure 49: Relative risk of headache after vaccination with recombinant influenza vaccine versus standard influenza vaccine (fixed-effects model)


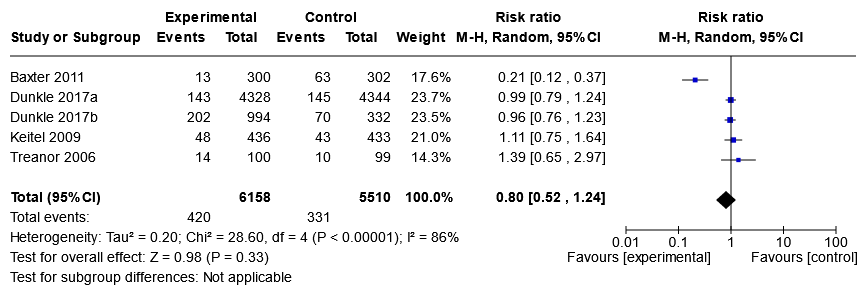


Suppl. Figure 50: Relative risk of headache after vaccination with recombinant influenza vaccine versus standard influenza vaccine (random-effects model)

In the primary review, no studies were included that reported on fever. In the update, we identified 2 NRSI which reported data on this outcome (42, 43). Both studies did not find an increased risk of fever (RR 0 (95%CI: 0 to 1.47) (43); RR inpatients: 0.38 (95%CI: 0.14 to 0.9); RR outpatients: 1.02 (95%CI: 0.6 to 1.74) (42).

Local adverse events

Seven RCTs were identified by the primary review that reported data on pain at the injection site. Pooled RR was 0.89 (95%CI: 0.84 to 0.95) by fixed effects model and 0.92 (95%CI: 0.84 to 1.00) by random effects model (Suppl. Figure 51-52). No additional data were identified in the update.


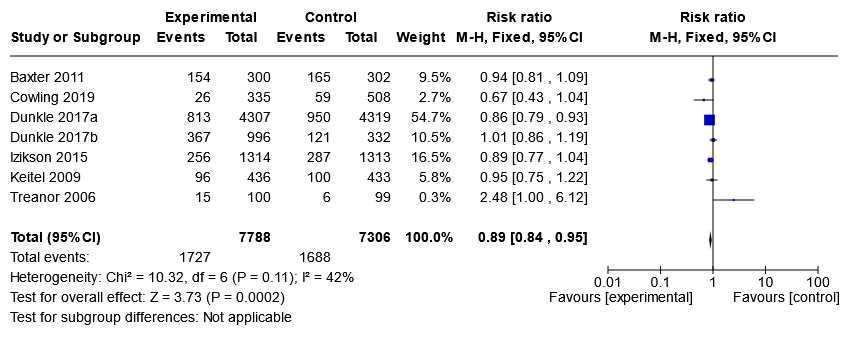


Suppl. Figure 51: Relative risk of pain after vaccination with recombinant influenza vaccine versus standard influenza vaccine (fixed-effects model)


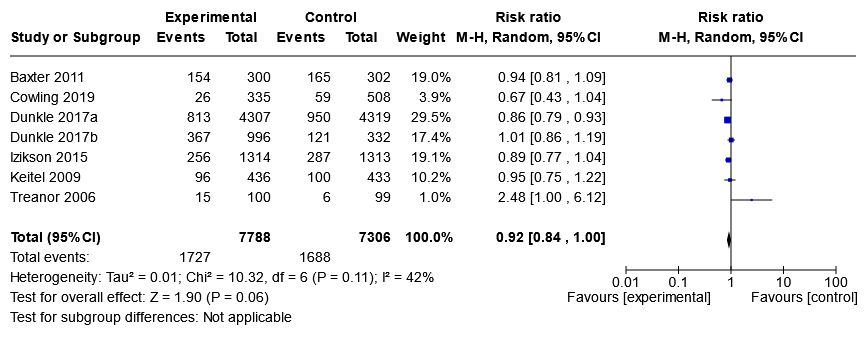


Suppl. Figure 52: Relative risk of pain after vaccination with recombinant influenza vaccine versus standard influenza vaccine (random-effects model)

Data on injection site swelling were provided by 6 RCTs in the primary review. According to the fixed effects model, pooled RR was 1.04 (95%CI: 0.87 to 1.24) and 0.94 (95%CI: 0.64 to 1.39) according to random effects model (Suppl. Figure 53-54). No additional data were identified in the update.
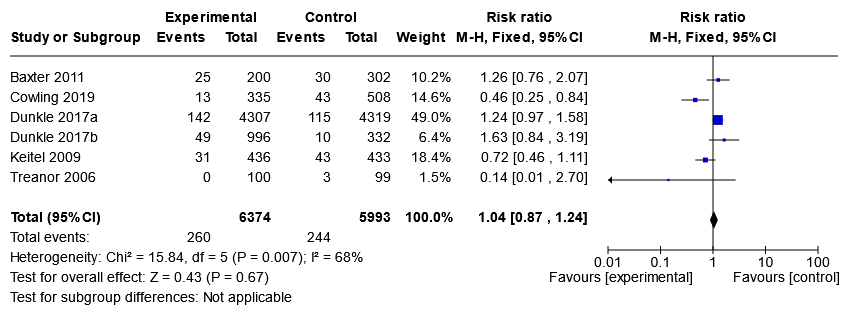


Suppl. Figure 53: Relative risk of swelling after vaccination with recombinant influenza vaccine versus standard influenza vaccine (fixed-effects model)


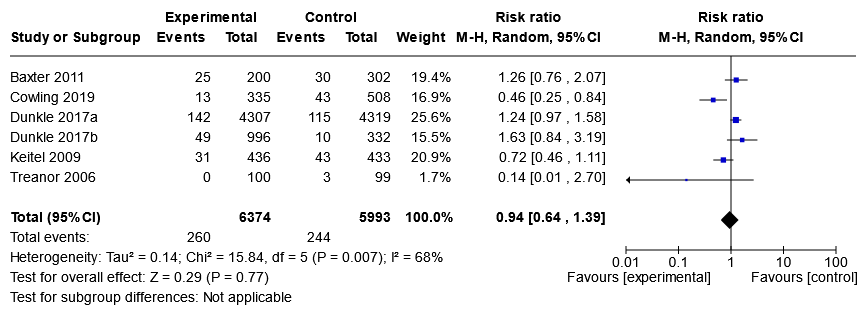


Suppl. Figure 54: Relative risk of swelling after vaccination with recombinant influenza vaccine versus standard influenza vaccine (random-effects model)

Adverse pregnancy outcomes after vaccination during pregnancy

No studies reported on this outcome, neither in the primary review nor in the update.

Adverse neonatal outcomes after vaccination during pregnancy

No studies reported on this outcome, neither in the primary review nor in the update.

# Appendix F – Summary of findings tables

Suppl. Table 1: Summary of findings relative effectiveness and safety of MF59-adjuvanted influenza vaccine vs. standard influenza vaccine in adults

| Outcome № of participants (studies) | Relative effect (95% CI) | **Anticipated absolute effects (95% CI)** | | | Certainty | What happens |
| --- | --- | --- | --- | --- | --- | --- |
|  |  | With standard influenza vaccine | MF59- adjuvanted influenza vaccine | **Difference** |  |  |
| Laboratory confirmed influenza  № of participants: 10.492 (7 observational studies) | **rVE-range:**  **-30**  (-146 to 31)  **to 88**  (51 to 100) | N. A. | N. A. | N.A. | ⨁⨁◯◯ Low^a,b^ | MF59-adjuvanted influenza vaccines may or may not reduce laboratory-confirmed influenza infection in adults compared to standard vaccine. |
| Influenza related hospitalisation (laboratory confirmed)  № of participants: 512 (1 observational study) | **rVE 59.2** (14.6 to 80.5) | N.A. | N.A. | N.A. | ⨁⨁⨁◯ Moderate^a^ | MF59-adjuvanted influenza vaccines probably reduce hospitalisation related to laboratory-confirmed influenza infection in adults compared to standard vaccine. |
| Influenza-related death (laboratory confirmed) | - | - | - | - | - | no data reported |
| Serious adverse event (SAE)  № of participants: 8504 (3 RCTs) | **RR 0.95** (0.19 to 4.72) | 0.1% | **0.1%** (0 to 0.3) | **0.0% fewer** (0.1 fewer to 0.3 more) | ⨁⨁◯◯ Low^c,d^ | MF59-adjuvanted influenza vaccines may result in little to no difference in serious adverse events (SAEs) compared to the standard vaccine. |
| Idiopathic thrombocytopenic purpura | - | - | - | - | - | no data reported |
| Narcolepsy/cataplexy | - | - | - | - | - | no data reported |
| Guillain–Barré syndrome (GBS) | - | - | - | - | - | no data reported |
| ***The risk in the intervention group** (and its 95% confidence interval) is based on the assumed risk in the comparison group and the **relative effect** of the intervention (and its 95% CI). **CI:** confidence interval; **RR:** risk ratio; **rVE**: relative vaccine effectiveness [(1 – Risk Ratio) *100%] | | | | | | |
| **GRADE Working Group grades of evidence** **High certainty:** we are very confident that the true effect lies close to that of the estimate of the effect. **Moderate certainty:** we are moderately confident in the effect estimate: the true effect is likely to be close to the estimate of the effect, but there is a possibility that it is substantially different. **Low certainty:** our confidence in the effect estimate is limited: the true effect may be substantially different from the estimate of the effect. **Very low certainty:** we have very little confidence in the effect estimate: the true effect is likely to be substantially different from the estimate of effect. | | | | | | |
| Explanations a. Residual confounding cannot be excluded  b. Heterogeneous point estimates between the studies.  c. High risk of bias in 2 out of 3 studies.  d. Wide confidence interval. | | | | | | |

Suppl. Table 2: Summary of findings relative effectiveness and safety of high-dose influenza vaccine vs. standard influenza vaccine in adults

| Outcome № of participants (studies) | Relative effect (95% CI) | **Anticipated absolute effects (95% CI)** | | | Certainty | What happens |
| --- | --- | --- | --- | --- | --- | --- |
|  |  | With standard influenza vaccine | High-dose influenza vaccine | Difference |  |  |
| Laboratory confirmed influenza (lab confirmed) assessed with: PCR № of participants: 31989 (1 RCT) | **rVE 24**  **(11 to 36)** | 1.9% | **1.4**% (1.2 to 1.7) | **0.5**% **fewer** (0.7 fewer to 0.2 fewer) | ⨁⨁⨁◯ Moderate^a^ | High-dose influenza vaccines probably slightly reduce laboratory-confirmed influenza infection in adults. |
| Influenza related hospitalisation (laboratory confirmed) assessed with: PCR № of participants: 1107 (1 NRSI) | **rVE 27** (-1 to 48) | N.A. | N.A. | N.A. | ⨁⨁◯◯ Low^b,c^ | High-dose influenza vaccines may slightly reduce hospitalisation related to laboratory-confirmed influenza infection in adults. |
| Influenza-related death (laboratory confirmed) | - | - | - | - | - | no data reported |
| Serious adverse events (SAE) № of participants: 9034 (6 RCTs) | **RR 1.02** (0.42 to 2.46) | 0.2% | **0.2**% (0.1 to 0.6) | **0.0**% **fewer** (0.1 fewer to 0.4 more) | ⨁⨁◯◯ Low^c,d^ | High-dose influenza vaccines may result in little to no difference in serious adverse events (SAEs) related to vaccination. |
| Idiopathic thrombocytopenic purpura | - | - | - | - | - | no data reported |
| Narcolepsy/cataplexy | - | - | - | - | - | no data reported |
| Guillain–Barré syndrome (GBS) | - | - | - | - | - | no data reported |
| ***The risk in the intervention group** (and its 95% confidence interval) is based on the assumed risk in the comparison group and the **relative effect** of the intervention (and its 95% CI). **CI:** confidence interval; **RR:** risk ratio; **rVE**: relative vaccine effectiveness [(1 – Risk Ratio) *100%] | | | | | | |
| **GRADE Working Group grades of evidence** **High certainty:** we are very confident that the true effect lies close to that of the estimate of the effect. **Moderate certainty:** we are moderately confident in the effect estimate: the true effect is likely to be close to the estimate of the effect, but there is a possibility that it is substantially different. **Low certainty:** our confidence in the effect estimate is limited: the true effect may be substantially different from the estimate of the effect. **Very low certainty:** we have very little confidence in the effect estimate: the true effect is likely to be substantially different from the estimate of effect. | | | | | | |
| Explanations a. One RCT with moderate risk of bias.  b. Residual confounding can not be excluded.  c. Wide confidence interval.  d. 3 out of 6 studies moderate risk of bias. | | | | | | |

Suppl. Table 3: Summary of findings relative effectiveness and safety of cell-based influenza vaccine vs. standard influenza vaccine in adults

| Outcome № of participants (studies) | Relative effect (95% CI) | **Anticipated absolute effects (95% CI)** | | | Certainty | What happens |
| --- | --- | --- | --- | --- | --- | --- |
|  |  | With standard influenza vaccine | With cell-based influenza vaccine | **Difference** |  |  |
| Laboratory confirmed influenza (lab confirmed) assessed with: PCR № of participants: 1,025,097  (2 observational studies) | **rVE-range**  **-5.8**  (-36.1 to 17.7)  **to 21.4**  (-7.3 to 42.4) | N.A. | N.A. | N.A. | ⨁⨁◯◯ Low^a^ | Cell-based influenza vaccines may or may not reduce laboratory-confirmed influenza infection in adults. |
| Influenza related hospitalisation (laboratory confirmed) assessed with: PCR № of participants: 1741  (1 observational study) | **rVE 8.5** (-75.9 to 52.3) | N.A. | N.A. | N.A. | ⨁⨁◯◯ Low^a,b^ | Evidence is uncertain, whether cell-based influenza vaccines reduce hospitalisation related to laboratory-confirmed influenza infection in adults. |
| Influenza-related death (laboratory confirmed) | - | - | - | - | - | no data reported |
| Serious adverse events (SAE) № of participants: 3208 (1 RCT) | **RR 0.39** (0.02 to 9.49) | N.A. | N.A. | N.A. | ⨁⨁◯◯ Low^b^ | Cell-based influenza vaccines may or may not decrease serious adverse events (SAEs) related to vaccination. |
| Idiopathic thrombocytopenic purpura | - | - | - | - | - | no data reported |
| Narcolepsy/cataplexy | - | - | - | - | - | no data reported |
| Guillain–Barré syndrome (GBS) | - | - | - | - | - | no data reported |
| ***The risk in the intervention group** (and its 95% confidence interval) is based on the assumed risk in the comparison group and the **relative effect** of the intervention (and its 95% CI). **CI:** confidence interval; **RR:** risk ratio; **rVE**: relative vaccine effectiveness [(1 – Risk Ratio) *100%] | | | | | | |
| **GRADE Working Group grades of evidence** **High certainty:** we are very confident that the true effect lies close to that of the estimate of the effect. **Moderate certainty:** we are moderately confident in the effect estimate: the true effect is likely to be close to the estimate of the effect, but there is a possibility that it is substantially different. **Low certainty:** our confidence in the effect estimate is limited: the true effect may be substantially different from the estimate of the effect. **Very low certainty:** we have very little confidence in the effect estimate: the true effect is likely to be substantially different from the estimate of effect. | | | | | | |
| Explanations a. Residual confounding can not be excluded.  b. Wide confidence interval. | | | | | | |

Suppl. Table 4: Summary of findings relative effectiveness and safety of recombinant influenza vaccine vs. standard influenza vaccine in adults

| Outcome № of participants (studies) | Relative effect (95% CI) | **Anticipated absolute effects (95% CI)** | | | Certainty | What happens |
| --- | --- | --- | --- | --- | --- | --- |
|  |  | With standard influenza vaccine | With recombinant influenza vaccine | Difference |  |  |
| Laboratory confirmed influenza (lab confirmed) assessed with: PCR № of participants: 8855 (1 RCT) | **rVE 30** (10 to 47) | **3.1%** | **2.2%** (1.7 to 2.8) | **0.9% fewer** (1.5 fewer to 0.3 fewer) | ⨁⨁⨁◯ Moderate^a^ | Recombinant influenza vaccines probably slightly reduce laboratory-confirmed influenza infection in adults. |
| Influenza related hospitalisation (laboratory confirmed) assessed with: PCR № of participants: 1,630,328  (1 RCT) | - | Certainty of the evidence could not be assessed due to lack of information. | | - | - | N.A. |
| Influenza-related death (laboratory confirmed) | - | - | - | - | - | no data reported |
| Serious adverse events (SAE) № of participants: 907 (2 RCTs) | **RR 3.04** (0.32 to 29.10) | N.A. | N.A. | N.A. | ⨁⨁◯◯ Low^b^ | Recombinant influenza vaccines may or may not result in an increase in serious adverse events (SAEs) related to vaccination. |
| Idiopathic thrombocytopenic purpura № of participants: 42.684  (1 observational study) | **OR 0.52** (0.15 to 1.50) | N.A. | N.A. | N.A. | ⨁⨁◯◯ Low^c,d^ | Recombinant influenza vaccines may or may not result in an decrease in of idiopathic thrombocytopenic purpura related to vaccination. |
| Narcolepsy/cataplexy № of participants: 305.659  (1 observational study) | **OR 0** (0 to 6) | N.A. | N.A. | N.A. | ⨁◯◯◯ Very low^d,e^ | Evidence is uncertain about the effect of recombinant influenza vaccines on narcolepsy/cataplexy related to vaccination. |
| Guillain–Barré syndrome (GBS) № of participants: 305.659  (1 observational study) | **OR 0.00** (0.00 to 16.07) | N.A. | N.A. | N.A. | ⨁◯◯◯ Very low^d,e^ | Evidence is uncertain about the effect of recombinant influenza vaccine on Guillain–Barré syndrome related to vaccination. |
| ***The risk in the intervention group** (and its 95% confidence interval) is based on the assumed risk in the comparison group and the **relative effect** of the intervention (and its 95% CI). **CI:** confidence interval; **OR:** odds ratio; **RR:** risk ratio; **rVE**: relative vaccine effectiveness [(1 – Risk Ratio) *100%] | | | | | | |
| **GRADE Working Group grades of evidence** **High certainty:** we are very confident that the true effect lies close to that of the estimate of the effect. **Moderate certainty:** we are moderately confident in the effect estimate: the true effect is likely to be close to the estimate of the effect, but there is a possibility that it is substantially different. **Low certainty:** our confidence in the effect estimate is limited: the true effect may be substantially different from the estimate of the effect. **Very low certainty:** we have very little confidence in the effect estimate: the true effect is likely to be substantially different from the estimate of effect. | | | | | | |

#### Explanations

a. One RCT with moderate risk of bias.

b. Two RCTs with moderate risk of bias.

c. Residual confounding can not be excluded.

d. Wide confidence interval.

e. No adjustment for co-morbidities, even though there was a significant difference between the groups.

# Appendix G – Differences to study protocol

There are several modifications between the study protocol and the actual review.

- Literature search was restricted to Medline and Embase.
- Metanalysis was performed using RevMan Web and Mantel-Haenszel method was used.
- For detection of possible publication bias (small study effects) visual inspection funnel plots were used.
- In order to not undermine the systematic character of this review, personal communication with investigators was only used for clarification of published study data. The PROSPERO protocol was changed accordingly on the 4 December 2023.
